# Preparation and DFT studies of chiral Cu (I)-complexes of biphenyl bisoxazolines and their application in enantioselective Kharasch–Sosnovsky reaction

**DOI:** 10.1038/s41598-022-18922-1

**Published:** 2022-09-03

**Authors:** Saadi Samadi, Hamid Arvinnezhad, Sirwan Mansoori, Hadi Parsa

**Affiliations:** Laboratory of Asymmetric Synthesis, Department of Chemistry, Faculty of Science, University of Kurdistan, Sanandaj, 66177-15175 Iran

**Keywords:** Asymmetric catalysis, Homogeneous catalysis, Organic chemistry, Stereochemistry, Asymmetric synthesis

## Abstract

Effect of a range of *t*-butyl perbenzoates bearing electron-withdrawing and electron-donating substitutions on the phenyl ring and HZSM-5 as a porous additive at 0 °C in enantioselective allylic C–H bond oxidation of cyclic and acyclic olefins in the presence of Cu (I)-(*S,*a*S,S*) complexes of biphenyl bisoxazoline ligands, produced easily through the chelation-induced process, were investigated. The enantioenriched allylic esters were obtained in reasonable times with excellent enantioselectivities and yields using electron-withdrawing substituted peresters in the presence of Cu (I)-(*S,*a*S,S*)**-1a** complex, containing phenyl groups at the stereogenic centers of the oxazoline moieties. To reach a better insight on geometry, chemical activity, enantioselectivity, and thermodynamic stability of the Cu (I)-BOX complexes, DFT calculations with B3LYP-D3/6-31G (*d*, *p*) level of theory were applied to them. Moreover, NBO analysis was used to illustrate interactions between orbitals.

## Introduction

**Scheme 1:**
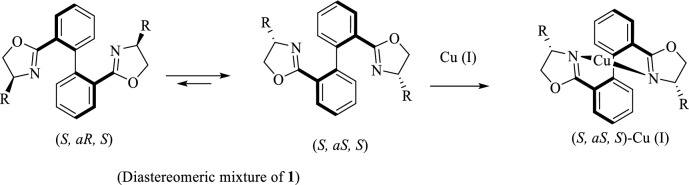
Resolution of (*S,*a*S,S*)-bipheny bisoxazoline complex through chelation-induced process.

Over the last two decades, chiral bisoxazoline (BOX) ligands, mainly prepared from the reaction of various dicarboxylic acids and a range of chiral *β*-amino alcohols, have great attracted increasing attention as promising ligands in numerous catalytic asymmetric transformations^1–11^. It has been shown that an effective arrangement for high asymmetric induction can be achieved owing to the existence of stereogenic centers adjacent to the active catalytic site. Furthermore, both great diversity in amino alcohols and diacid structures lead to a wide range of BOX ligands with unique features. On the other hand, literature surveys have implied that the introduction of an additional chiral element in the ligand backbone is capable of effectively controlling asymmetric induction^12–17^. It has demonstrated that the combination of a biaryl backbone and chiral oxazoline rings at *ortho-*position leads to a mixture of atropisomeric diastereomers of BOX ligands bearing a chiral axis close to the stereogenic centers of the oxazoline moieties^18–25^. However, separation of the diastereomers is always a tedious and time-consuming process, and also the maximum theoretical yield of the resulting atropisomers is only 50%. These drawbacks can be ingeniously overcome by using a biphenyl backbone containing only two *ortho* oxazoline moieties. Such a BOX ligand scaffold exists as an equilibrium mixture of two axis-unfixed atropisomers that are able to readily rotate around C–C bond between Ph–Ph, and one of them tend to be selectively coordinated to a metal ion such as Cu (I), Ag (I), Pd (II), and Zn (II) through chelation-induced process^12–15,26^. It is obvious that during such a dynamic kinetic resolution process, the more stable BOX complex can be obtained in a theoretical yield of 100% due to rotation around the biphenyl axis. According to our earlier study^15^ and also Ikeda group^12,14^, it was found that the selective complexation of an equilibrium mixture of biphenyl bisoxazoline **1** with Cu (I) results in the Cu (I)-(*S,*a*S,S*) complex as almost the only complex that can catalyze allylic oxidation and cyclopropanation with high enantioselectivity (Scheme 1).

Enantioselective copper-catalyzed allylic C-H bond oxidation of olefins with peresters (Kharasch–Sosnovsky reaction) has been known as a powerful strategy to prepare chiral allylic esters by introducing a new stereogenic center containing oxygen substituent close to an intact C=C^27–49^. Previous studies have shown that this asymmetric transformation is considerably improved by inorganic porous additives^15,18,50^. Therefore, based on the above-mentioned and in continuation of our research group studies to tune the best condition^15,18,49–52^, enantioselective allylic C–H bond oxidation of alkenes was evaluated with different substituted *t*-butyl perbenzoates as an oxidant in the presence of inorganic porous additives, using the copper complexes of biphenyl BOX ligands **1** bearing phenyl and *iso*propyl groups at the stereogenic centers of the oxazoline moiety. Furthermore, to evaluation the thermodynamic stability and catalyst activity of the chiral biphenyl BOX Cu-complexes, density functional theory (DFT) calculations were performed.

## Result and discussion

**Scheme 2:**
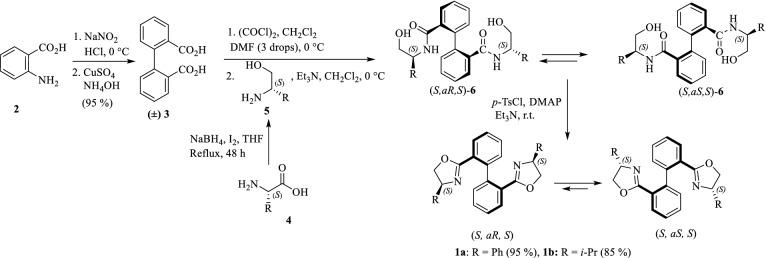
Preparation of bipheny bisoxazoline ligands **1.**

Chiral atropisomeric BOX ligands **1a** and **1b** were prepared according to our previous study^15^. In this procedure, at first, inexpensive starting material anthranilic acid **2** was converted to biphenyl dicarboxylic acid backbone **3** using Cu (I) through homo-coupling of aryldiazonium salts^53^. Treatment of the obtained dicarboxylic acid **3** with oxalyl chloride using a catalytic amount of DMF resulted in diacyl chloride that was then reacted with two individual (*S*)-amino alcohol **5a** and **5b**, prepared from the reduction of the corresponding chiral amino acids **4a** and **4b**^54^, to form (*S,aS,S*) and (*S,aR,S*)-bishydroxylamides **6a** and **6b**. As expected, the *S,aS,S* isomer^15^, the more stable one, is mainly formed during the crystallization of the equilibrium mixture. It seems that this process, which is known as crystallization induced asymmetric transformation^55^, is mainly directed by hydrogen bonding. Cyclization of the bishydroxylamides **6a** and **6b** using *p*-TsCl, DMAP, and Et_3_N at ambient temperature resulted in an equilibrium mixture of the (*S*,a*S*,*S*) and the (*S*,a*R*,*S*) atropisomeric ligands **1a** and **1b** in 95% and 85% yields, respectively (Scheme 2).

The ^1^HNMR spectra of the resulting ligands have demonstrated two sets of signals for the rotatory diastereomers with different ratios. In the case of the ligand bearing phenyl groups (**1a**), the observed ratio of (*S*,a*S*,*S*) to (*S*,a*R*,*S*) was 61:39, while for the ligand bearing *iso*propyl groups (**1b**), it was 80: 20. These ratios indicate that one of the two possible diastereomers has more preference to the other, which can be attributed to the steric congestion of the R groups on the oxazoline rings. On the basis of the behavior of such scaffolds, in order to obtain enantiomerically pure complexes, Cu(CH_3_CN)_4_PF_6_ was added to the conformational mixture. Upon addition of the Cu salt, one of the both possible diastereomeric complexes, the (*S*,a*S*,*S*) isomer, was predominantly formed through the chelation-induced process. It can be deduced that one of the two diastereomers converts to another by rotation around the internal C–C bond, resulting in a stable Cu-isomeric complex. In the ^1^H NMR spectrum of the obtained complex, one set of signals was mainly observed. Comparison of the ^1^H NMR spectra of the Cu (I)-**1a** complex with the ligand **1a**, for example, clearly showed that three of six triplets signals in the ligand **1a**, which are around at δ = 3.83–5.27 ppm, were disappeared. Such a distinct decline in the number of signals was also observed in the ^13^CNMR spectra of the Cu (I)-**1a** complex. Although the absolute configuration of the resulting complex has been determined as (*S,aS,S*) by NOE^14,15^, attempts to achieve suitable crystals for X-ray single crystallographic analysis to assign the exact stereochemistry were failed.

**Figure 1:**
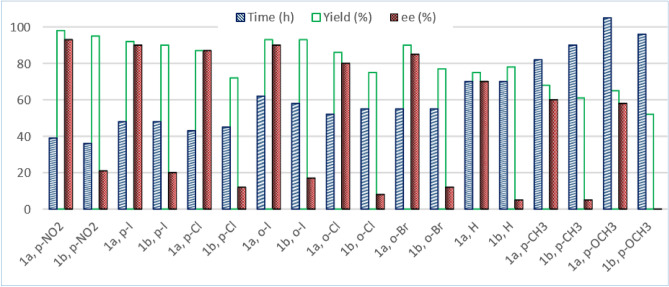
Comparison of the chiral ligands **1a** and **1b** in *ee*, yield and reaction time.

The resulting Cu-(*S,*a*S,S*)-BOX complexes **1a** and **1b** were evaluated in the enantioselective allylic oxidation of a range of olefins using different substituted *t*-butyl perbenzoate **7** bearing both electron-withdrawing and electron-donating groups on the phenyl ring. To obtain the optimum condition, the asymmetric reaction was studied by employing cyclohexene as the substrate and *t*-butyl *p*-nitroperbenzoate **7a** in the presence of catalytic amounts of Cu-**1a** or **1b** complexes, which is generated in situ from a slight excess of ligands **1a** or **1b** and a variety of copper (I) and (II) salts such as Cu(CH_3_CN)_4_PF_6,_ CuOTf, Cu(OTf)_2_, CuI, CuCl_2_, CuO, CuSO_4_, Cu(OAc)_2_, Cu_2_O, and Cu(NO_3_)_2_. The reaction was also examined in a range of temperatures from − 10 to room temperature in different polar to non-polar solvents such as acetonitrile, acetone, chloroform, dichloromethane, toluene, and *n*-hexane. Moreover, the effect of additives such as phenylhydrazine as a reductant agent that reduces Cu (II) into Cu (I), and also inorganic porous materials such as molecular sieves 4 Å, MCM-41, SBA-15 and HZSM-5 were investigated. The results showed that the corresponding enantiomerically enriched allylic ester **8a**, (*S*)-2-cyclohexenyl-*p*-nitrobenzoate, could be obtained in high enantioselectivity (93% *ee*) and excellent yield (98%) in a reasonable time (39 h) when the reaction was carried out at room temperature in CH_3_CN, in the presence of 3.2 mol% of Cu(CH_3_CN)_4_PF_6_-**1a** complex as a catalyst, 5 *μ*L phenylhydrazine and 5 mg HZSM-5 (Table 1, entry 1). As it can be seen in Table 1 and Fig. 1, conducting the reaction in the presence of Cu(CH_3_CN)_4_PF_6_-**1a** gave higher enantioselectivites than Cu(CH_3_CN)_4_PF_6_-**1b**. It was also found that the best results could be achieved when the peresters containing electron-withdrawing substitutions, especially *p*-nitro (**7a**), *p*-iodo (**7b**), and also *o*-iodo (**7d**) substitutions, were used (Table 1, entries 1–6). The reaction using electron donating groups such as Me (**7 h**) and OMe (**7i**) was slower and gave allylic esters in lower yields and *ee* values (Table 1, entries 8 and 9).

**Table 1:**
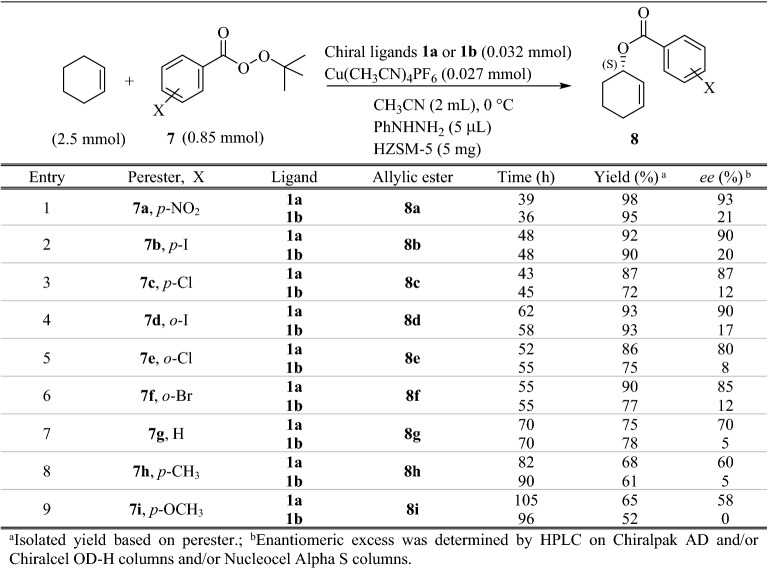
Effect of stereochemistry of the BOX ligand and substitution on the perester on asymmetric allylic oxidation of cyclohexene.

**Scheme 3:**
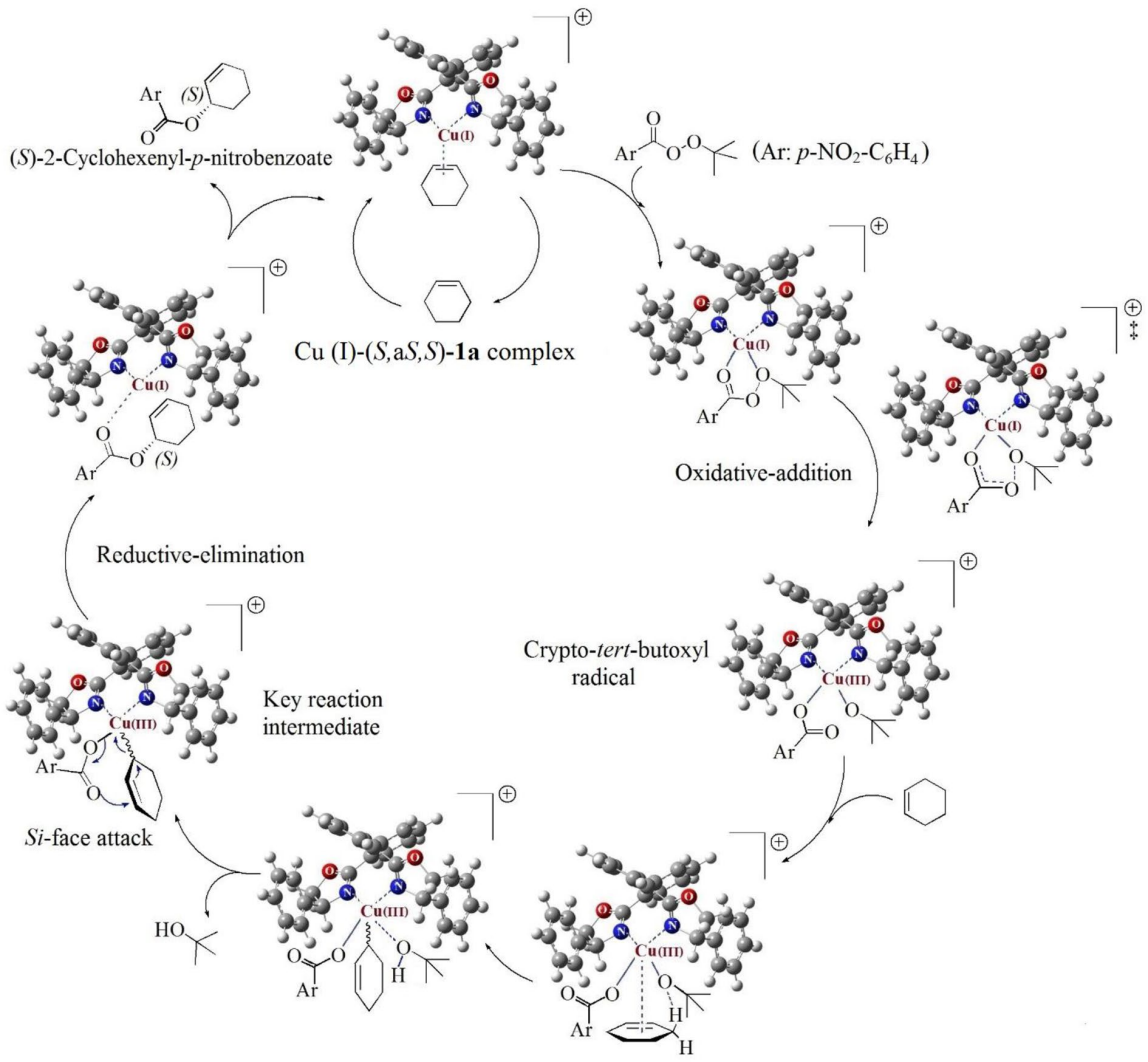
A rational mechanism of enantioselective allylic oxidation of cyclohexene using *tert*-butyl *p*-nitrobenzoperoxoate, catalyzed by Cu (I)-(*S,*a*S,S*)-**1a** complex.

According to literature^49,56–65^, the proposed reaction mechanism take places by complexation of the (*S,*a*S,S*)-biphenyl BOX ligand **1a** with Cu (I) to form the chiral catalyst, which is assumed that mainly coordinated with cyclohexene (Scheme 3). Addition of the *t*-butyl *p*-nitroperbenzoate **7a** initiates the reaction through a catalytic cycle involving change in the copper oxidation state. In the first step, cyclohexene is substituted by the perester **7a**. Following that, in the oxidative-addition step, crypto-*tert*-butoxyl radical is formed through concerted cleavage of oxygen–oxygen bond of the perester **7a** and also oxidation of copper (I) into copper (III). In the next step, cyclohexene again coordinates to the copper, and then, in the limiting step, the *tert*-butoxo group, which is bonded to the copper complex, selectively removes a prochiral allylic hydrogen of the cyclohexene in an intramolecular process. Thereafter, elimination of a *tert*-butyl alcohol leads to the key intermediate. Subsequently, carboxyl attacks the *Si*-face of cyclohexnyl and through a pericyclic rearrangement including the migration of π-bond and a stereospecific reductive-elimination, gives (*S*)-2-cyclohexenyl-*p*-nitrobenzoate-catalyst complex. Eventually, enantioenriched (*S*)-2-cyclohexenyl-*p*-nitrobenzoate is released by replacing with cyclohexene, and as a result, the Cu (I)- cyclohexene is reproduced.

It was observed that the reaction rate in the case of *ortho* substituted peresters was slower than *para*; however, enantioselectivities and yields were not significantly different (Table 1, *cf*. entries 1–3 and 4–6). Therefore, under optimum conditions, allylic oxidation of other olefinic substrates with peresters **7a**,** b** and **d** were also investigated. Similarly, ligand **1a** gave better results than **1b**, and also *p*-nitroperbenzoate **7a** was the best oxidant (Table 2, entries 1–9). This discrepancy in the enantioselectivity may be attributed to the interaction between generated allyl radicals and the phenyl substituents in ligand **1a** at the transition states^51,66^. In case of acyclic olefins, the reaction times were longer, and yields and *ee* values were also inferior, although the best results were obtained in the presence of *p*-iodoperbenzoate **7b** (Table 2, entries 10–12). It seems that due to more conformational flexibility of the acyclic olefins compared with the cyclic ones, both *re*-face and *Si*-face can be attacked^32^. Cyclopentene and cyclooctene afforded the corresponding enantioenriched allylic esters **9** and **10** in longer times with lower enantioselectivities and yields in comparison to cyclohexene (Table 2, entries 1–6). However, in the case of 1,5-cyclooctadiene the best results were obtained. In other words, not only the reaction was completed in shorter times, but enantioselectivity and yield of the obtained chiral ester **11** were also excellent (Table 2, entries 7–9).

**Table 2:**
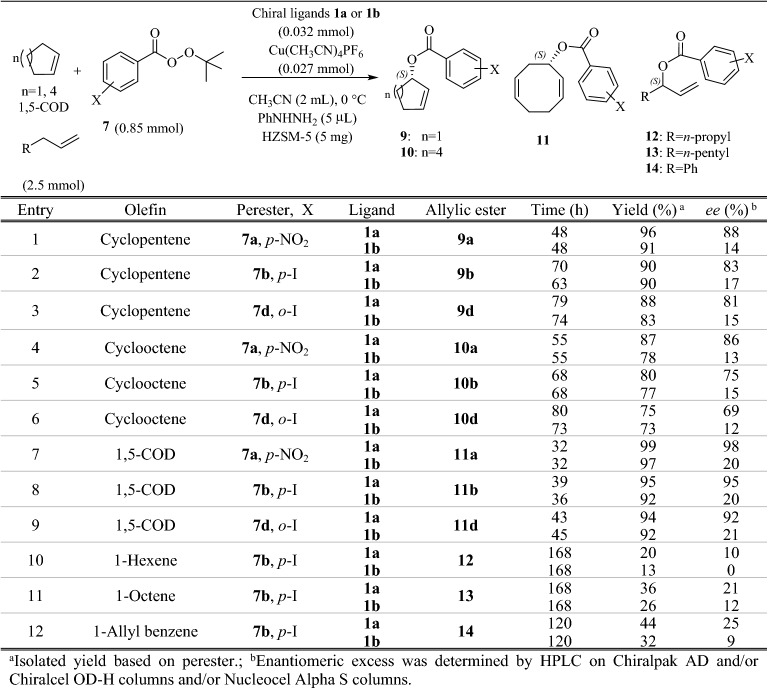
Enantioselective allylic oxidation of cyclic and acyclic olefins using BOX ligands **1a** and **1b.**

**Figure 2:**
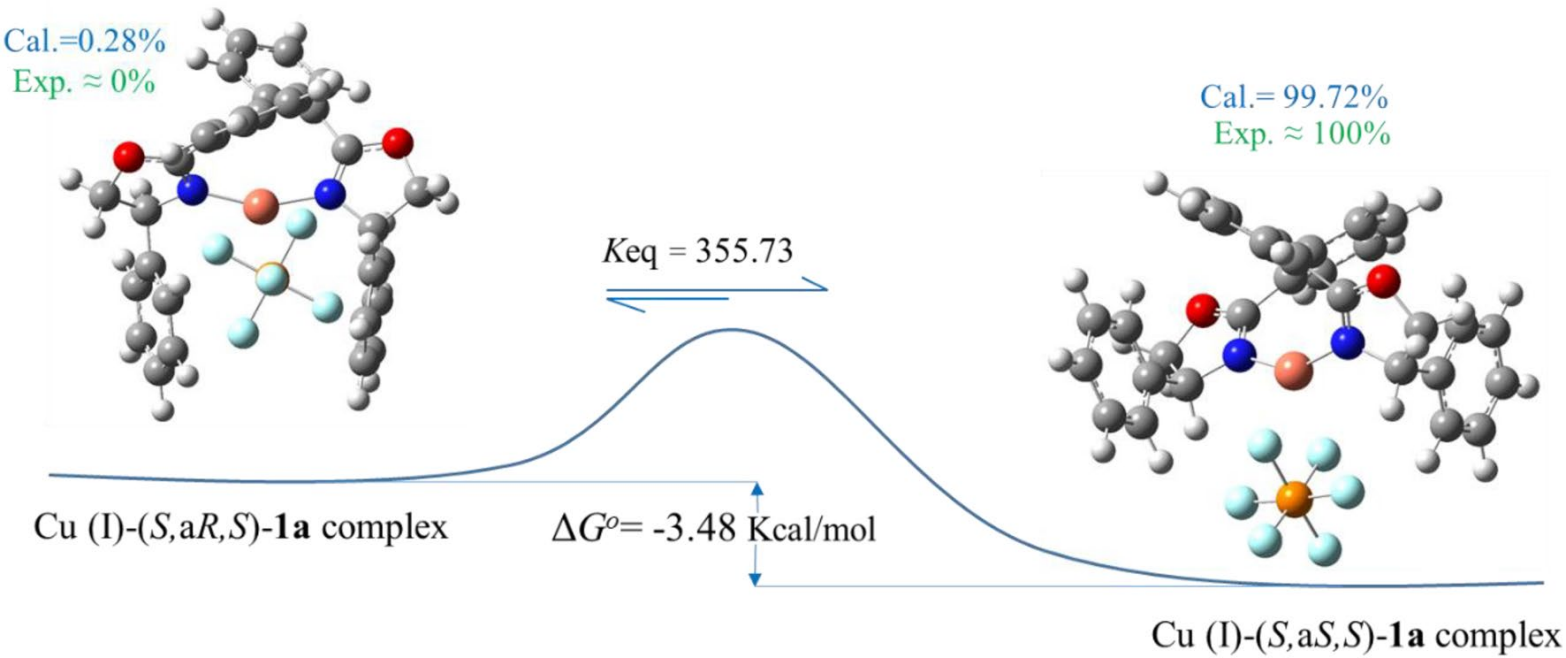
Gibbs free energy and equilibrium constant values for Cu (I)-**1a** complexes.

In the following, to gain a greater insight in to the structure of the copper complexes **1a** and **1b**, catalytic activity, enantioselectivity, thermodynamic stability, and interactions between orbitals, DFT calculations were carried out using the B3LYP method at 6–31 (*d*,*p*) basis set level and CPCM as the method of solvent. Moreover, the Van der Waals interactions were considered to correct DFT energies. A suitable way to investigate the thermodynamic stability of isomers is the comparison between their Gibbs free energy values^67–69^. Thermochemistry results of the isomeric complexes containing phenyl group (Cu (I)-**1a**) showed that the Cu (I)-(*S,*a*S,S*)-isomeric complex has more negative free energy by 3.48 kcal/mol than the (*S,*a*R,S*) isomer, which means that the (*S,*a*S,S*)-complex is more stable than another one. Moreover, the equilibrium constant, which indicates the population of each isomer, was calculated to be *K* = 355.73, at ∆*G*^*o*^ = − 3.48 kcal/mol that again confirms the more preferred of the (*S,*a*S,S*) isomeric complex than the (*S,*a*R,S*) isomer (Fig. 2). Similarly, the difference Gibbs free energy between the isomeric complexes containing *iso*propyl group (Cu (I)-**1b**) displayed that the (*S,*a*S,S*) complex is 3.05 kcal/mol more negative than another one, and the equilibrium constant at ∆*G*^*o*^ = − 3.05 kcal/mol is 172.15, which again showed that the (*S*,a*S*,*S*) complex is almost the only formed product (Figure S46). All of these results are in good agreement with the experimental results, where the complexation of the mixture of the free ligands with Cu(CH_3_CN)_4_PF_6_ showed mainly one set of signals in the ^1^HNMR and ^13^CNMR spectra. Based on the NMR observations, the absolute configuration of the main complex was assigned as (*S,*a*S,S*)^14,15^.

**Figure 3:**
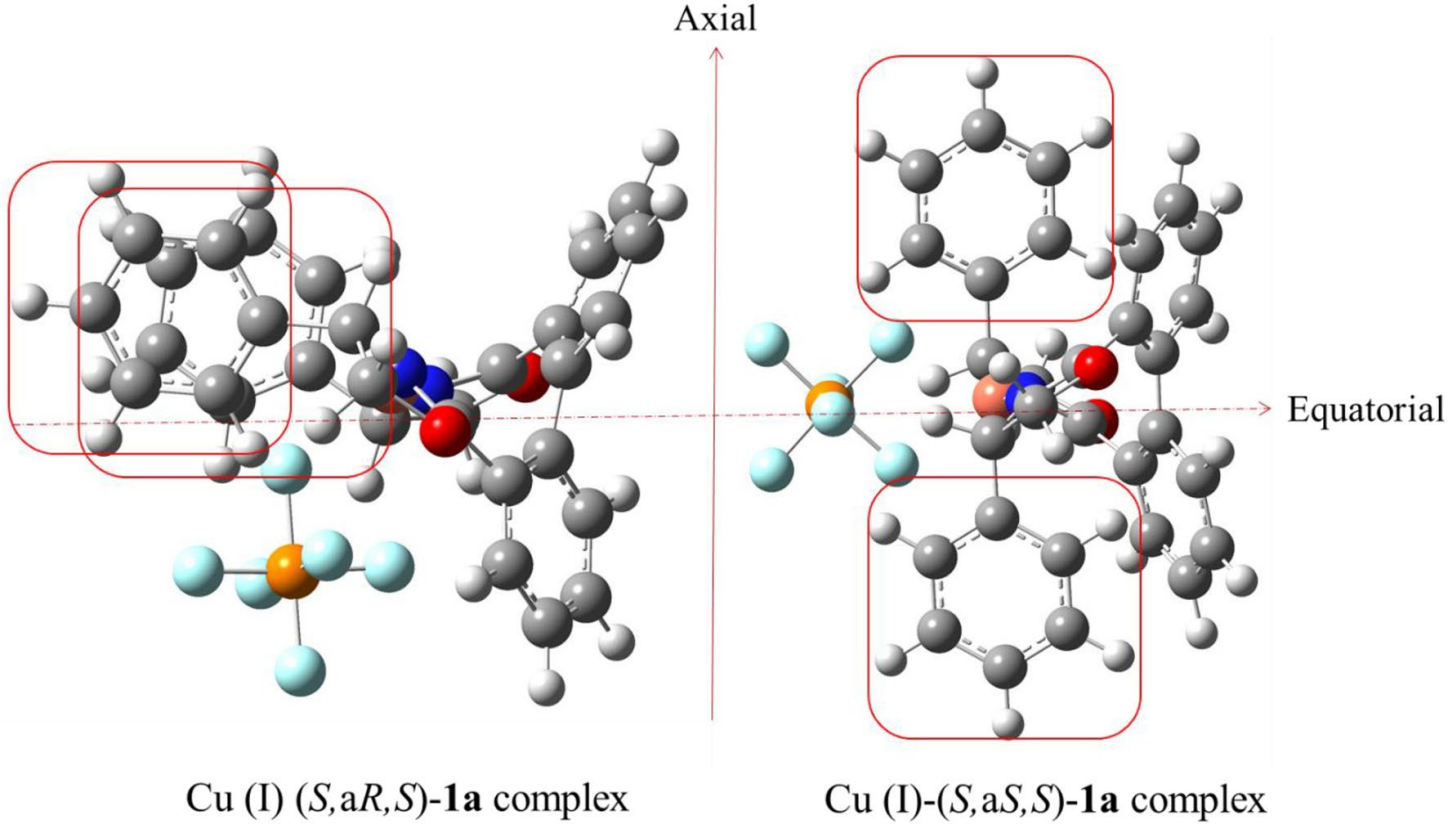
Optimized structures of the Cu (I)*-*(*S,*a*S,S*) and (*S,*a*R,S*)-**1a** complexes in CHCl_3_.

It was calculated that the relative population of the (*S,*a*S,S*) isomeric complexes Cu (I)-**1a** and Cu (I)-**1b** are 99.72% and 99.42%, respectively (Fig. 2 and S46). It can be deduced that regardless of the type of substitution at the stereogenic centers, the Cu (I)-(*S,*a*S,S*) diastereomer is more stable than the other one. The optimized geometric structures of the Cu (I)-(*S,*a*S,S*)**-1a** and Cu (I)-(*S,*a*R,S*)**-1a** have obviously shown that the (*S,*a*R,S*)-isomer confronts with steric hindrance caused by phenyl groups, which are in the equatorial position and have eclipsed form to each other. It is thought that the eclipsed form does not allow the PF_6_^−^ group to be at the best distance and orientation from the Cu (I), thus the Cu (I)-(*S,*a*R,S*)**-1a** complex has more energy level than the (*S,*a*S,S*) isomer (Fig. 3). On the contrary, in the Cu (I)-(*S,*a*S,S*)**-1a**, the phenyl substitutions are placed in the axial position and *anti*-form to each other, so, due to lack of constraint, the PF_6_^−^ group can easily close to the Cu (I) and stabilize it more effectively than the eclipse form (Fig. 3). Therefore, in line with the experimental observation, the major formed isomeric complex would be the (*S,*a*S,S*) isomer^15,18,19,24^.

Natural Bond Orbital (NBO) analysis is a convenient method to simplify the analysis of intra and intermolecular interactions between filled and virtual orbitals in molecules^70,71^. Therefore, NBO calculation was used at the B3LYP/6-31G(*d*,*p*) level to determine stabilization energy (*E*^(2)^)^72,73^, and to specify partial charge on the selected atoms, as well. Then the highest values of *E*^(2)^, namely the strongest interactions, were selected to examine interactions in the Cu (I)-(*S,*a*S,S*)**-1a** and Cu (I)-(*S,*a*S,S*)**-1b** complexes. As expected, the stabilization energies, *E*^(2)^, have shown that the nitrogen atoms have a main role in coordination with the Cu (I). In the Cu (I)-(*S,*a*S,S*)**-1a** complex, the highest interaction energy between the donor orbital (the nitrogen lone pair) and the acceptor antibonding orbital (the lone pair star on the Cu (I) metal) is 55.39 kcal/mol. While, this energy in the case of Cu (I)-(*S,*a*S,S*)**-1b** complex is lower (44.62 kcal/mol). The stabilization energies for other types of interactions are listed in Table 3.

**Table 3:** *E*^(2)^ parameter on selected atoms of the Cu (I)-(*S,aS,S*)-complexes using NBO calculation.

Cu (I)-(*S,*a*S,S*)**-1b** complex			Cu (I)-(*S,*a*S,S*)**-1a** complex		
Donor	Acceptor	*E*^(2)^ (kcal/mol)	Donor	Acceptor	*E*^2^(kcal/mol)
LP (1) N 33	LP*(6) Cu 37	44.62	LP (1) N 33	LP*(6) Cu 37	55.39
LP (1) N 36	LP*(6) Cu 37	44.62	LP (1) N 36	LP*(6) Cu 37	55.39
CR (2) P 38	LP*(7) Cu 37	11.83	LP*(1) P 60	LP*(7) Cu 37	11.81
LP*(1) P 38	LP*(7) Cu 37	14.24	CR (2) P 60	LP*(7) Cu 37	10.54
LP*(2) P 38	LP*(6) Cu 37	13.60	LP*(2) P 60	LP*(6) Cu 37	14.92
LP (3) F 39	LP*(6) Cu 37	14.62	LP (3) F 61	LP*(6) Cu 37	12.70
LP (3) F 39	LP*(7) Cu 37	12.76	LP (3) F 61	LP*(7) Cu 37	11.00
LP (3) F 44	LP*(6) Cu 37	14.62	LP (3) F 66	LP*(6) Cu 37	12.70
LP (3) F 44	LP*(7) Cu 37	12.74	LP (3) F 66	LP*(7) Cu 37	11.00

**Figure 4:**
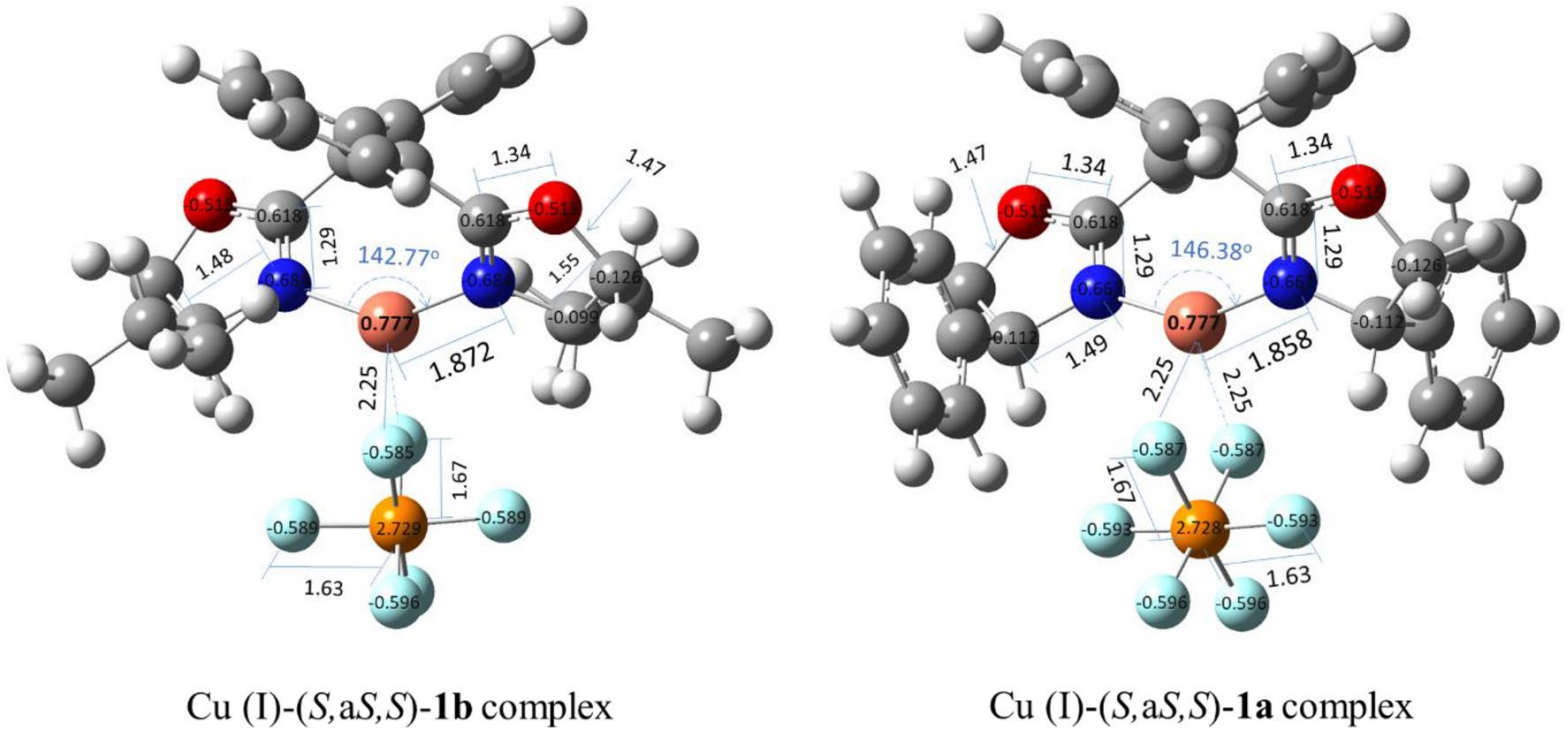
Bond lengths and partial charges on selected atoms in the Cu (I)-(*S*,a*S*,*S*)-**1a** and -**1b** complexes*.*

In addition, the comparison of the length bond between the nitrogen atom and the Cu (I) fragment of the two mentioned complexes have shown that the complex Cu (I)-(*S,*a*S,S*)**-1a** possess fewer length bond (1.4 pm). It seems that this slight difference caused a dramatic increase (10.77 kcal/mol) in *E*^(2)^ energy (Table 3 and Fig. 4).

**Figure 5:**
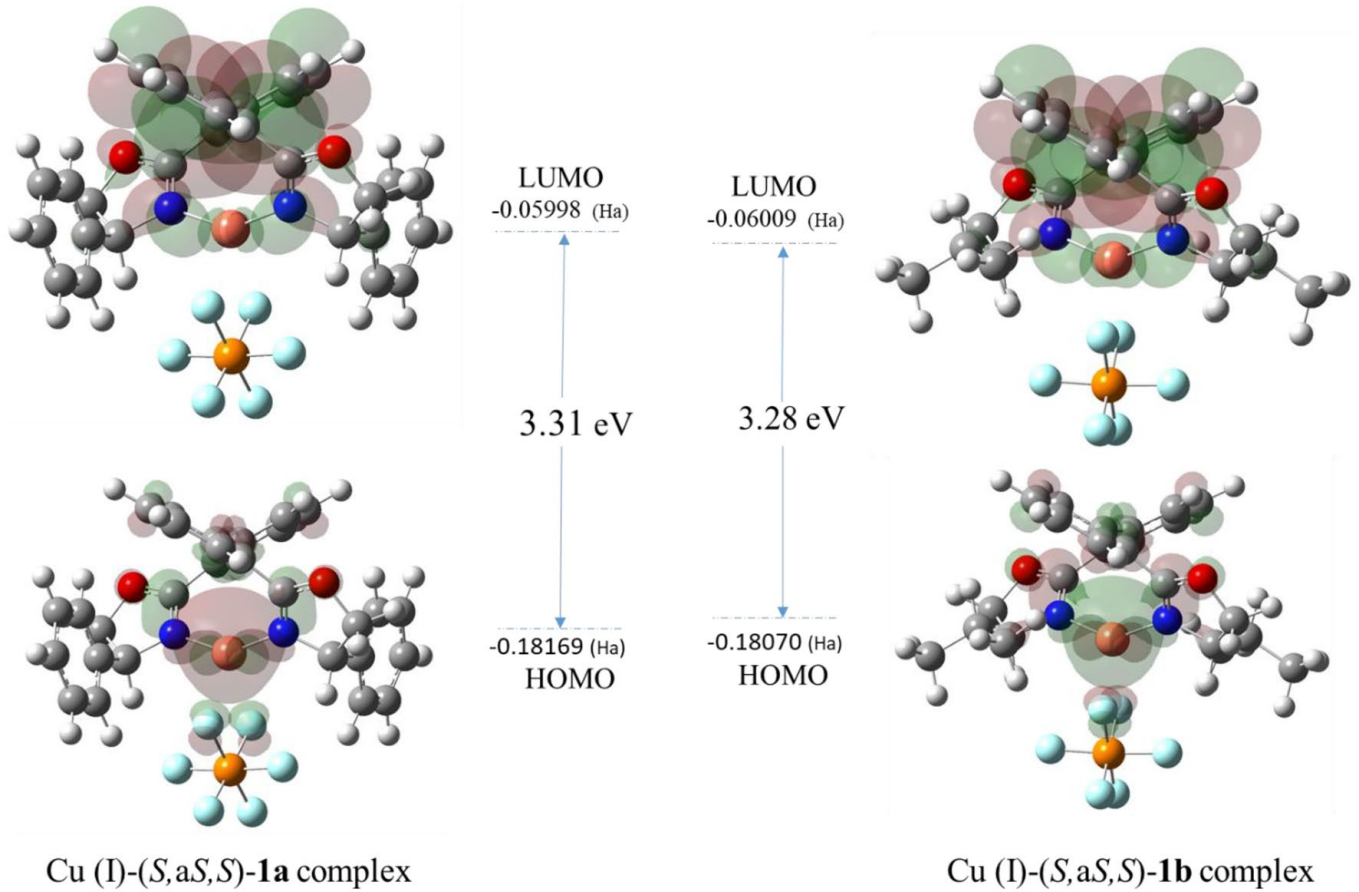
HOMO–LUMO energy gaps of the Cu (I)*-*(*S,aS,S*)-**1a** and Cu (I)*-*(*S,aS,S*)-**1b** complexes.

In the following, to compare the chemical activity of the complexes, the HOMO–LUMO energy gaps of the (*S,*a*S,S*) complexes were calculated^74,75^. In fact, the lower the HOMO–LUMO gap, the higher the chemical activity. The HOMO–LUMO gap values for the Cu (I)-(*S,*a*S,S*)-**1a**- and -**1b** complexes are 3.31 eV and 3.28 eV, respectively (Fig. 5). The Cu (I)-**1b** complex possesses a very slightly lower (30 meV) band-gap energy than the Cu (I)-**1a** complex. Although analyzing the HOMO–LUMO gaps indicates a very little higher chemical activity in favor of the Cu (I)-**1b** complex, it seems that the formation of enantiomerically enriched allylic esters is controlled by steric hindrance. In addition, the large electron density on the Cu (I) fragment reveals that this metal ion plays a significant role in the HOMO orbital.

**Figure 6:**
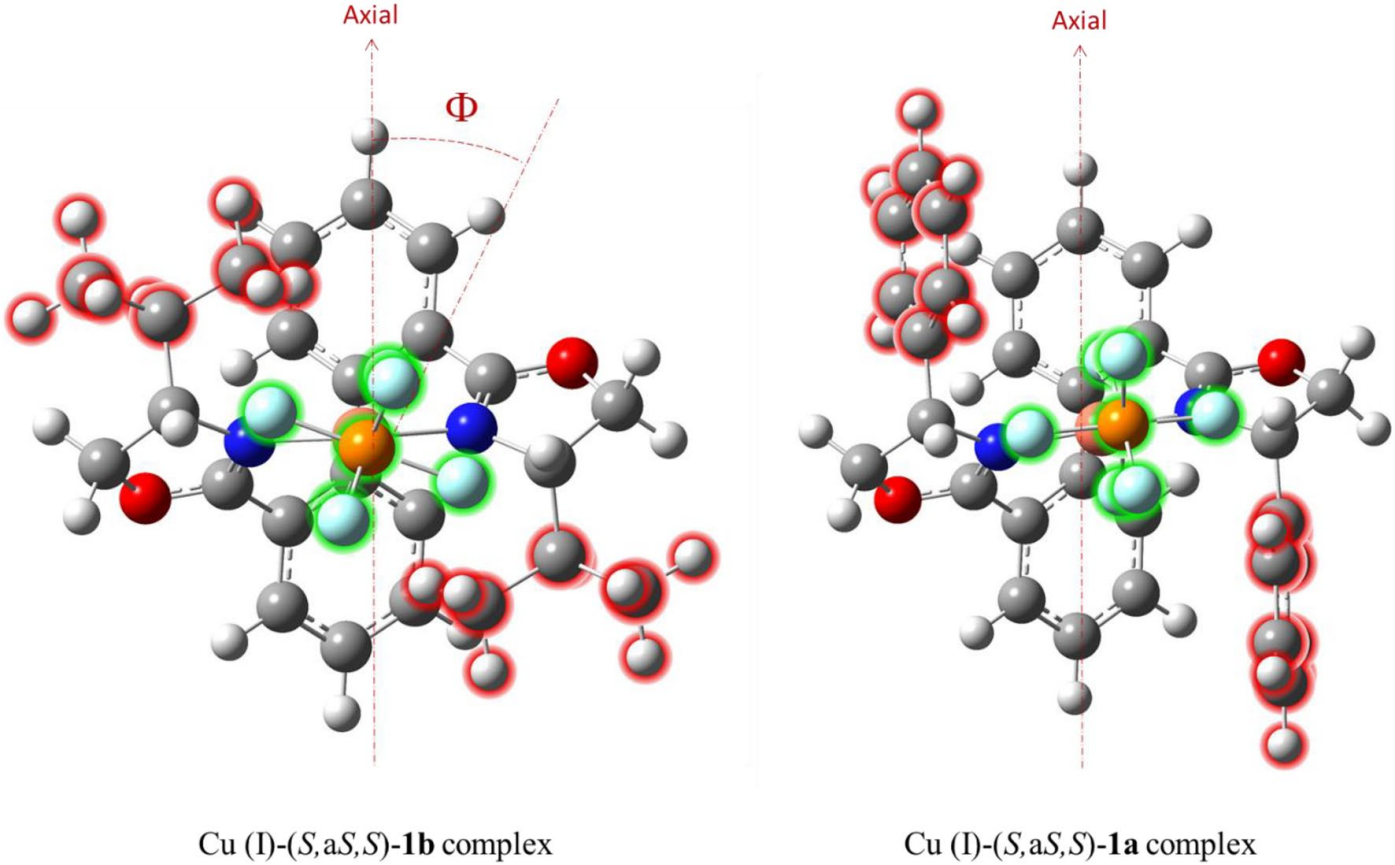
Steric repulsion caused by phenyl and *iso*propyl substitutions.

As it was explained, in the Cu (I)-(*S,*a*S,S*)**-1b** complex, the steric congestion caused by the *iso*propyl substitutes is more than the phenyl groups in the Cu (I)-(*S,*a*S,S*)**-1a** complex, and as a result, the N-Cu–N bond angle has to become a little smaller in the Cu (I)-(*S,*a*S,S*)**-1b** complex than the one in another complex. Although this help the steric repulsion arising from the *iso*propyl groups decrease to some extent, yet the PF_6_^−^ group has to approach to the Cu (I) atom with a slight tilt angle (Ф). In contrast, in the Cu (I)-(*S,*a*S,S*)**-1a** complex, there is no significant steric congestion; the PF_6_^−^ group can approach to the Cu (I) atom with a little tilt (Fig. 6). Therefore, it seems that the reactants, olefin and perester, can properly close to the Cu (I)-(*S,*a*S,S*)**-1a** complex and generate chiral allylic esters with higher enantioselectivities than the other complex.

**Figure 7:**
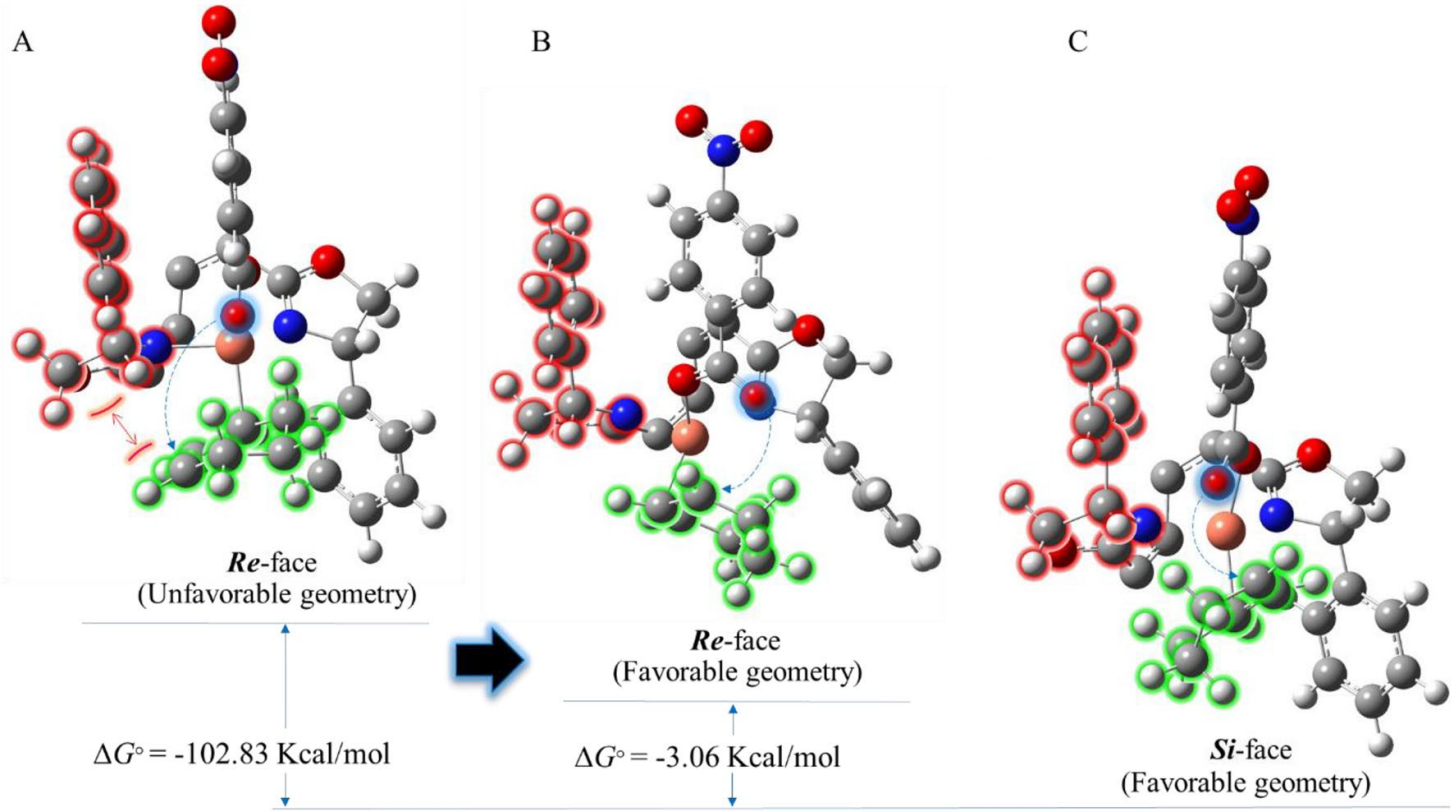
Steric hindrance and Gibbs free energies of the *Si*-face and *Re*-face of the key reaction intermediate containing cyclohexene reactant. To better visibility, some atoms of biphenyl backbone are not shown.

To investigate the enantioselectivity and yields of the allylic oxidation reaction, DFT calculations of the key reaction intermediate stage (the mechanism proposed in Scheme 3) were performed at the B3LYP-D3/6-31G (*d*,*p*) level of theory for cyclohexene and 1-hexene reactants and *para*-nitrobenzoate as the perester of the reaction in acetonitrile. To this end, our selected conformers not only must have minimum energy but also must follow a pericyclic rearrangement similar to the key intermediate stage. The computational results indicate that this intermediate is more stable thermodynamically when the oxygen atom attacks the cyclohexyl group from the *Si*-face side than the *Re*-face side by − 3.06 kcal/mol (Fig. 7). Based on the optimized geometry structures, in the *Si*-face intermediate the nucleophile (i.e., oxygen atom) can easily approach the double bond, on the contrary, in the *Re*-face intermediate the oxygen cannot simply get close to the double bond due to the existence of steric hindrance between the oxazoline ring (in red) of the catalyst and cyclohexyl group (in green) (Fig. 7A). Therefore, to reduce repulsive interactions, the cyclohexnyl group has to get away from the oxazoline moiety so that the oxygen atom can get close to the double bond (Fig. 7B). In this situation, although the *Re*-face intermediate can avoid undesirable interactions to some degree, it remains unstable thermodynamically compared to the *Si*-face intermediate (Fig. 7C).

**Figure 8:**
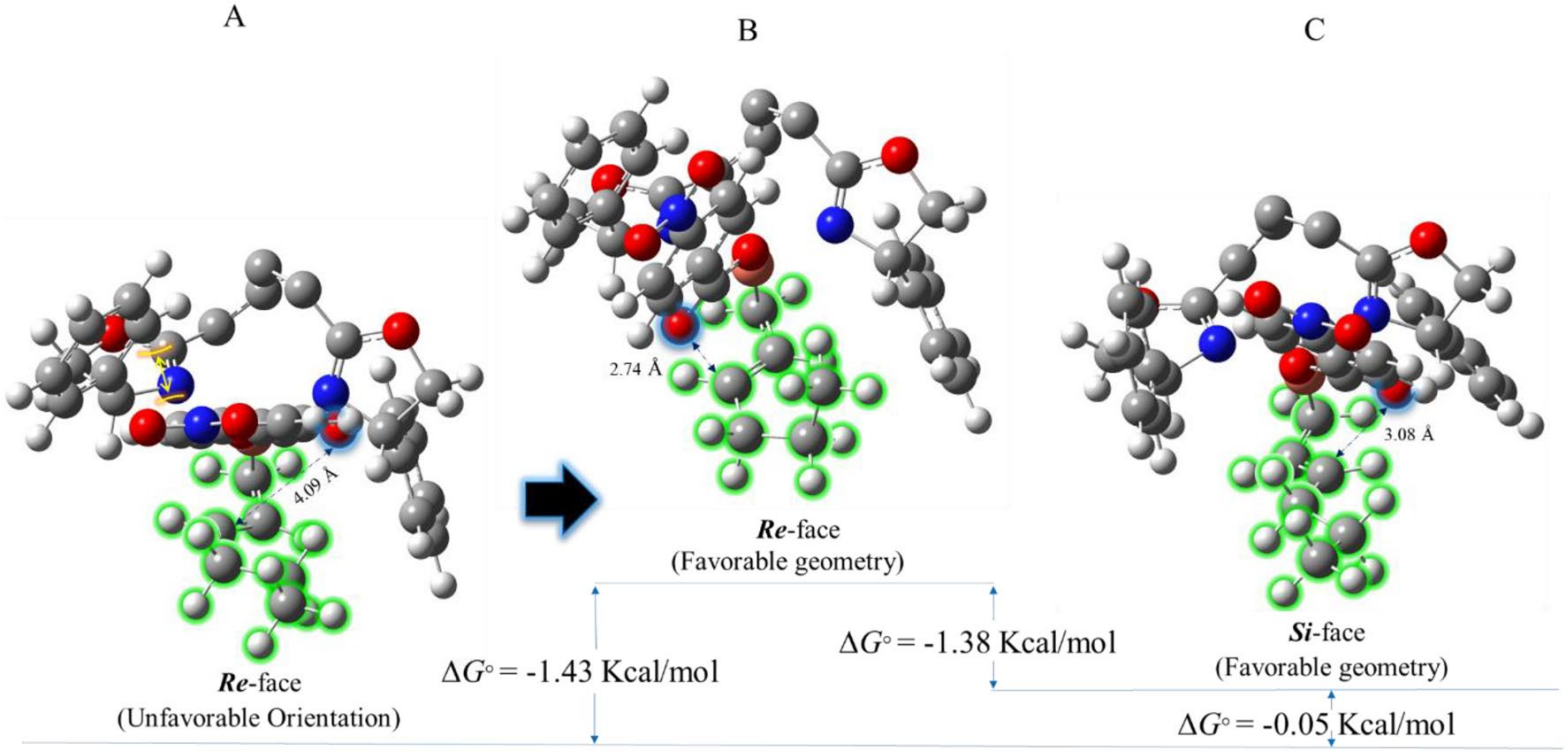
Geometries and Gibbs free energies of the *Si*-face and *Re*-face of the key reaction intermediate containing 1-hexene reactant. To better visibility, some atoms of biphenyl backbone are not shown.

On the other hand, since acyclic compounds are very flexible, they can find a variety of geometries; consequently, they can get rid of the steric congestion caused by the oxazoline ring well. Accordingly, for the intermediate bearing 1-hexenyl group, the steric congestion does not make a significant difference between energy levels of the *Si*-face and *Re*-face in the key reaction intermediate stage. As a result, the *ee* and yield of products would be very low compared to the cyclic compounds. Albeit conformational search indicates a slight preference (by − 0.05 kcal/mol) for the *Re*-face intermediate (Fig. 8A), in this conformer, the orientation of the nucleophile (i.e., oxygen atom) is opposite to the double bond; thereby, the *p*ara-nitro benzoate group must have a significant amount of rotation to perform the desired reaction. According to our initial condition for intermediates, having an appropriate orientation between the nucleophile and electrophile is essential for a pericyclic rearrangement. Therefore, the Gibbs free energy difference between *Si*-face and *Re*-face intermediates containing favorable orientation is − 1.38 kcal/mol in favor of the *Si*-face intermediate (Fig. 8B,C).

Most importantly, the difference in enantiomeric excesses and yields of the cyclic and acyclic products is due to the difference in steric congestion that these reactants encounter in the key reaction intermediate stage. In the case of the 1-hexene reactant, the 1-hexenyl group can avoid the steric congestion caused by the oxazoline ring, thus the difference in thermodynamic stability between *Re*- and *Si*-face conformers are very close to each other (1.38 kcal/mol). For that reason, enantiomeric excess and yields of its products would be lower compared to the rigid structure of the intermediate containing cyclohexene, in which related ∆*G*^o^ is − 3.08 kcal/mol. These results are in line with the experimental data in Table 2.

Another factor that is effective in the low yields of acyclic compounds is their non-symmetric geometries, leading to a diversity of intermediates via coordinating different carbon types to the copper catalyst. Hence, the nucleophile can attack a variety of carbons, produce a range of products, and as a result, reduce the yield and *ee* of the products.

## Conclusion

In summary, asymmetric copper catalyzed allylic C-H bond oxidation of cyclic and acyclic alkenes with a series of substituted *t*-butyl perbenzoates in the presence of (*S,*a*S,S*)-atropisomeric BOX ligands **1a** and **1b** (which can be easily obtained from a mixture of their *S*, *R* epimers, through the chelation-induced process) and porous inorganic additives was studied. It was revealed that at room temperature in CH_3_CN in a combination of ligand Cu(CH_3_CN)_4_PF_6_-**1a** and additives of PhNHNH_2_ and porous HZSM-5, peresters containing electron withdrawing groups at phenyl ring led to the corresponding chiral allylic esters with an excellent level of enantioselectivities and chemical yields in relatively short times. The DFT results of the Cu (I)-BOX complexes provided a deeper understanding of more thermodynamic stability of the (*S,*a*S,S*) complexes than their (*S*,a*R*,*S*) isomers. It can be inferred that due to the less sterically congestion of Cu (I)-(*S*, a*S*,* S*)-**1a** complex, the reactants are able to easily place in the appropriate position, leading to enantioenriched allylic esters with higher enantioselectivity compared to the Cu (I)-(*S*,a*S*,*S*)-**1b** complex. Finally, it turns out that enantiomeric excess of the cyclic products is controlled by steric hindrance arose from oxazoline moiety of the copper catalyst and incoming reactants in the key reaction intermediate stage.

## Experimental

### Typical procedure for the synthesis of bishydroxylamides 6a and 6b

In a 25 mL flame dried, 2-necked flask, under nitrogen, biphenyl dicarboxylic acid **3** (0.48 g, 2.0 mmol) was dissolved in 6 mL dichloromethane. Then, the reaction was cooled to 0 °C, and then oxalyl chloride (0.84 mL, 8 mmol) was added slowly followed by 3 drops of DMF. After stirring the mixture for 4 h at room temperature, evaporation of the solvent in *vaccuo* gave diacyl chloride as a light yellow solid (0.56 g, 99%). The obtained diacyl chloride was dissolved in 6 mL CH_2_Cl_2,_ and at 0 °C, slowly added to a solution of (*S*)-phenyl glycinol **5a** (0.6 g, 2.4 mmol) and Et_3_N (0.67 mL) in 6 mL CH_2_Cl_2_ during 30 min. The mixture was allowed to warm to room temperature and stirred overnight. Monitoring the reaction by TLC (90:10 EtOAc/*n*-hexane) showed two compounds (*S*,a*S*,*S*)- and (*S*,a*R*,*S*)-**6a**. After the reaction was completed, it was washed with brine (10 mL) and the organic layer was separated, and then the aqueous layer was extracted with EtOAc (3 × 15 mL). The combined organic layer was dried over MgSO_4_ and concentrated in *vaccuo*. Purification of the residue by silica gel column chromatography (eluent: EtOAc/*n*-hexane; 80–100: 20–0) gave a white solid **6a** in 95% yield. Compound **6b** was prepared according to the same procedure in 98% yield^15,18^.

### Typical procedure for the synthesis of ligands 1a and 1b

In order to cyclization of **6a**, under nitrogen atmosphere*,* in an oven-dried round-bottom flask bishydroxylamide **6a** (1 mmol, 0.48 g, 1 equiv) was dissolved in CH_2_Cl_2_ and 4-(dimethylamino) pyridine (0.01 g, 0.1 mmol, 0.1 equiv) was added. After cooling to 0 °C, Et_3_N (0.6, 4.4 mmol, 4.4 equiv), and a solution of *p*-TsCl (0.38 g, 2 mmol, 2 equiv) in 2 mL of dichloromethane were added. The mixture was stirred at ambient temperature for 18 h and then washed with saturated aqueous NH_4_Cl (10 mL). The aqueous layer was extracted with CH_2_Cl_2_ (3 × 10 mL), and the combined organic layers washed with 10 mL saturated NaHCO_3 (aq)_, dried with Na_2_SO_4_ and evaporated under *vaccuo*. Purification of the resulting light yellow oil by column chromatography (*n*-hexane/EtOAc; 90:10); resulted in pure light yellow **1a** (95%); (61 (*S*,a*S*,*S*): 39 (*S*,a*R*,*S*)). Ligand **1b** was synthesized by the similar protocol in 85% yield; (80 (*S*,a*S*,*S*): 20 (*S*,a*R*,*S*) ^15,18^.

### General procedure for the synthesis of the Cu (I)-1-complex

Under nitrogen atmosphere, 1 equiv of Cu(CH_3_CN)_4_PF_6_ (0.018 mmol, 6.6 mg) was added to ligand **1** (0.02 mmol) dissolved in 1 mL of chloroform-*d* and stirred at room temperature for 3 h. Monitoring the reaction by TLC revealed a single new spot^14,15^.

### Typical procedure for asymmetric Kharasch–Sosnovsky reaction

Under a nitrogen atmosphere, at room temperature, a 10 mL flame dried schlenk flask was charged with dried acetonitrile (2 mL), Cu(CH_3_CN)_4_PF_6_ (10 mg, 0.027 mmol) and chiral ligand **1a** (14 mg, 0.032 mmol) and stirred for 2 h. Then, phenyl hydrazine (5 *μ*L, 0.05 mmol) and HZSM-5 (5 mg) were added. After a few minutes, cyclohexene (2.5 mmol, 0.25 mL) was added slowly, and the reaction mixture was cooled to 0 °C, and *tert*-butyl-*p*-nitrobenzoperoxoate **7a**^15,18,50–52^ (0.85 mmol, 0.203 g) was added portionwise, and then stirred at 0 °C until complete disappearance of **7a** (TLC). After that, 5 mL 10% NH_4_OH was added to the mixture and extracted with EtOAc (3 × 5 mL). A yellow residue was obtained after evaporation of the solvent. Column chromatography of the obtained residue on silica gel afforded (*S*)-2-cyclohexenyl-*p*-nitrobenzoate as a white solid (98%, 93% *ee*). The bisoxazoline ligand was also recovered in 92% yield^15,18,50–52,76–80^.

### Computational method

In this study, the phenyl and *iso*propyl group substitutions were selected to examine the effect of aryl and alkyl groups on the isomeric complexes. First structures were drawn using Spartan software^81^ and gauss view 6^82^, and then Gaussian 9^83^ and Gaussian 16 were employed for DFT calculations. Both frequency and optimization calculations were carried out with the B3LYP method at 6-31G (*d*,*p*) basis set level and CPCM as the method for chloroform solvent. The basis set was selected based on two factors: computational cost and an excellent agreement between computational and experimental results. In addition, long-range van der Waals interactions are taken into account using the Grimm’s D3 dispersion correction for complexes (i.e. B3LYP-D3/6-31G (*d*,*p*) level of theory) to make the DFT energies of complexes more accurate. Different conformers of complexes with *C*_1_ and *C*_2_ symmetries were designed and optimized at the 6-31G (*d*,*p*) basis set level. The selection criterion for determining the stable conformers was the Gibbs free energy of the systems and not the symmetry of the molecules. All frequencies were done without imaginary frequency. The absence of imaginary frequencies in the computational outputs indicates that all optimized structures are at their minimum energy in the potential energy surfaces diagram. Moreover, Gibbs free energies were calculated to determine the thermodynamic stability of the complexes. In the case of intermediates containing 1-hexenyl groups, different conformers, including *cis* and *trans* forms of the 2-hexenyl group, were investigated to find global minimums. In addition, to determine the relative amounts of each isomer at equilibrium, equilibrium constant (*K*eq) at the 298.15 K, one atmosphere pressure and 1.98 × 10^–3^ kcal/(K mole) as gas constant, were calculated^84^.

## Supplementary material

Supplementary material (included materials and characterization methods, the typical procedure for the synthesis of **5** and **7–14**, and Figure S46 and geometry optimized coordinates of compounds) associated with this article can be found in the online version.

## Supplementary Information

Supplementary Information.

## Data Availability

The generated and analyzed data during the current study is supplied in this manuscript and supplementary material.
