# Supplementary material for: Preparation and DFT studies of chiral Cu (I)-complexes of biphenyl bisoxazolines and their application in enantioselective Kharasch–Sosnovsky reaction
Source: Sci Rep. 2022 Sep 3;12:15038. doi: 10.1038/s41598-022-18922-1 (PMC9440904; doi:10.1038/s41598-022-18922-1)
Supplement: Supplementary file 1 — Supplementary Information. [file 41598_2022_18922_MOESM1_ESM.pdf]

Supplementary material

Preparation and DFT studies of chiral Cu (I)-complexes of biphenyl bisoxazolines and their application in enantioselective Kharasch-Sosnovsky reaction

Saadi Samadi*, Hamid Arvinnezhad, Sirwan Mansoori & Hadi Parsa

Laboratory of Asymmetric Synthesis, Department of Chemistry, Faculty of Science, University of Kurdistan, Sanandaj 66177-15175, Iran. Phone: (+9887) 33624133; Email: s.samadi@uok.ac.ir.

Page	List of contents	Page	List of contents
S1	Title, author's name, address	S31	Figure S25: ^{13}CNMR of **8c**
S2-S6	Experimental section	S32	Figure S26: ^{1}HNMR of **8d**
S7	Figure S1: ^{1}HNMR of **5a**	S33	Figure S27: ^{13}CNMR of **8d**
S8	Figure S2: ^{13}CNMR of **5a**	S34	Figure S28: ^{1}HNMR of **8g**
S9	Figure S3: ^{1}HNMR of **5b**	S35	Figure S29: ^{13}CNMR of **8g**
S10	Figure S4: ^{13}CNMR of **5b**	S36	Figure S30: ^{1}HNMR of **9a**
S11	Figure S5: ^{1}HNMR of **6a**	S37	Figure S31: ^{13}CNMR of **9a**
S12	Figure S6: ^{1}HNMR of **6b**	S38	Figure S32: ^{1}H NMR of **9d**
S13	Figure S7: ^{13}CNMR of **6b**	S39	Figure S33: ^{13}C NMR of **9d**
S14	Figure S8: ^{1}HNMR of **1a**	S40	Figure S34: ^{1}H NMR of **10a**
S15	Figure S9: ^{13}CNMR of **1a**	S41	Figure S35: ^{13}C NMR of **10a**
S16	Figure S10: ^{1}HNMR of **1b**	S42	Figure S36: ^{1}H NMR of **10b**
S17	Figure S11: ^{13}CNMR of **1b**	S43	Figure S37: ^{13}C NMR of **10b**
S18	Figure S12: ^{1}HNMR of **1a**-Cu	S44	Figure S38: ^{1}H NMR of **10d**
S19	Figure S13: ^{13}CNMR of **1a**-Cu	S45	Figure S39: ^{13}C NMR of **10d**
S20	Figure S14: ^{1}HNMR of **1b**-Cu	S46	Figure S40: ^{1}H NMR of **11a**
S21	Figure S15: ^{13}CNMR of **1b**-Cu	S47	Figure S41: ^{13}C NMR of **11a**
S22	Figure S16: ^{1}HNMR of **7a**	S48	Figure S42: ^{1}H NMR of **11b**
S23	Figure S17: ^{13}CNMR of **7a**	S49	Figure S43: ^{13}C NMR of **11b**
S24	Figure S18: ^{1}HNMR of **7d**	S50	Figure S44: ^{1}H NMR of **11d**
S25	Figure S19: ^{13}CNMR of **7d**	S51	Figure S45: ^{13}C NMR of **11d**
S26	Figure S20: ^{1}HNMR of **8a**	S52	Computational method
S27	Figure S21: ^{13}CNMR of **8a**	S53	Figure S46: Gibbs free energy and Equilibrium
S28	Figure S22: ^{1}H NMR of **8b**	S54-S63	Geometry optimized coordinates of the compounds
S29	Figure S23: ^{13}C NMR of **8b**	S64-S76	Calculations of the key reaction intermediate
S30	Figure S24: ^{1}H NMR of **8c**	S77-S78	References

Experimental:

Materials and characterization methods

Melting points were measured by using an Electrothermal 9100 apparatus and uncorrected. NMR spectra were recorded in $CDCl_3$, and DMSO-d_6 using TMS ($\delta = 0.0$ ppm) as internal standard on a BRUKER DRX-300 AVANCE spectrometer at 300.13 for ^{1}H and 75.47 MHz and 100 MHz for ^{13}C. FT-IR spectra were determined on a Bomen FT-IR-MB-series instrument. Optical rotations were measured at the sodium D line on a Perkin–Elmer 341 polarimeter. Enantiomeric excess (*ee*) of the resulting chiral allylic esters were determined by HPLC on Chiralpak AD and/or Chiralcel OD-H columns and/or Nucleocel Alpha S columns. All the reactions were accomplished under a dry atmosphere, oxygen-free nitrogen in flame dried glassware. All starting materials and reagents were purchased from Aldrich and Merck. Cycloolefins were purified by distillation from CaH_2 prior to use. All solvents were of reagent grade and were dried and distilled immediately before use as follows: acetonitrile, acetone and chloroform from P_2O_5; methylene chloride and *n*-hexane from CaH_2; toluene from sodium/benzophenone. Column chromatography was performed on silica gel 60 (230–400 mesh) eluting with ethyl acetate/*n*-hexane. Reactions were monitored by TLC on silica gel 60 F_{256} plates with visualization by UV.

Typical procedure for the synthesis of chiral amino alcohols 5a and 5b:

Sodium borohydride (16.0 mmol, 6.0 g) and 8 mL of dried tetrahydrofuran were added to an oven-dried 3-neck 100 mL round-bottom flask equipped with reflux condenser. After 15 minutes, (*S*)-phenylglycine **4a** (6.6 mmol, 0.99 g) was added in one portion to the stirring solution. Then the resulting mixture was cooled to 0 °C, and I_2 (6.6 mmol, 1.67 g) dissolved in 4 mL of THF was added dropwise over 30 minutes. After the brownish color of the solution was faded away, the mixture was warmed to room temperature and then the white cloudy solution refluxed for 48 hours. After cooling to room temperature, 4 mL of CH_3OH was added dropwise, with fast stirring. The obtained solution was concentrated in *vaccuo*, and the residue dissolved in 4 mL of KOH (20%) and then stirred for 4 hours at room temperature. The resulting solution was extracted with CH_2Cl_2 (3 x 8 mL), the organic layer washed with brine, and then the aqueous layer back extracted with CH_2Cl_2 (8 mL). The combined organic layers were dried over anhydrous $MgSO_4$ and concentrated. Phenyglycinol **5a** was obtained as a white solid in 96% yield. (*S*)-valinol **5b** was also prepared from (*S*)-valine **4b** in the similar procedure with 98% yield [1-7].

Typical procedure for the synthesis of bishydroxylamides 6a and 6b:

In a 25 mL flame dried, 2-necked flask, under nitrogen, biphenyl dicarboxylic acid **3** (0.48 g, 2.0 mmol) was dissolved in 6 mL dichloromethane. Then, the reaction was cooled to 0 °C, and then oxalyl chloride (0.84 mL, 8 mmol) was added slowly followed by 3 drops of DMF. After stirring the mixture for 4 h at room temperature, evaporation of the solvent in *vaccuo* gave diacyl chloride as a light yellow solid (0.56 g, 99%). The obtained diacyl chloride was dissolved in 6 mL CH_2Cl_2, and at 0 °C, slowly added to a solution of (*S*)-phenyl glycinol **5a** (0.6 g, 2.4 mmol) and Et_3N (0.67 mL) in 6 mL CH_2Cl_2 during 30 min. The mixture was allowed to warm to room temperature and stirred overnight. Monitoring the reaction by TLC (90:10 EtOAc/*n*-hexane) showed two compounds (*S*,a*S*,*S*)- and (*S*,a*R*,*S*)-**6a**. After the reaction was completed, it was washed with brine (10 mL) and the organic layer was separated, and then the aqueous layer was extracted with EtOAc (3 ×15 mL). The combined organic layer was dried over $MgSO_4$ and concentrated in *vaccuo*. Purification of the residue by silica gel column chromatography (eluent: EtOAc/*n*-hexane; 80–100: 20–0) gave a white solid **6a** in 95% yield. Compound **6b** was prepared according to the same procedure in 98% yield[5,7].

Typical procedure for the synthesis of ligands 1a and 1b:

In order to cyclization of **6a**, under nitrogen atmosphere, in an oven-dried round-bottom flask bishydroxylamide **6a** (1 mmol, 0.48 g, 1 equiv) was dissolved in CH_2Cl_2 and 4-(dimethylamino) pyridine (0.01 g, 0.1 mmol, 0.1 equiv) was added. After cooling to 0 °C, Et_3N (0.6, 4.4 mmol, 4.4 equiv), and a solution of *p*-TsCl (0.38 g, 2 mmol, 2 equiv) in 2 mL of dichloromethane were added. The mixture was stirred at ambient temperature for 18 h and then washed with saturated aqueous NH_4Cl (10 mL). The aqueous layer was extracted with CH_2Cl_2 (3×10 mL), and the combined organic layers washed with 10 mL saturated $NaHCO_{3\,(aq)}$, dried with Na_2SO_4 and evaporated under *vaccuo*. Purification of the resulting light yellow oil by column chromatography (*n*-hexane/EtOAc; 90:10); resulted in pure light yellow **1a** (95%); (61 (*S*,a*S*,*S*): 39 (*S*,a*R*,*S*)). Ligand **1b** was synthesized by the similar protocol in 85% yield; (80 (*S*,a*S*,*S*): 20 (*S*,a*R*,*S*)[5,7].

General procedure for the synthesis of the Cu (I)-1-complex

Under nitrogen atmosphere, 1 equiv of $Cu(CH_3CN)_4PF_6$ (0.018 mmol, 6.6 mg) was added to ligand **1** (0.02 mmol) dissolved in 1 mL of chloroform-*d* and stirred at room temperature for 3 h. Monitoring the reaction by TLC revealed a single new spot[5,8].

Typical procedure for asymmetric Kharasch-Sosnovsky reaction:

Under a nitrogen atmosphere, at room temperature, a 10 mL flame dried schlenk flask was charged with dried acetonitrile (2 mL), $Cu(CH_3CN)_4PF_6$ (10 mg, 0.027 mmol) and chiral ligand **1a** (14 mg, 0.032 mmol) and stirred for 2 hours. Then, phenyl hydrazine (5 μL, 0.05 mmol) and HZSM-5 (5 mg) were added. After a few minutes, cyclohexene (2.5 mmol, 0.25 mL) was added slowly, and the reaction mixture was cooled to 0 °C, and *tert*-butyl-*p*-nitrobenzoperoxoate **7a**[3-7] (0.85 mmol, 0.203 g) was added portionwise, and then stirred at 0 °C until complete disappearance of **7a** (TLC). After that, 5 mL 10% NH_4OH was added to the mixture and extracted with EtOAc (3×5 mL). A yellow residue was obtained after evaporation of the solvent. Column chromatography of the obtained residue on silica gel afforded (*S*)-2-cyclohexenyl-*p*-nitrobenzoate as a white solid (98%, 93% *ee*). The bisoxazoline ligand was also recovered in 92% yield[3-7, 9-13].

(S)-Cyclohex-2-en-1-yl 4-nitrobenzoate **(8a)** [3,4,6,15-17]: $[\alpha]^{20}_D$ = –135.0° (*c* 1.0, $CHCl_3$); The optical purity was determined by HPLC with Nucleocel Alpha S column; eluent: *n*-hexane/isopropyl alcohol = 99.5/0.5; Flow rate: 0.6 mL/min; t_R = 27.7 min (*R*), 30.2 min (*S*)) (Maximum *ee* = 93%).

(S) Cyclohex-2-en-1-yl 4-iodobenzoate **(8b)** [3,4,6]: $[\alpha]^{20}_D$ = –86.8° (*c* 1.0, $CHCl_3$); The optical purity was determined by HPLC with Chiralpak AD column; eluent: *n*-hexane/isopropyl alcohol = 99.6/0.4; Flow rate: 0.6 mL/min; t_R = 25.0 min, 26.3 min (Maximum *ee* = 90%).

(S)- Cyclohex-2-en-1-yl 4-chlorobenzoate **(8c)** [3,4, 6,17]: $[\alpha]^{20}_D$ = –146.5° (*c* 1.0, $CHCl_3$); The optical purity was determined by HPLC with Chiralpak AD column; eluent: *n*-hexane/isopropyl alcohol = 99.6/0.4; Flow rate: 0.6 mL/min; t_R = 21.2 min (*R*), 23.5 min (*S*)) (Maximum *ee* = 87%).

(S)-Cyclohex-2-en-1-yl 2-iodobenzoate **(8d)** [3,4,6,13-15]: $[\alpha]^{20}_D$ = –158.2° (*c* 1.0, $CHCl_3$); The optical purity was determined by HPLC with Chiralpak AD column; eluent: *n*-hexane/isopropyl alcohol = 99.6/0.4; Flow rate: 0.5 mL/min; t_R = 23.5 min (*R*), 26.2 min (*S*)) (Maximum *ee* = 90%).

***(S)-Cyclohex-2-en-1-yl 2-chlorobenzoate* (8e)** [15]: $[\alpha]^{20}_{D}$ = –115.6° (*c* 1.0, $CHCl_3$); The optical purity was determined by HPLC with Chiralpak AD column; eluent: *n*-hexane/isopropyl alcohol = 99.6/0.4; Flow rate: 0.6 mL/min; t_R = 19.5 min (*R*), 22.0 min (*S*)) (Maximum *ee* = 80%).

***(S)-Cyclohex-2-en-1-yl benzoate* (8g)** [3,4,6]: $[\alpha]^{20}_{D}$ = –78.5° (c 1.0, $CHCl_3$).; The optical purity was determined by HPLC with Chiralcel OD-H column; eluent: *n*-hexane/isopropyl alcohol = 99.7/0.3; Flow rate: 0.5 mL/min; t_R = 18.6 min (*R*), 19.8 min (*S*)) (Maximum *ee* = 70%).

***(S) Cyclohex-2-en-1-yl 4-methoxybenzoate* (8i)** [5,15]: $[\alpha]^{20}_{D}$ = –62.0° (*c* 1.0, $CHCl_3$); The optical purity was determined by HPLC with Chiralpak AD column; eluent: *n*-hexane/isopropyl alcohol = 99.6/0.4; Flow rate: 0.6 mL/min; t_R = 22.7 min, 24.2 min (Maximum *ee* = 58%).

***(S)-Cyclopent-2-en-1-yl 4-nitrobenzoate* (9a)** [3,4,6,16,17]: $[\alpha]^{20}_{D}$ = –162.4° (*c* 1.0, $CHCl_3$); The optical purity was determined by HPLC with Nucleocel Alpha S column; eluent: *n*-hexane/isopropyl alcohol = 99.6/0.4; Flow rate: 0.4 mL/min; t_R = 36.2 min (*R*), 37.5 min (*S*)) (Maximum *ee* = 88%).

***(S)-Cyclopent-2-en-1-yl 2-iodobenzoate* (9d)** [3,4,6,13-15]: $[\alpha]^{20}_{D}$ = –89.0° (*c* 1.0, $CHCl_3$); The optical purity was determined by HPLC with Chiralpak AD column; eluent: *n*-hexane/isopropyl alcohol = 99.6/0.4; Flow rate: 0.6 mL/min; t_R = 21.1 min (*R*), 22.7 min (*S*)) (Maximum *ee* = 81%).

***(S)-Cyclooct-2-en-1-yl 4-nitrobenzoate* (10a)** [3,4,6]: $[\alpha]^{20}_{D}$ = +44.7° (*c* 1.0, $CHCl_3$); Optical purity was determined by HPLC with Chiralcel OD-H column; eluent: *n*-hexane/isopropyl alcohol = 99.6/0.4; Flow rate: 0.4 mL/min; t_R = 20.0 min (*R*), 22.7 min (*S*)) (Maximum *ee* = 86%).

***(S)-Cyclooct-2-en-1-yl 4-iodobenzoate* (10b)** [3,4,6]: $[\alpha]^{20}_{D}$ = +31.0° (*c* 1.0, $CHCl_3$); Optical purity was determined by HPLC with Chiralcel OD-H column; eluent: *n*-hexane/isopropyl alcohol = 99.5/0.5; Flow rate: 0.4 mL/min; t_R = 17.0 min, 20.5 min (Maximum *ee* = 75%).

***(S)-Cyclooct-2-en-1-yl 2-iodobenzoate* (10d)** [3,13,15]: $[\alpha]^{20}_{D}$ = +43.8° (*c* 1.0, $CHCl_3$); Optical purity was determined by HPLC with Chiralpak AD column; eluent: *n*-hexane/isopropyl alcohol = 99.6/0.4; Flow rate: 0.6 mL/min; t_R = 16.5 min (*R*), 19.1 min (*S*)) (Maximum *ee* = 69%).

***(S)-Cycloocta-2,6-dien-1-yl 4-nitrobenzoate* (11a)** [3,4,6,15-18]: $[\alpha]^{20}_{D}$= +25.1° (*c* 1.0, $CHCl_3$); Optical purity was determined by HPLC with Chiralcel OD-H column column; eluent: *n*-hexane/isopropyl alcohol = 99.3/0.7; Flow rate: 0.4 mL/min; t_R = 32.2 min (*S*), 34.0 min (*R*)) (Maximum *ee* = 98%).

***(S)-Cycloocta-2,6-dien-1-yl 4-iodobenzoate* (11b)** [3,4,6]: $[\alpha]^{20}_{D}$= +20.8° (*c* 1.0, $CHCl_3$); Optical purity was determined by HPLC with Chiralcel OD-H column column; eluent: *n*-hexane/isopropyl alcohol = 99.3/0.7; Flow rate: 0.4 mL/min; t_R = 27.5 min, 31.2 min (Maximum *ee* = 95%).

***(S)-Cycloocta-2,6-dien-1-yl 2-iodobenzoate* (11d)** [3,13,15]: $[\alpha]^{20}_{D}$= +33.7° (c 1.0, $CHCl_3$); Optical purity was determined by HPLC with Chiralcel OD-H column; eluent: *n*-hexane/isopropyl alcohol = 99.6/0.4; Flow rate: 0.5 mL/min; t_R = 29.5 min (*S*), 32.6 min (*R*)) (Maximum *ee* = 92%).

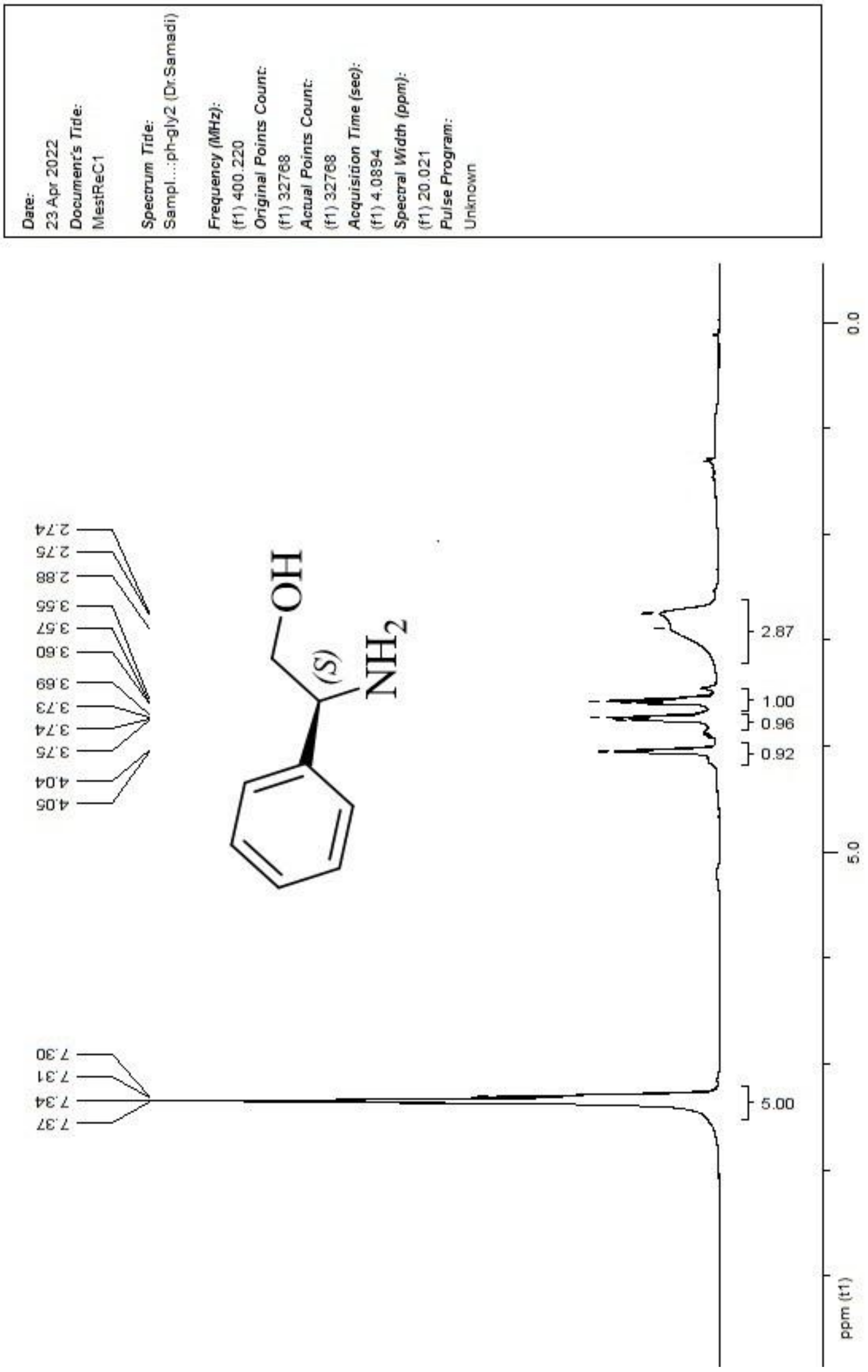

Figure S1: ^{1}H NMR of **5a**

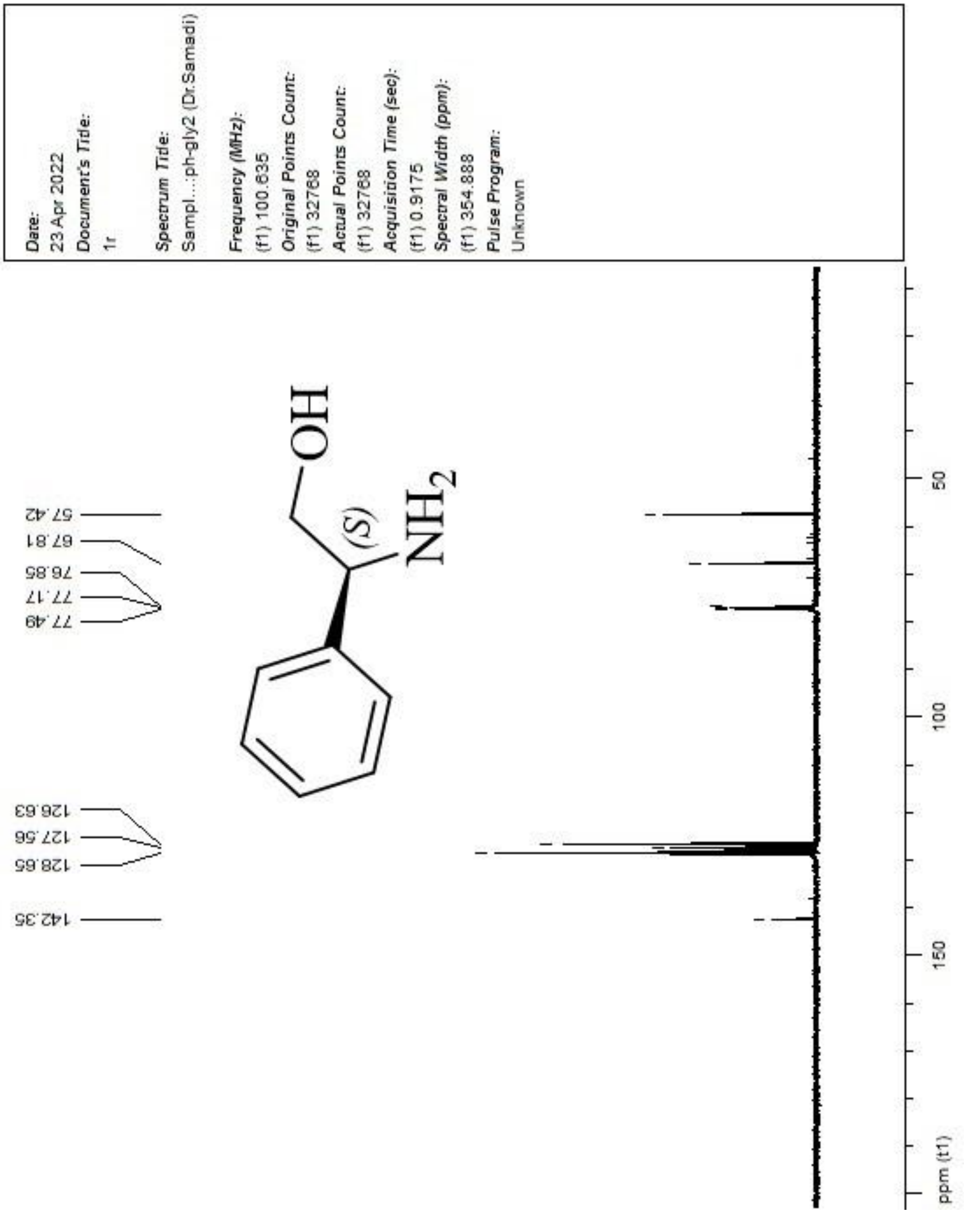

Figure S2: ^{13}CNMR of **5a**

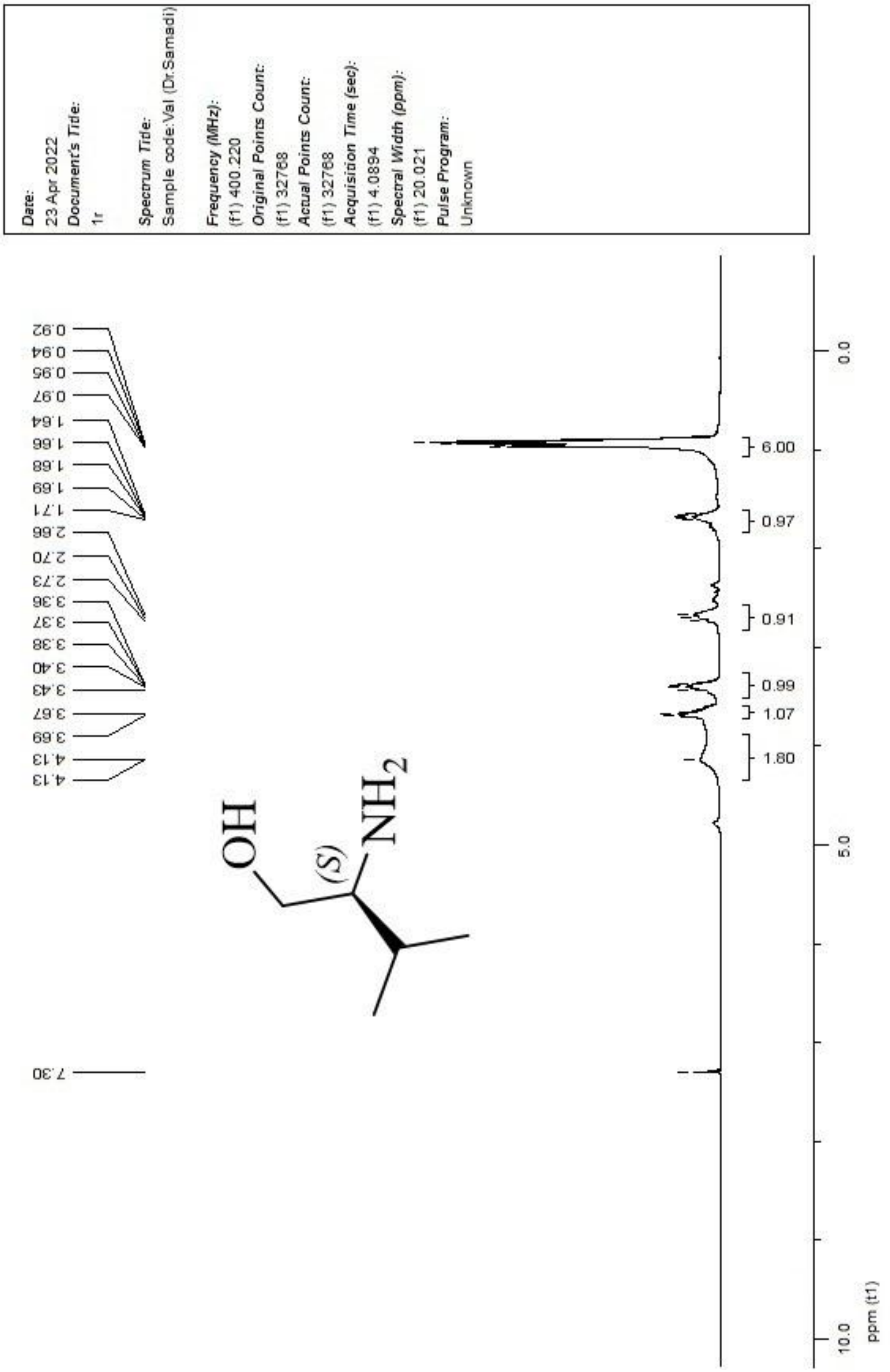

Figure S3: ^{1}H NMR of **5b**

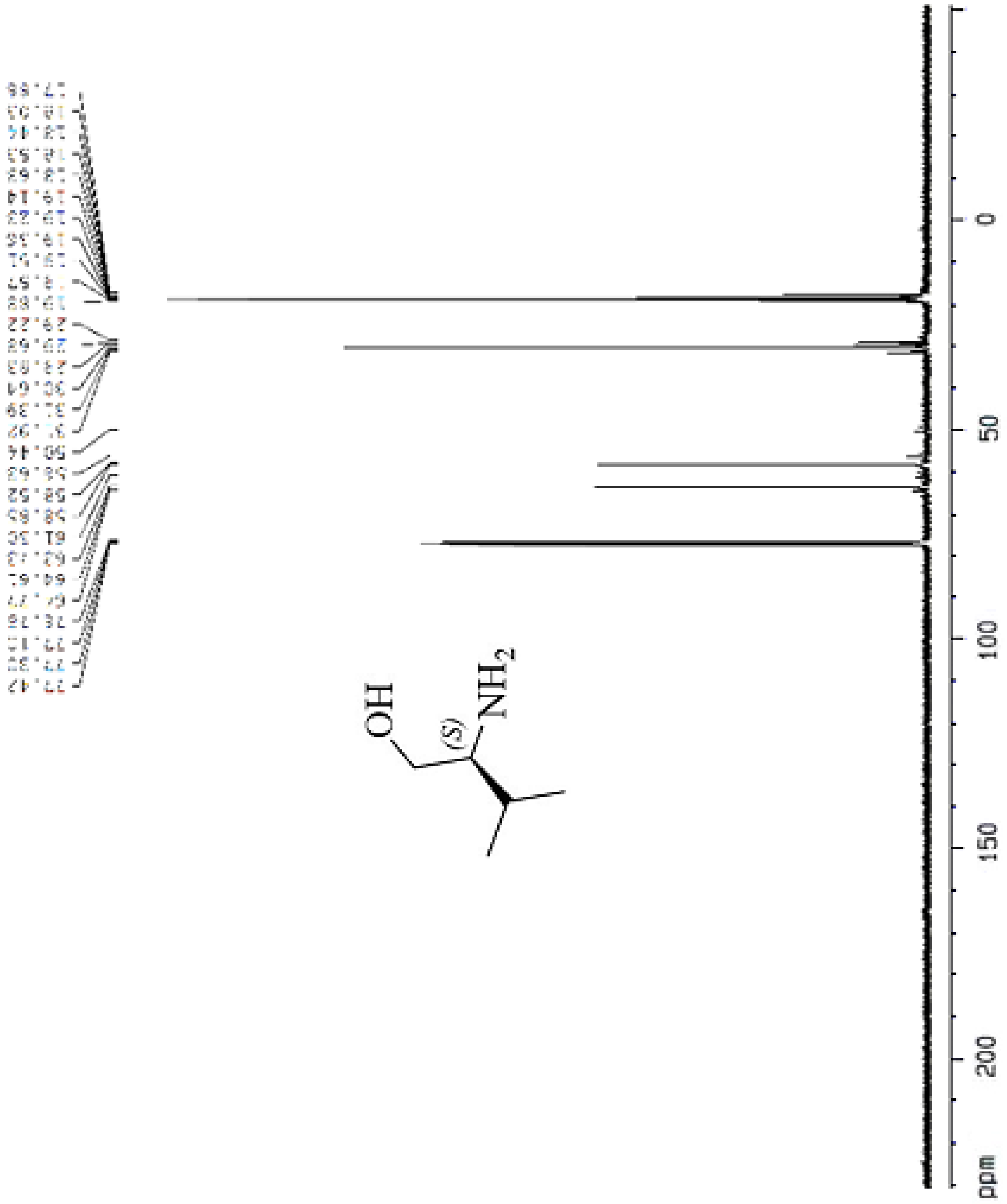

Figure S4: ^{13}CNMR of **5b**

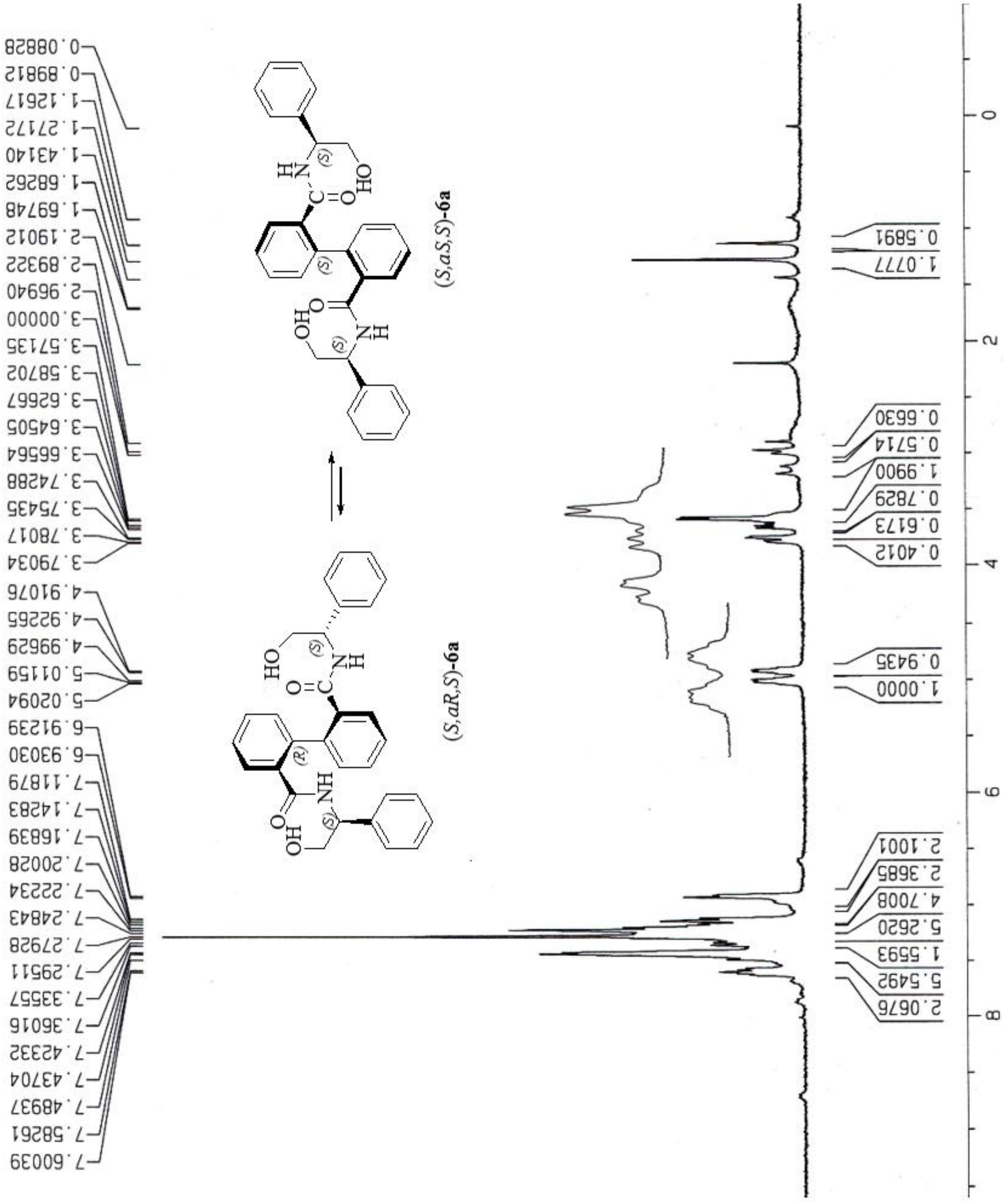

Figure S5: 1HNMR of **6a**

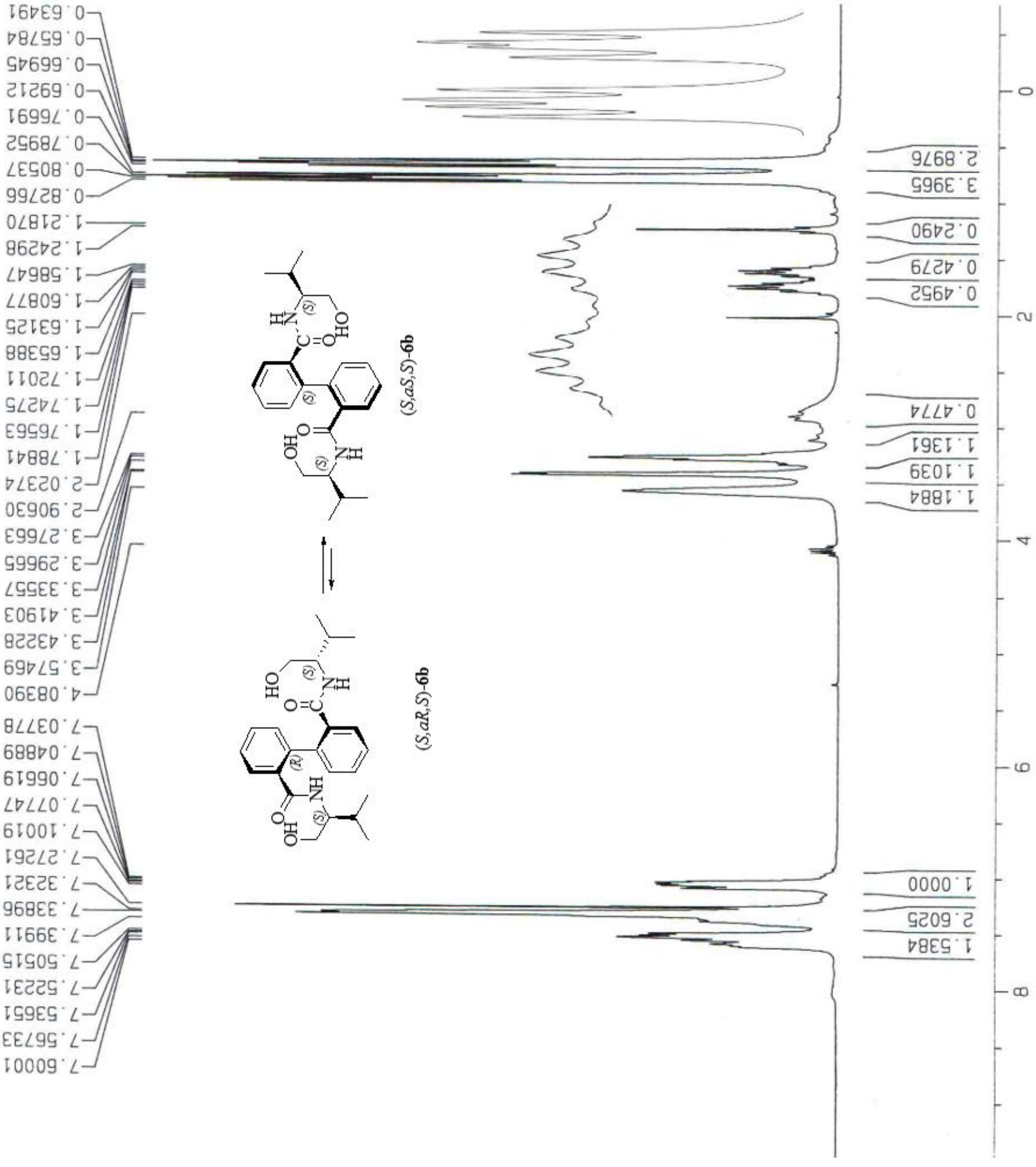

Figure S6: ^{1}HNMR of **6b**

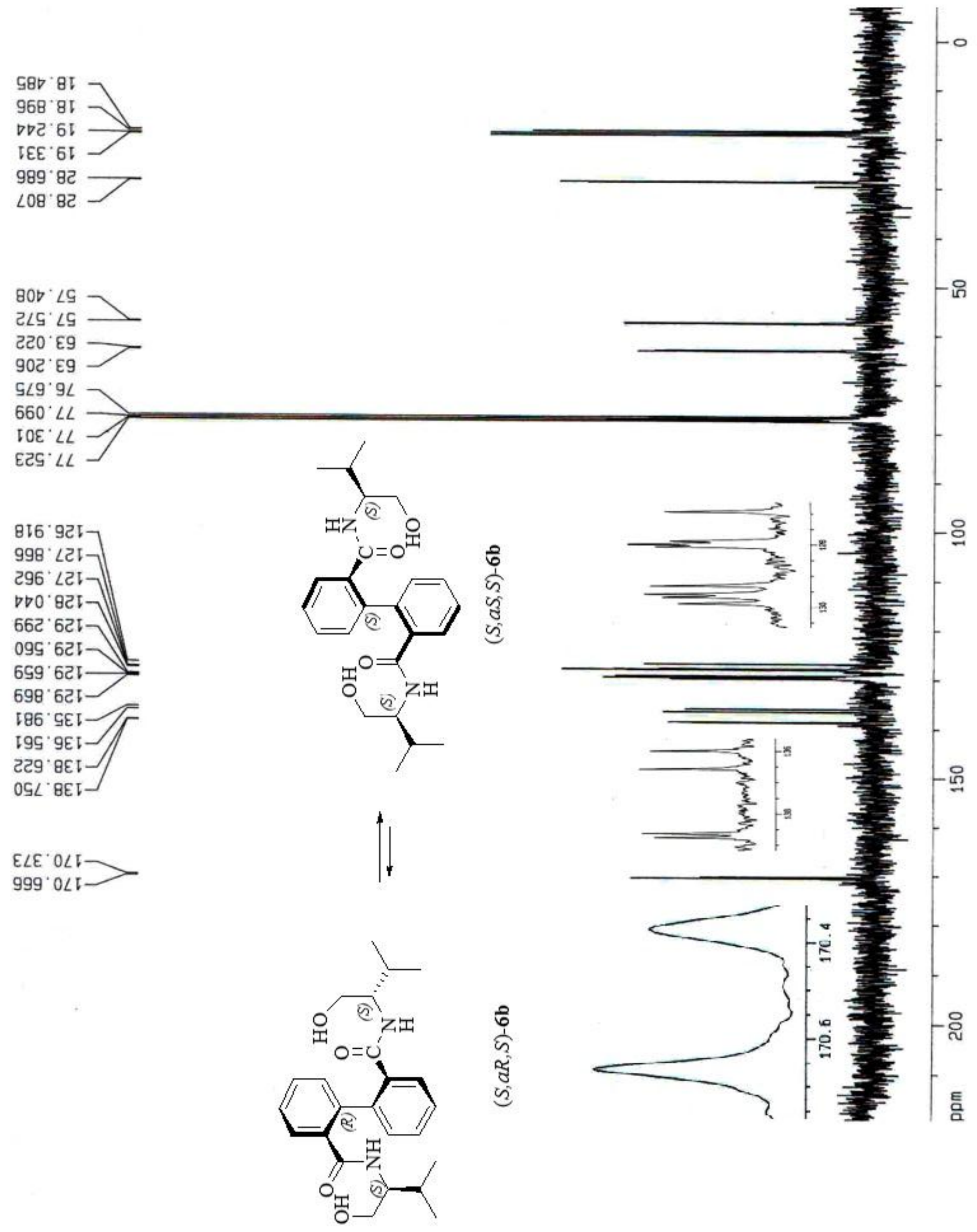

Figure S7: ^{13}CNMR of **6b**

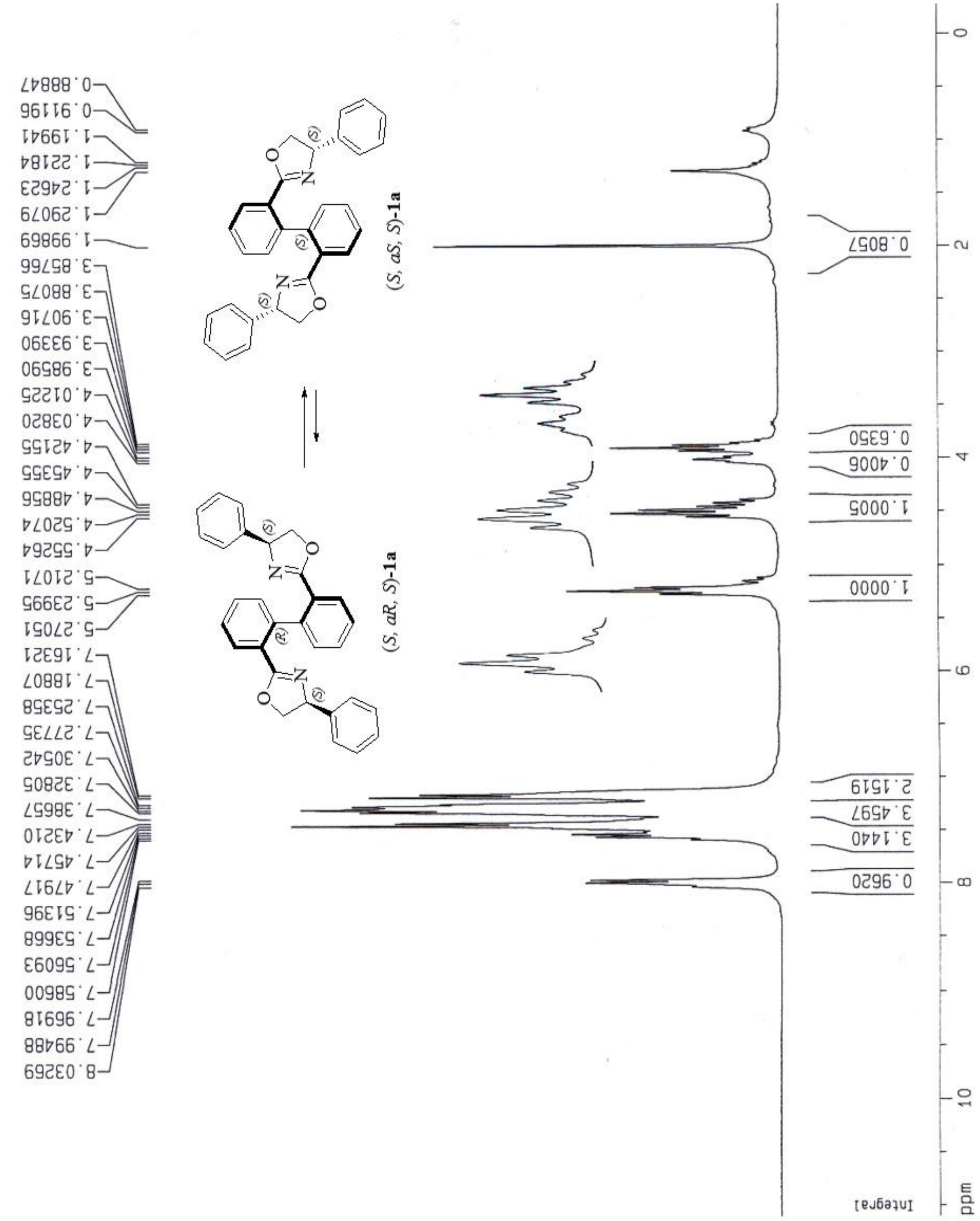

Figure S8: ^{1}HNMR of **1a**

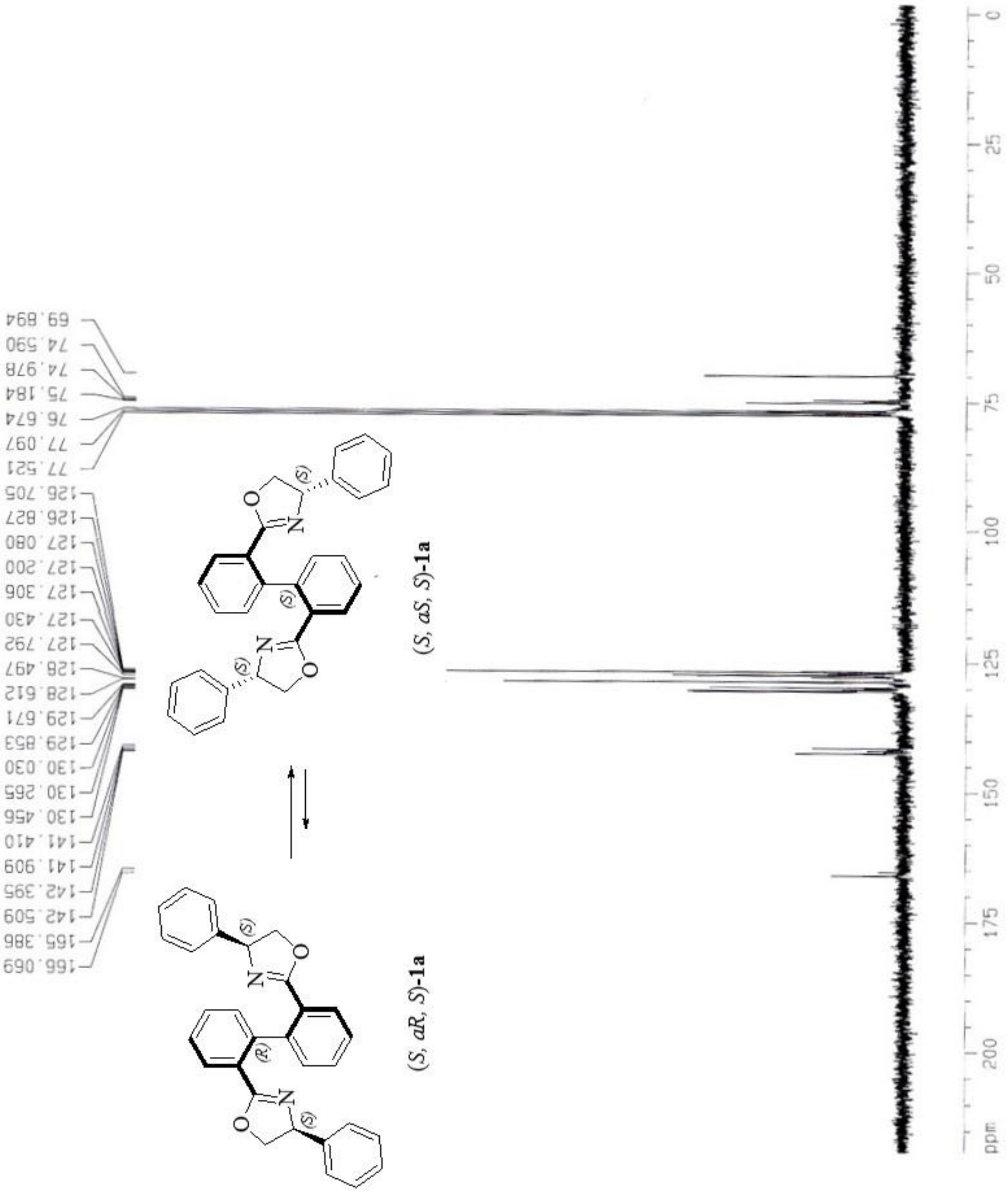

Figure S9: ^{13}CNMR of **1a**

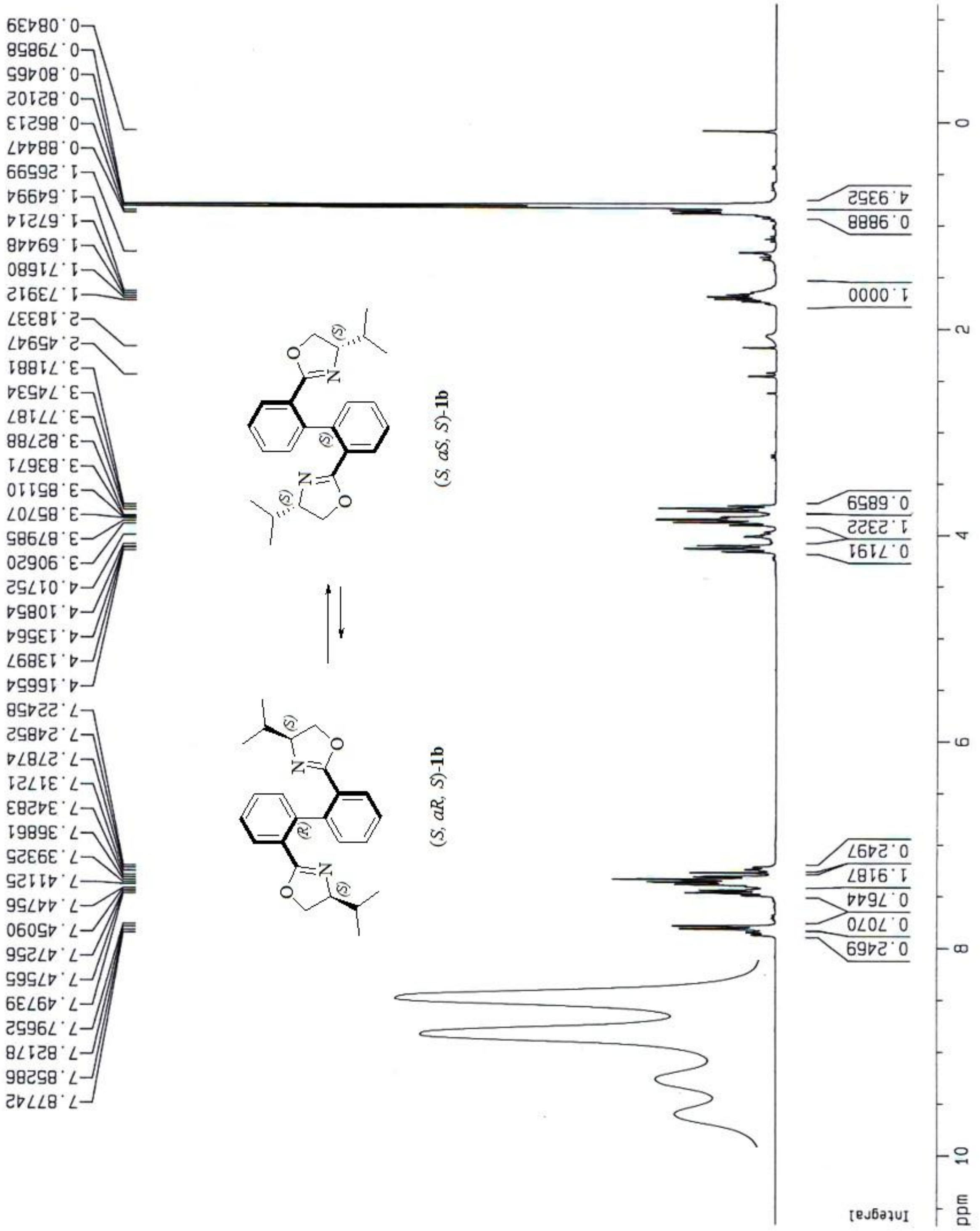

Figure S10: ^{1}HNMR of **1b**

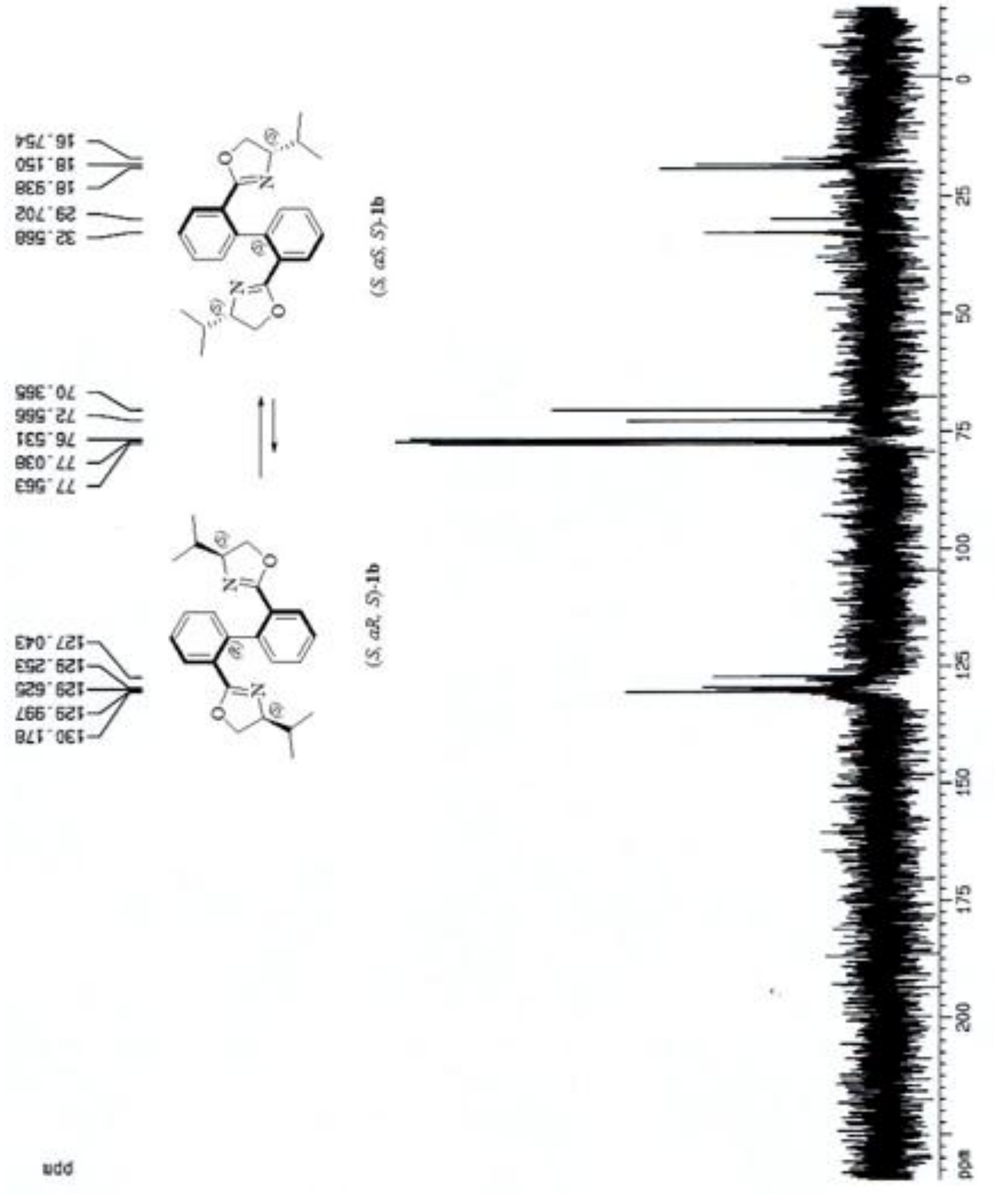

Figure S11: ^{13}CNMR of **1b**

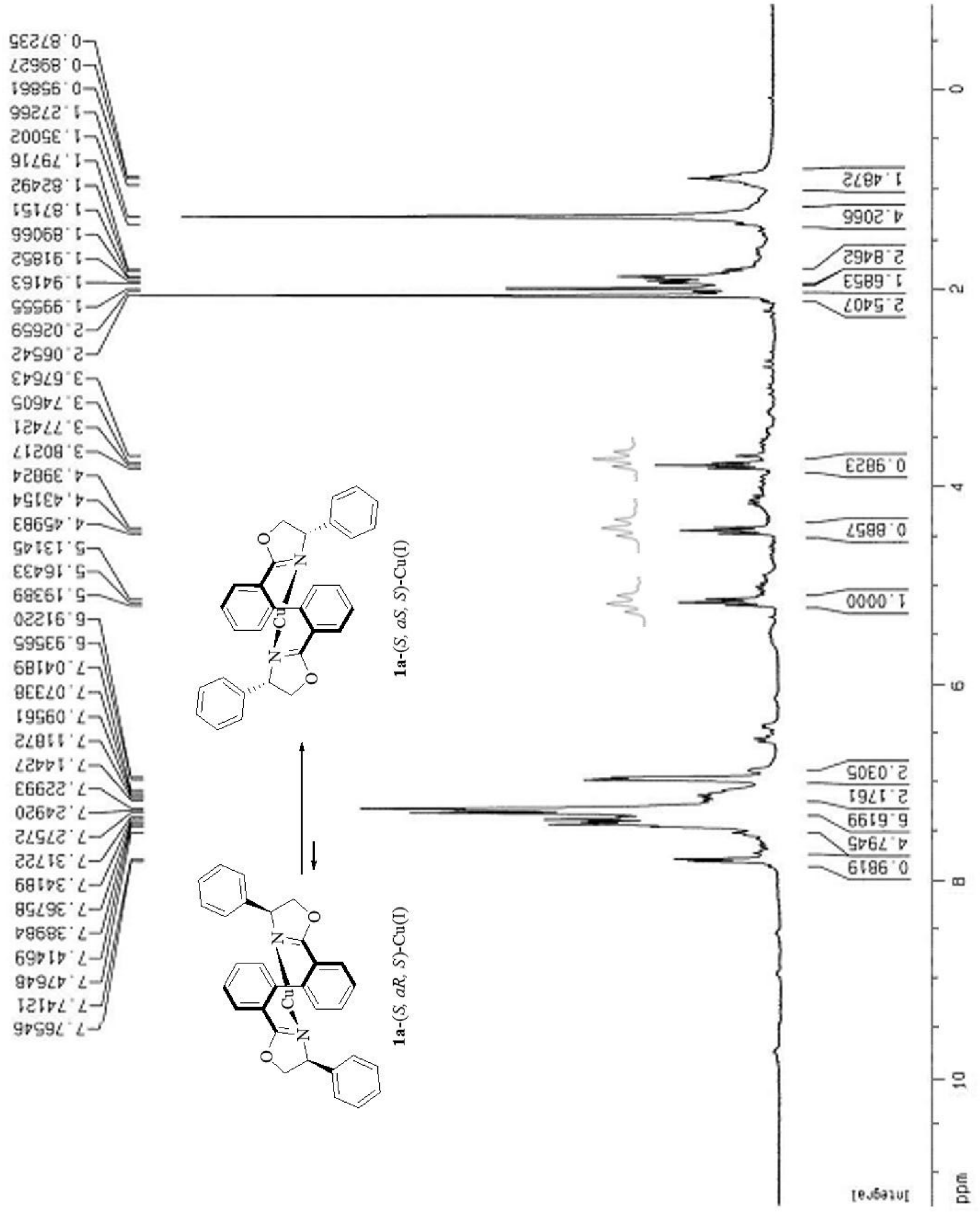

Figure S12: ^{1}HNMR of **1a-Cu**

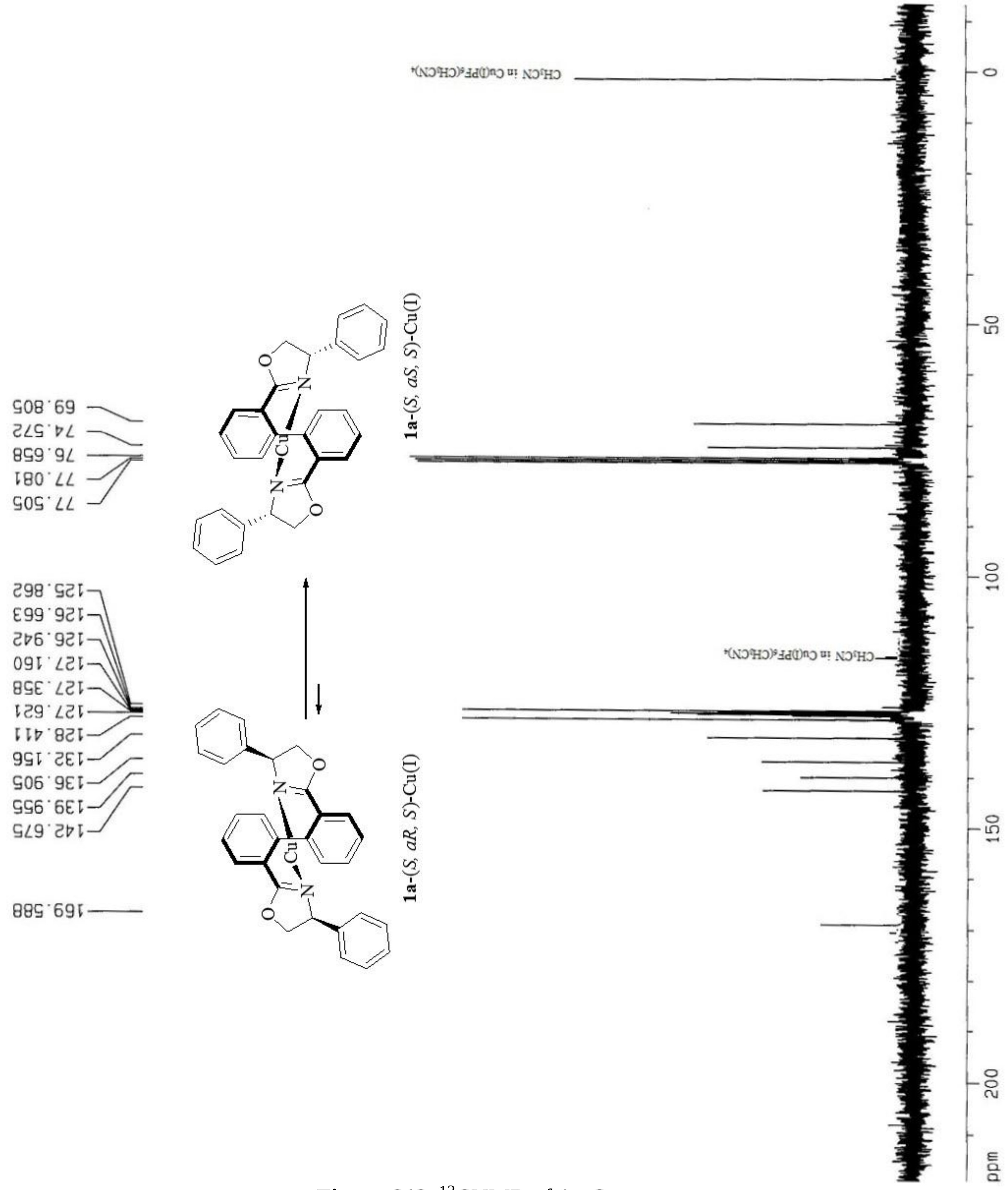

Figure S13: ^{13}CNMR of **1a-Cu**

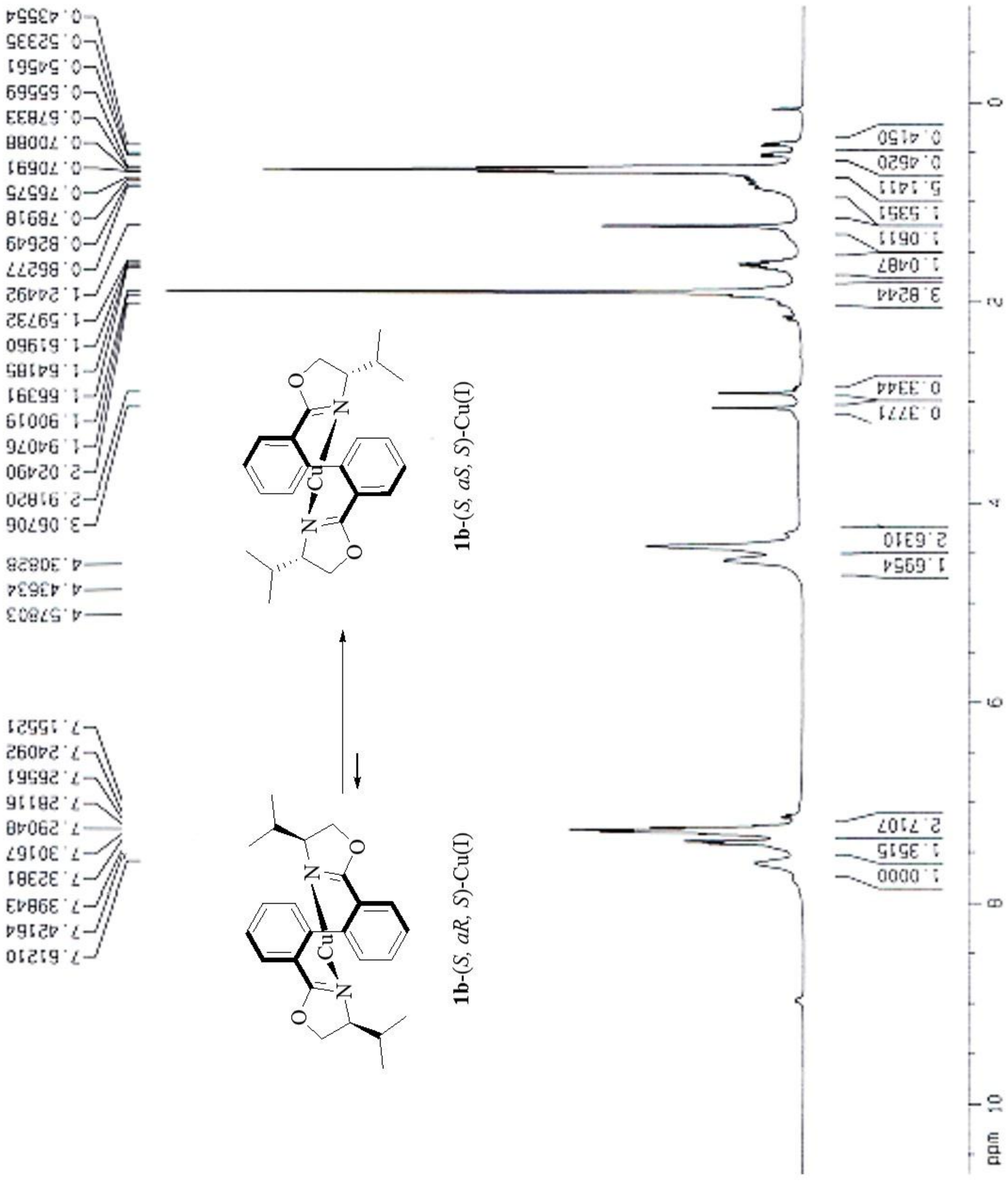

Figure S14: ^{1}HNMR of **1b-Cu**

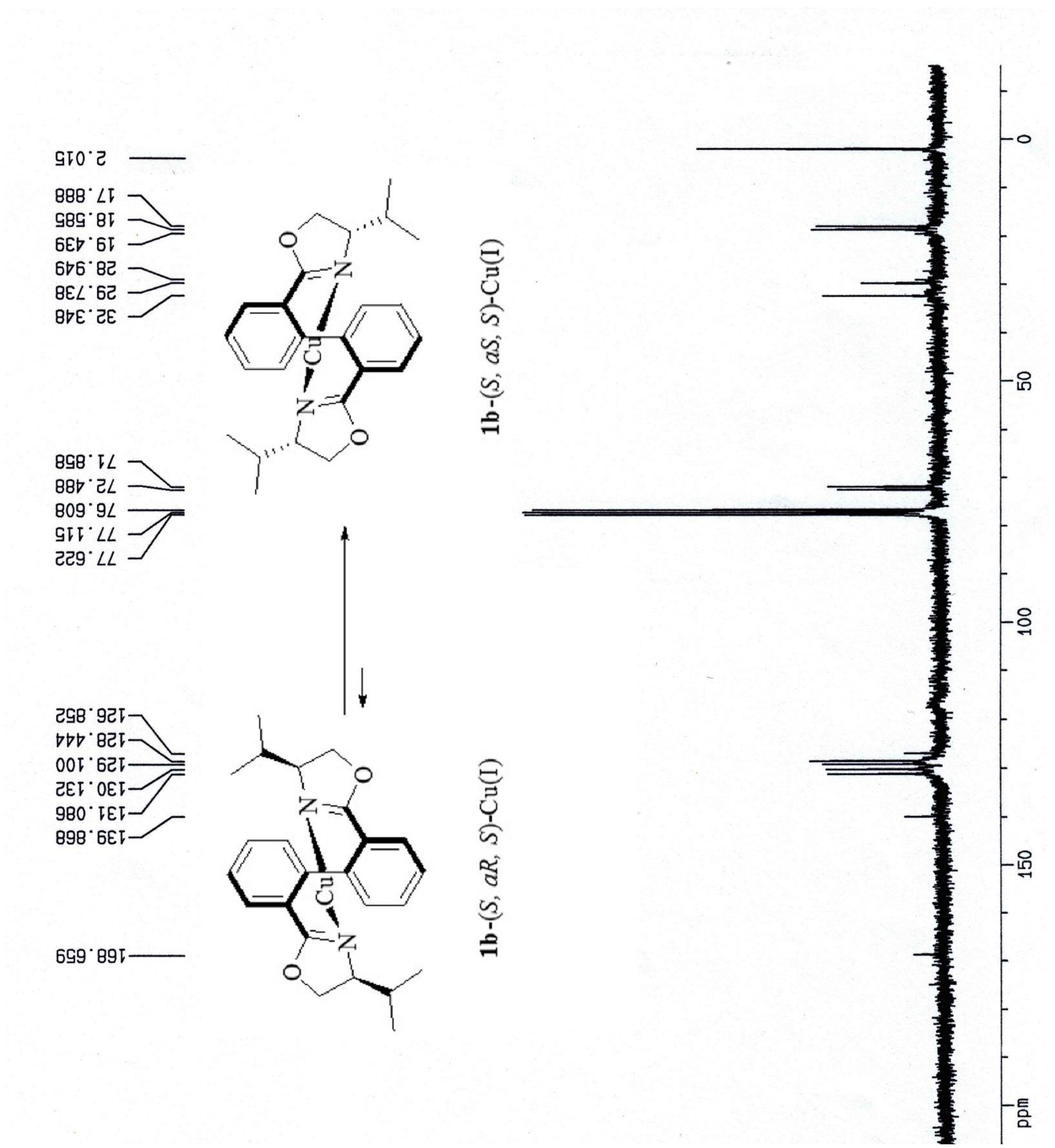

Figure S15: ^{13}CNMR of **1b-Cu**

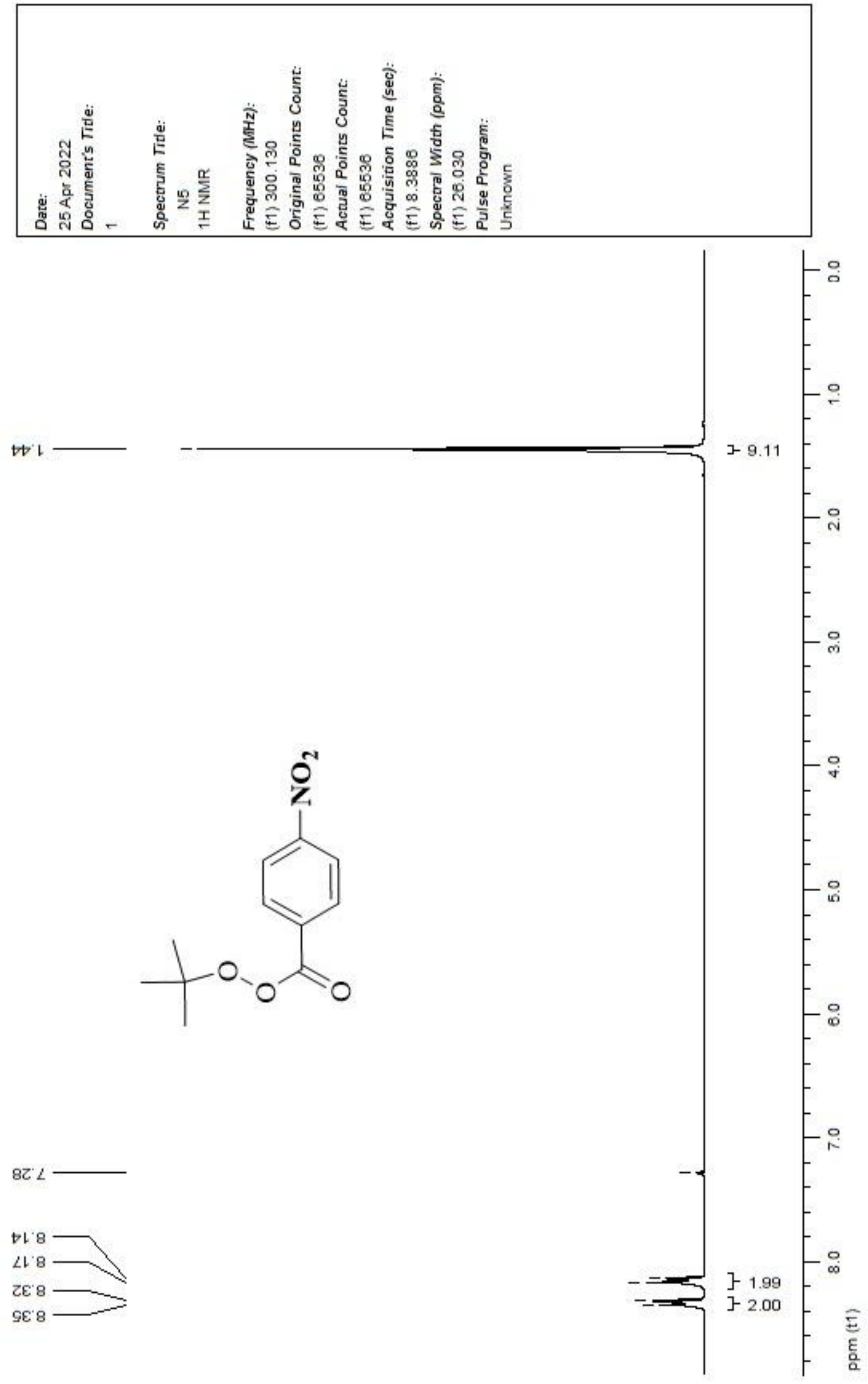

Figure S16: ^{1}HNMR of **7a**

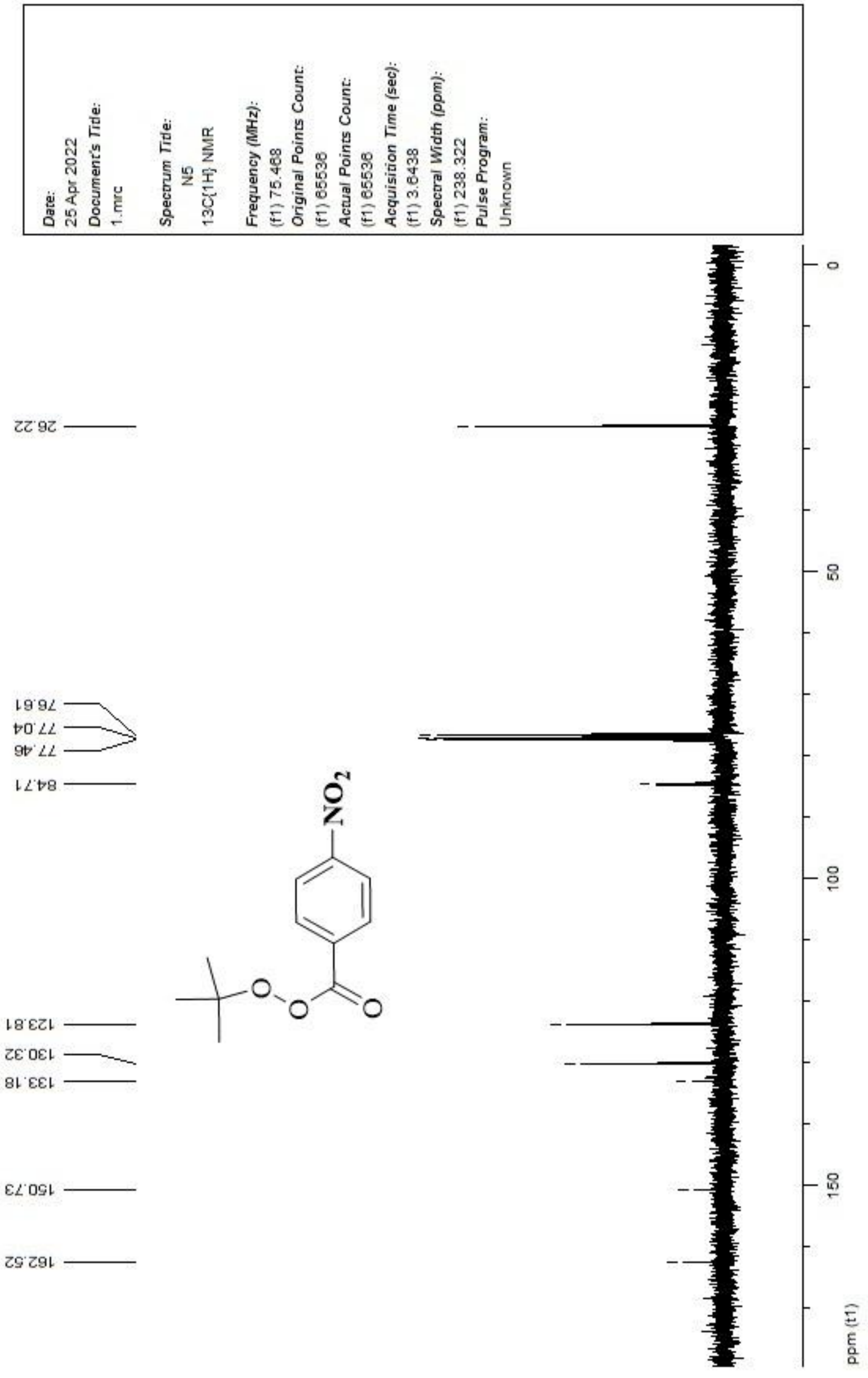

Figure S17: ^{13}CNMR of **7a**

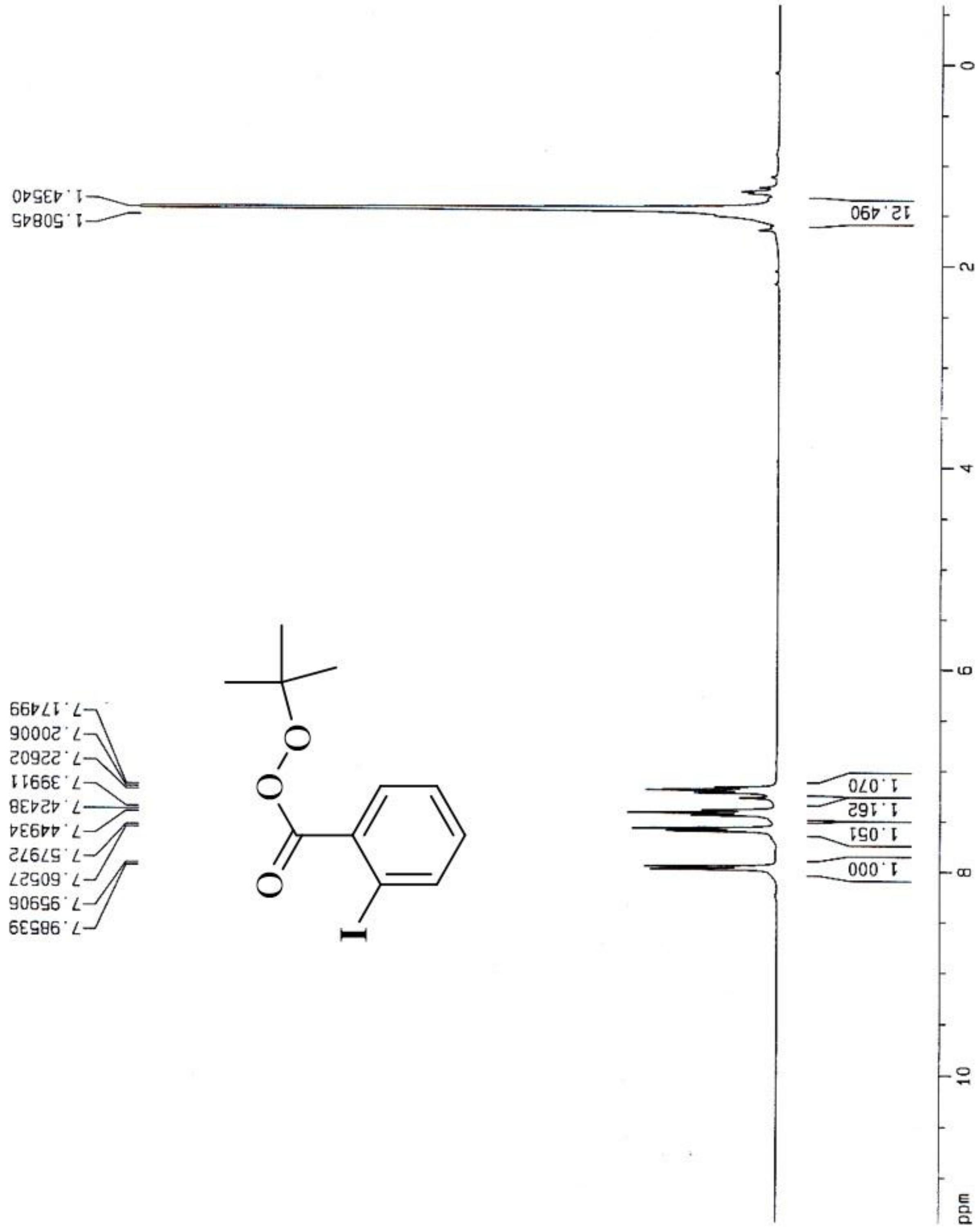

Figure S18: ^{1}HNMR of **7d**

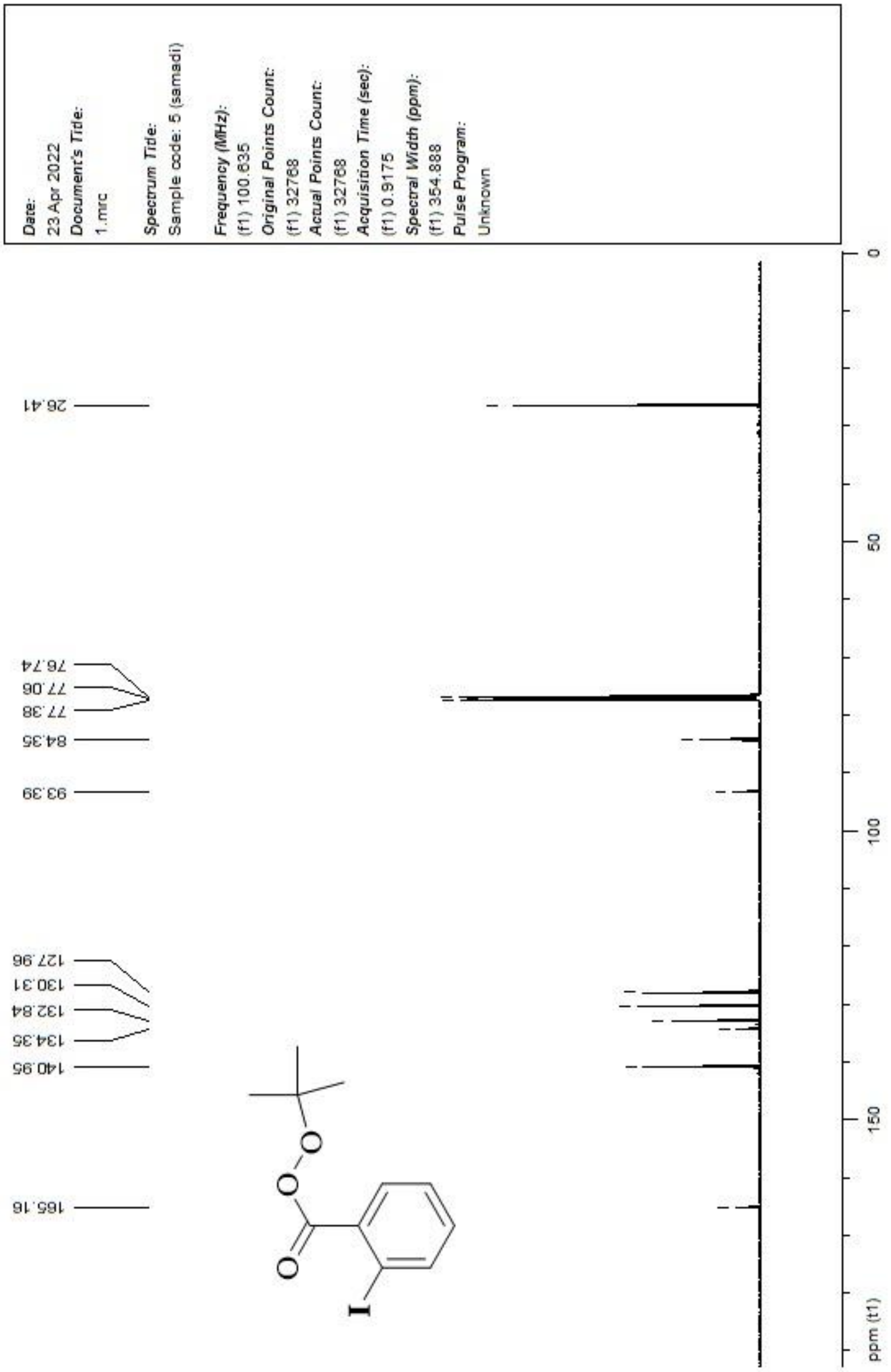

Figure S19: ^{13}CNMR of **7d**

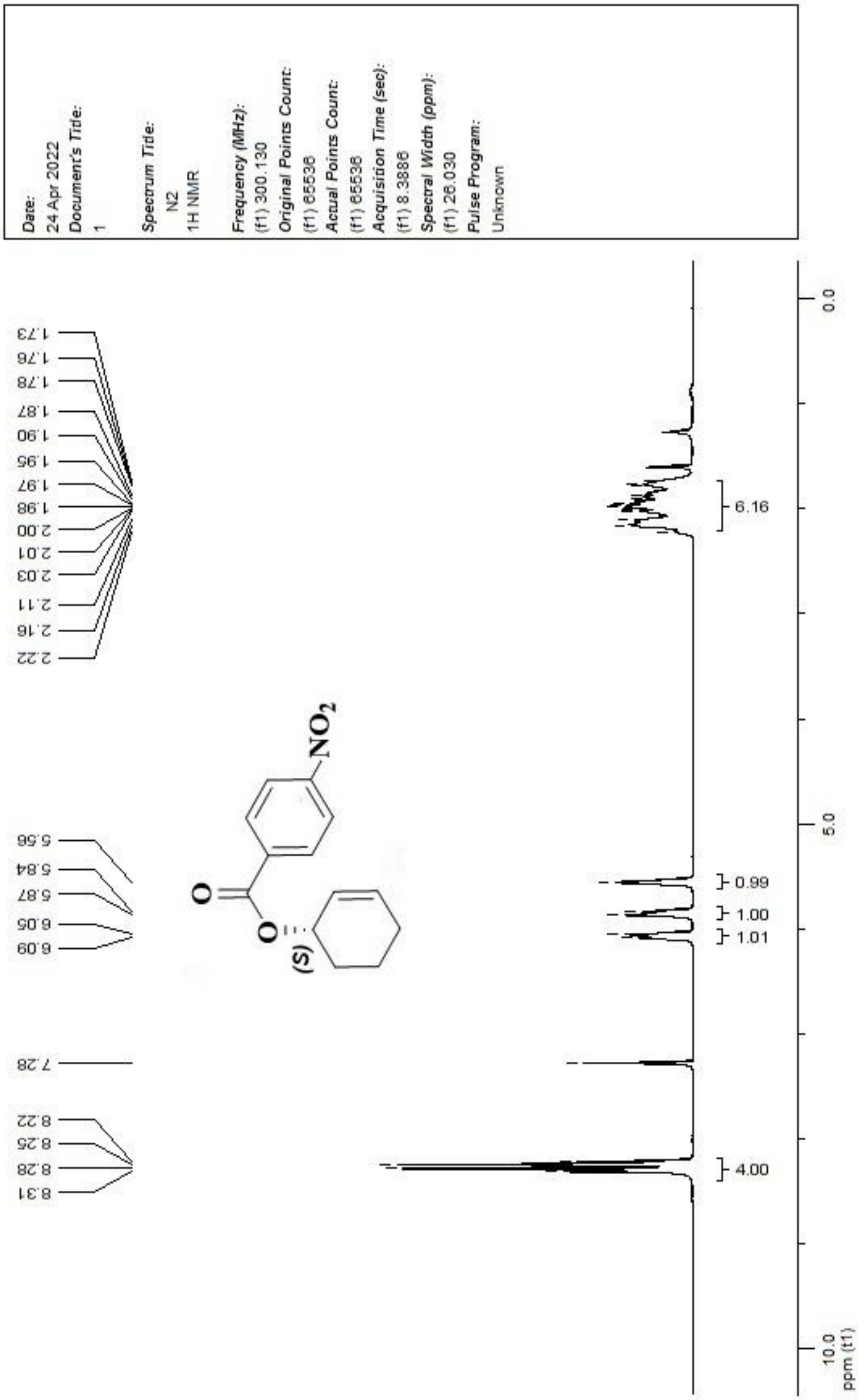

Figure S20: ^{1}HNMR of **8a**

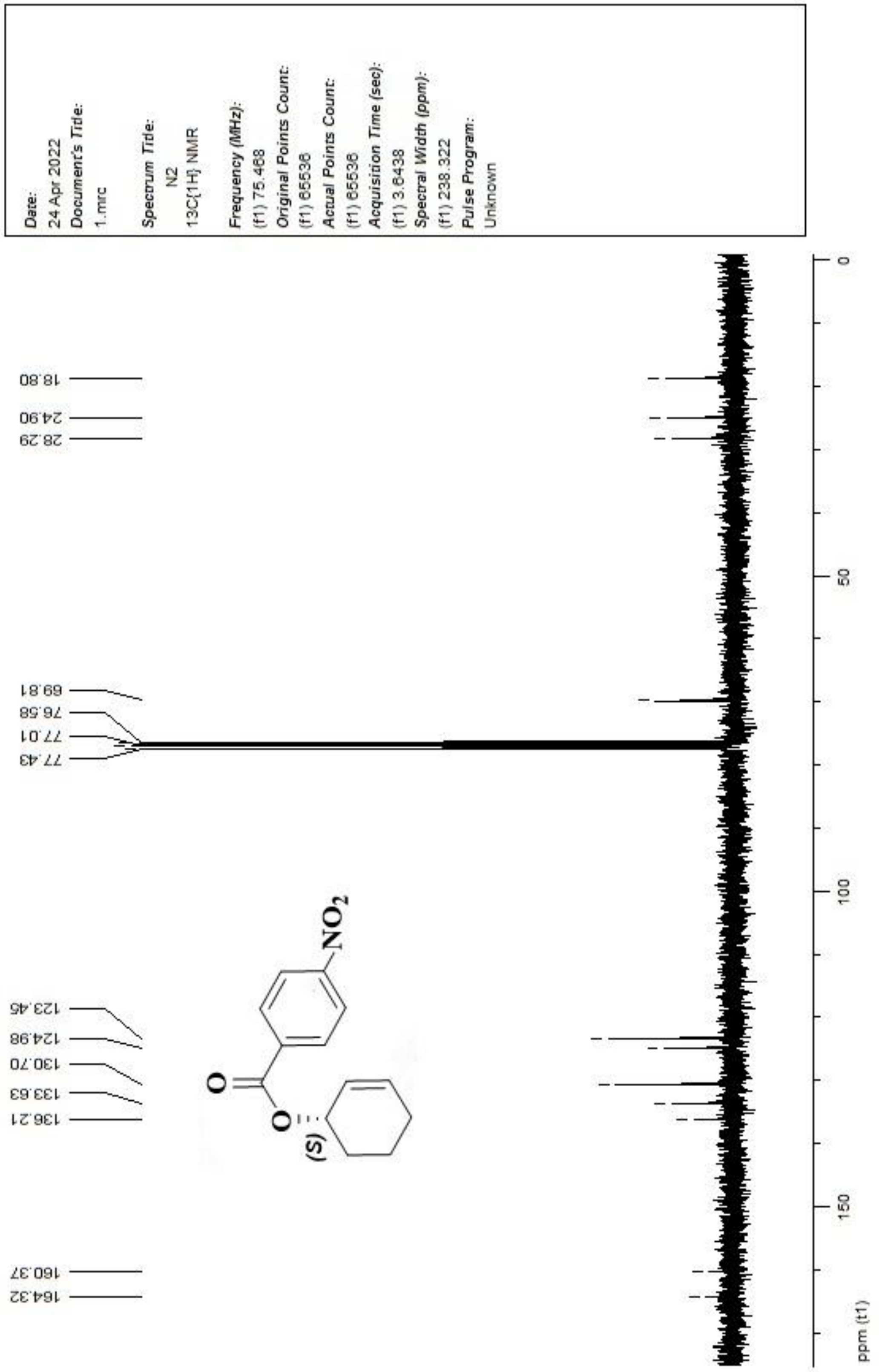

Figure S21: ^{13}CNMR of **8a**

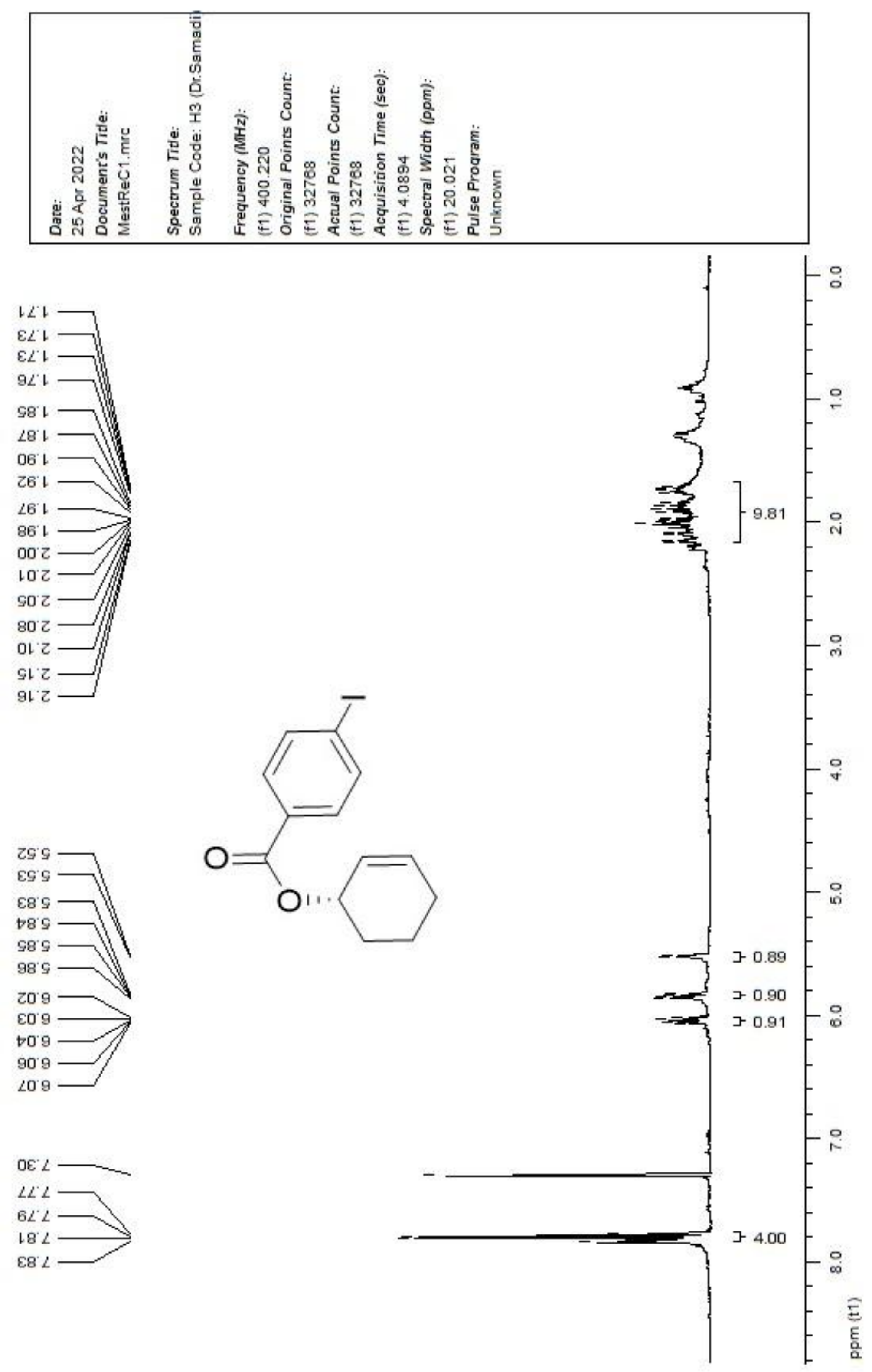

Figure S22: ^{1}HNMR of **8b**

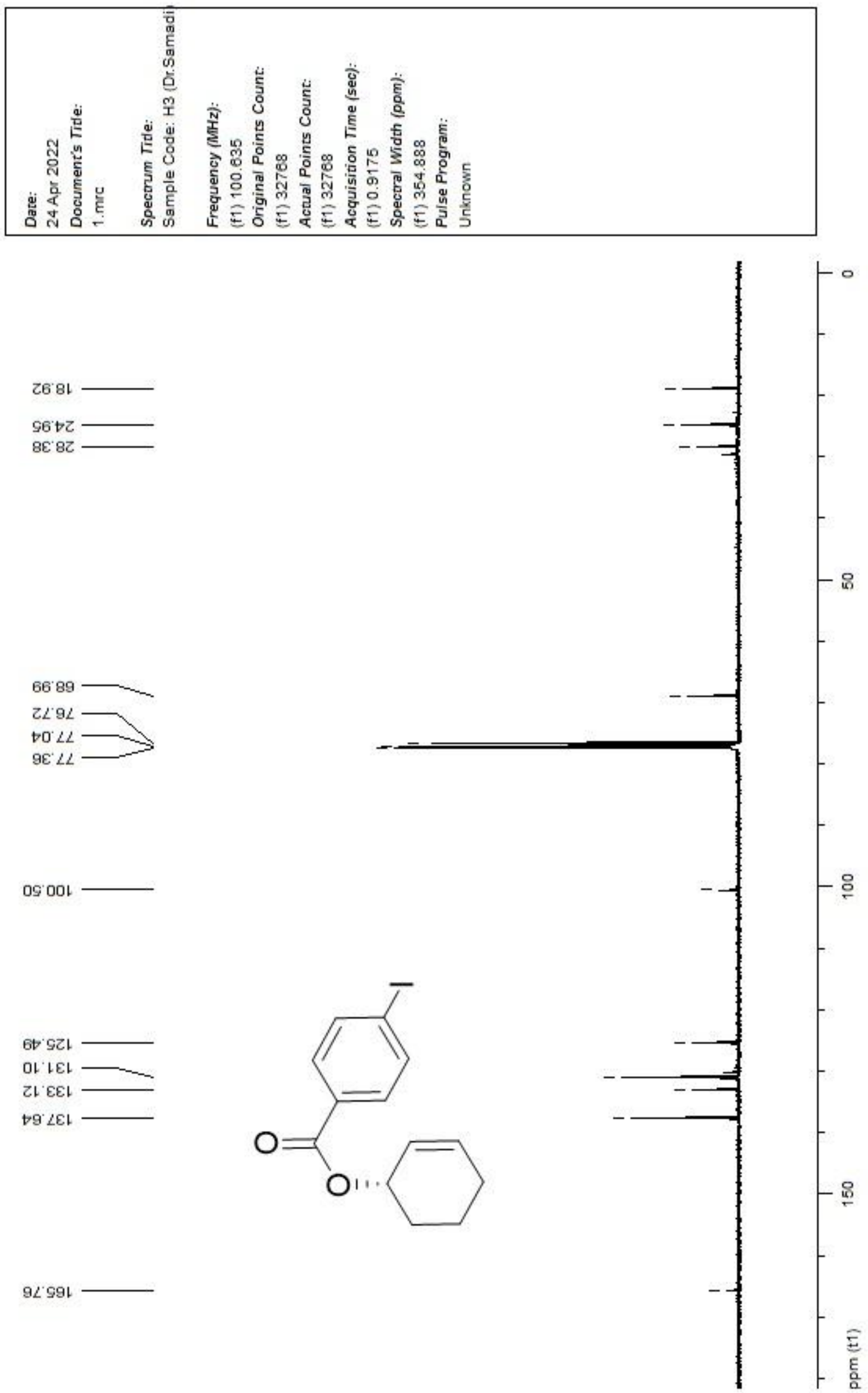

Figure S23: ^{13}CNMR of **8b**

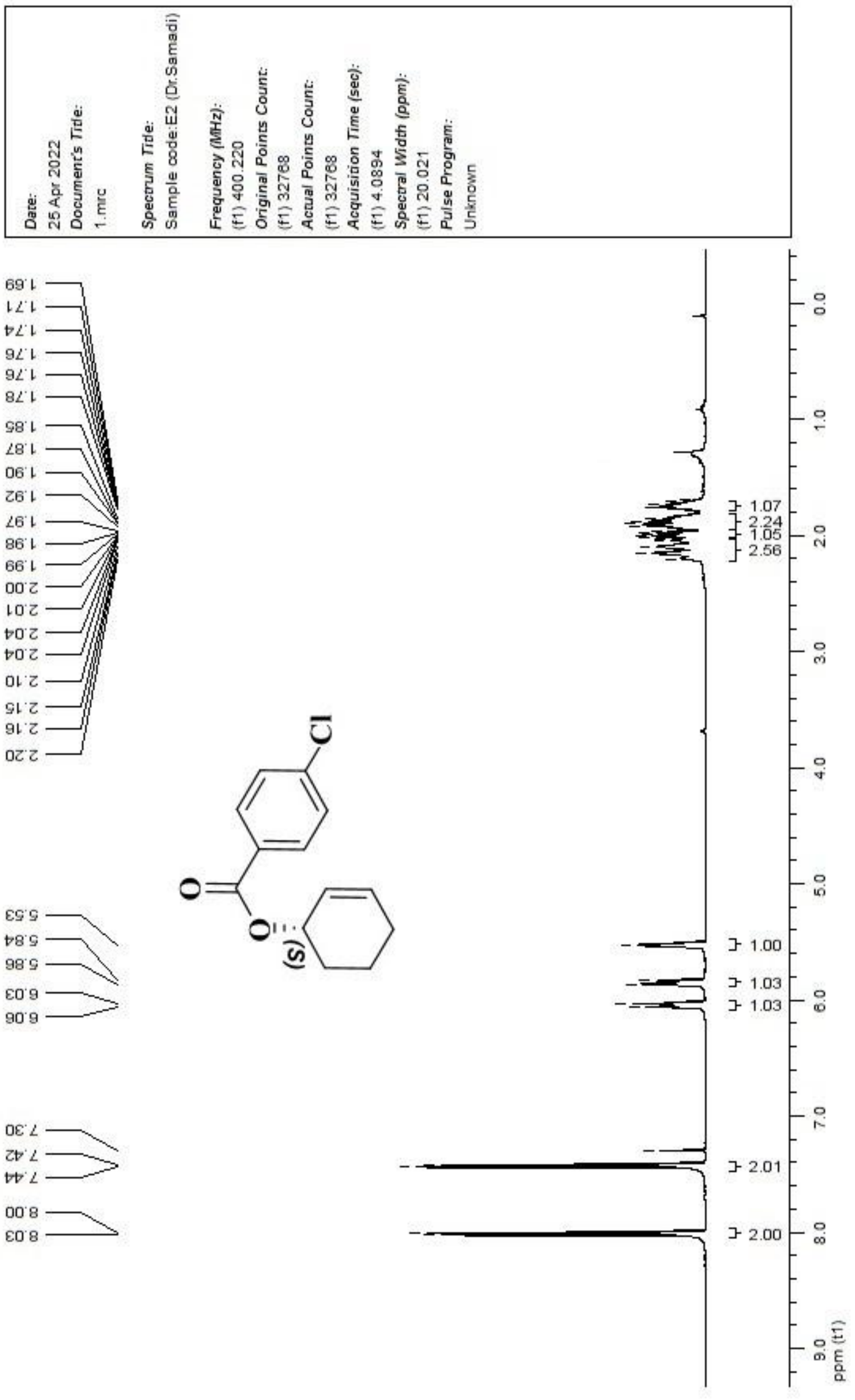

Figure S24: ^{1}HNMR of **8c**

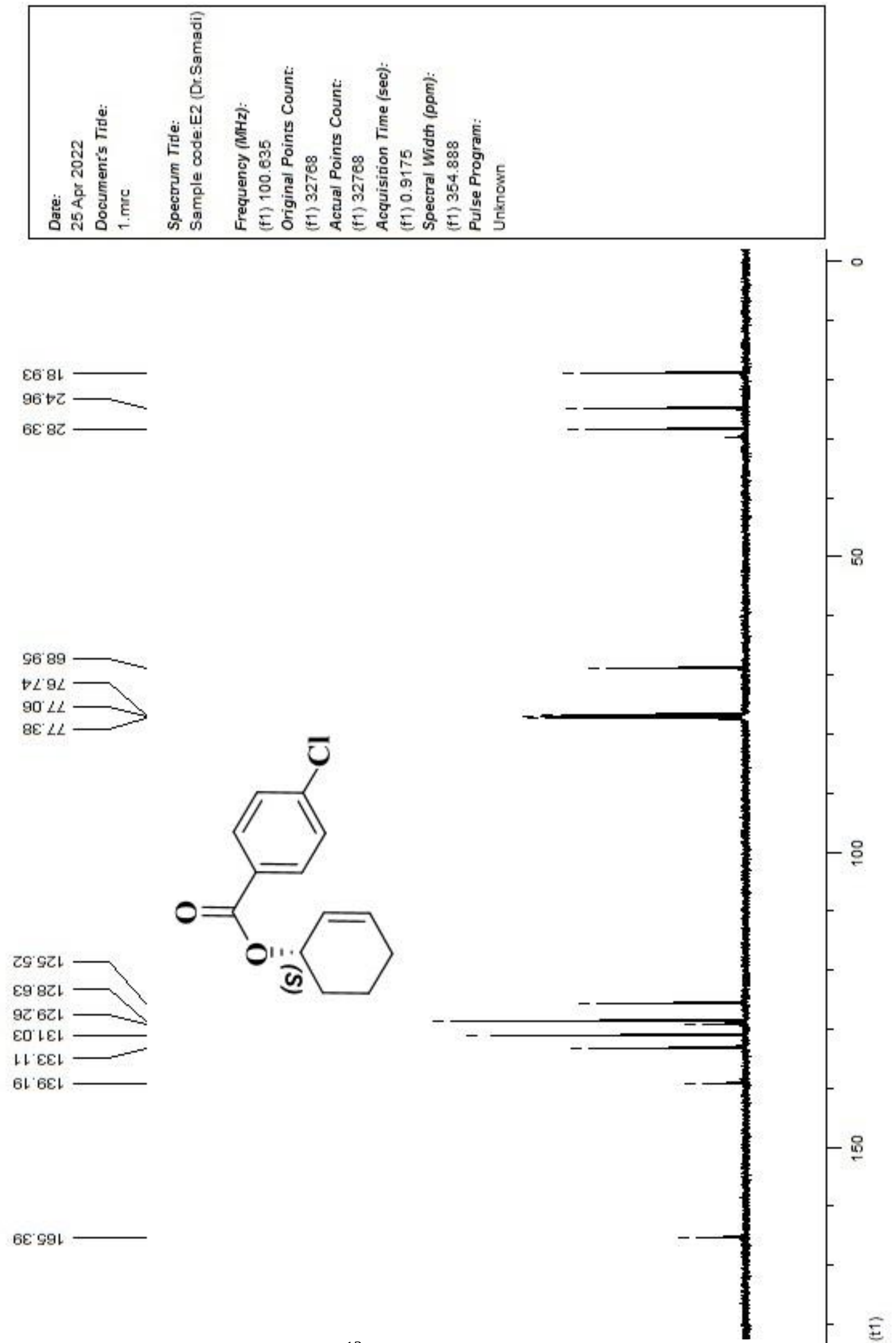

Figure S25: ^{13}CNMR of **8c**

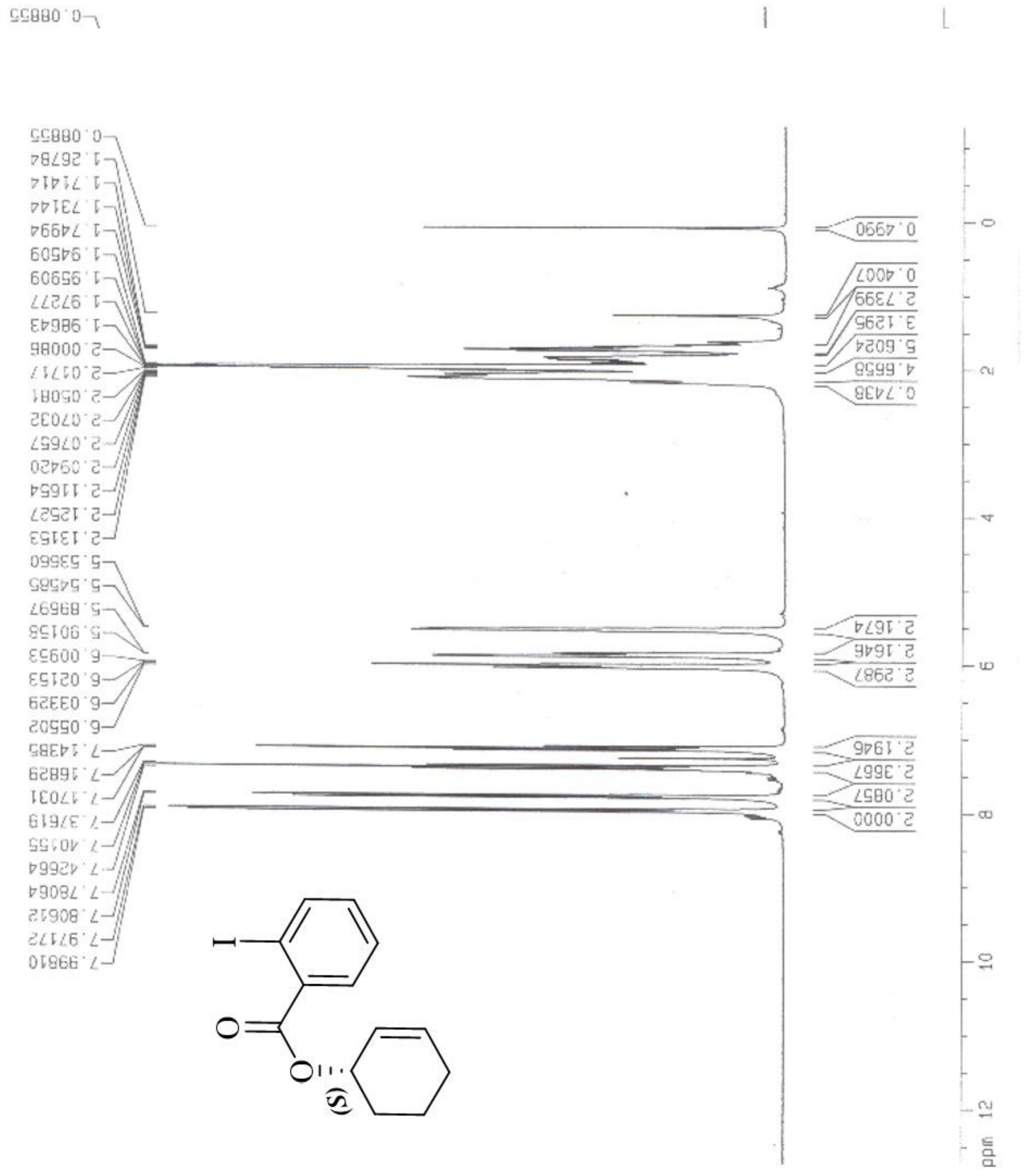

Figure S26: ^{1}HNMR of **8d**

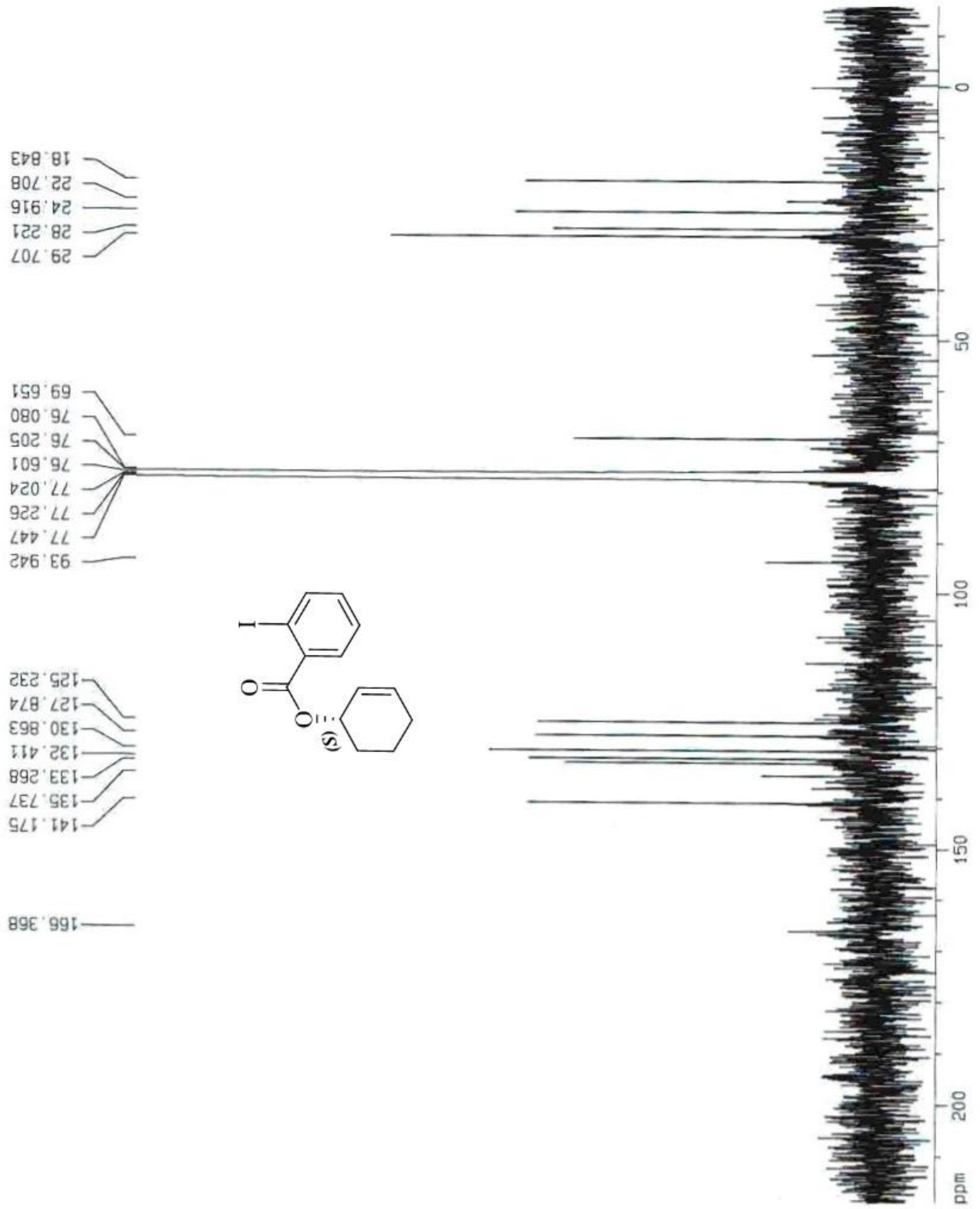

Figure S27: ^{13}CNMR of **8d**

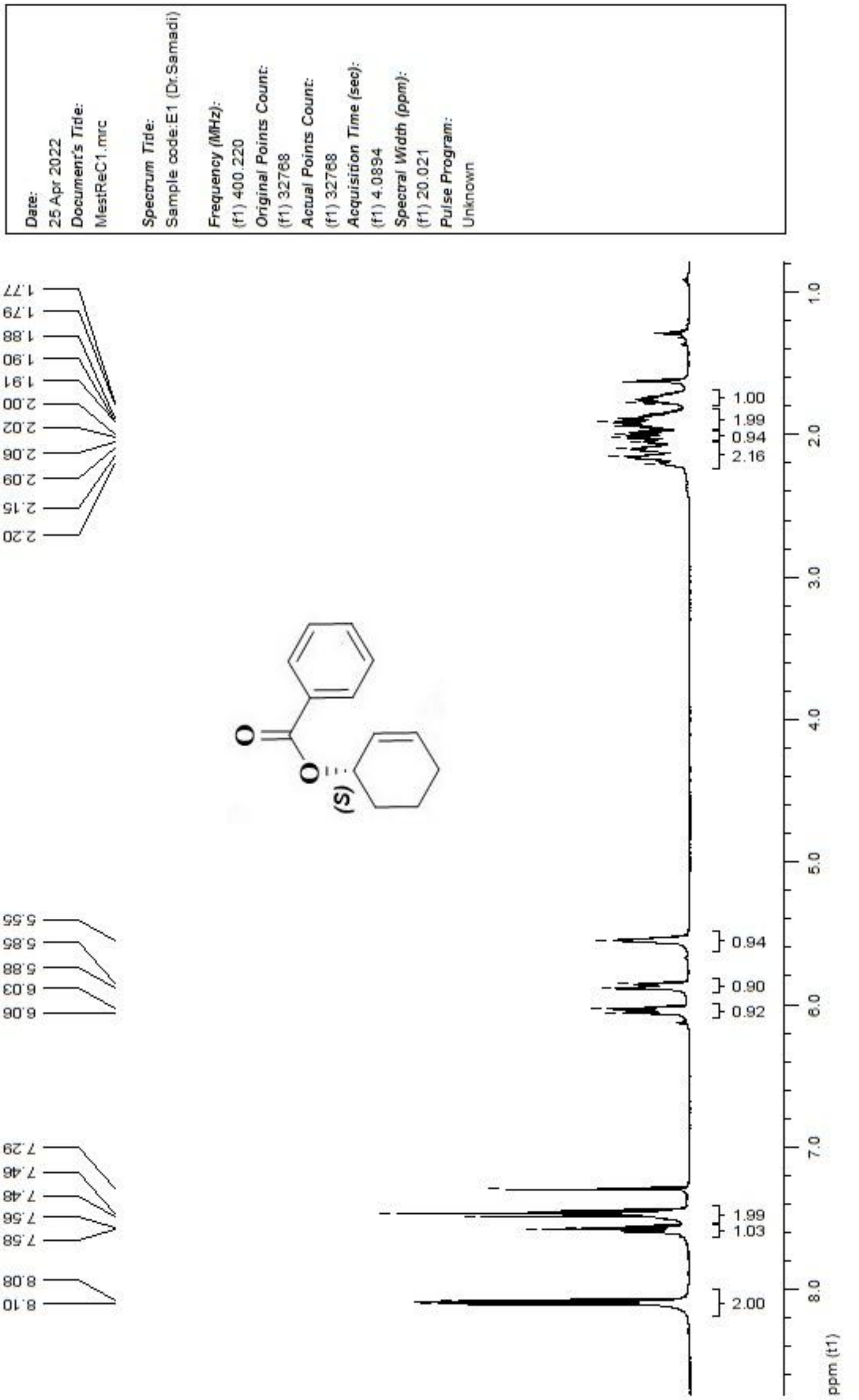

Figure S28: ^{1}HNMR of **8g**

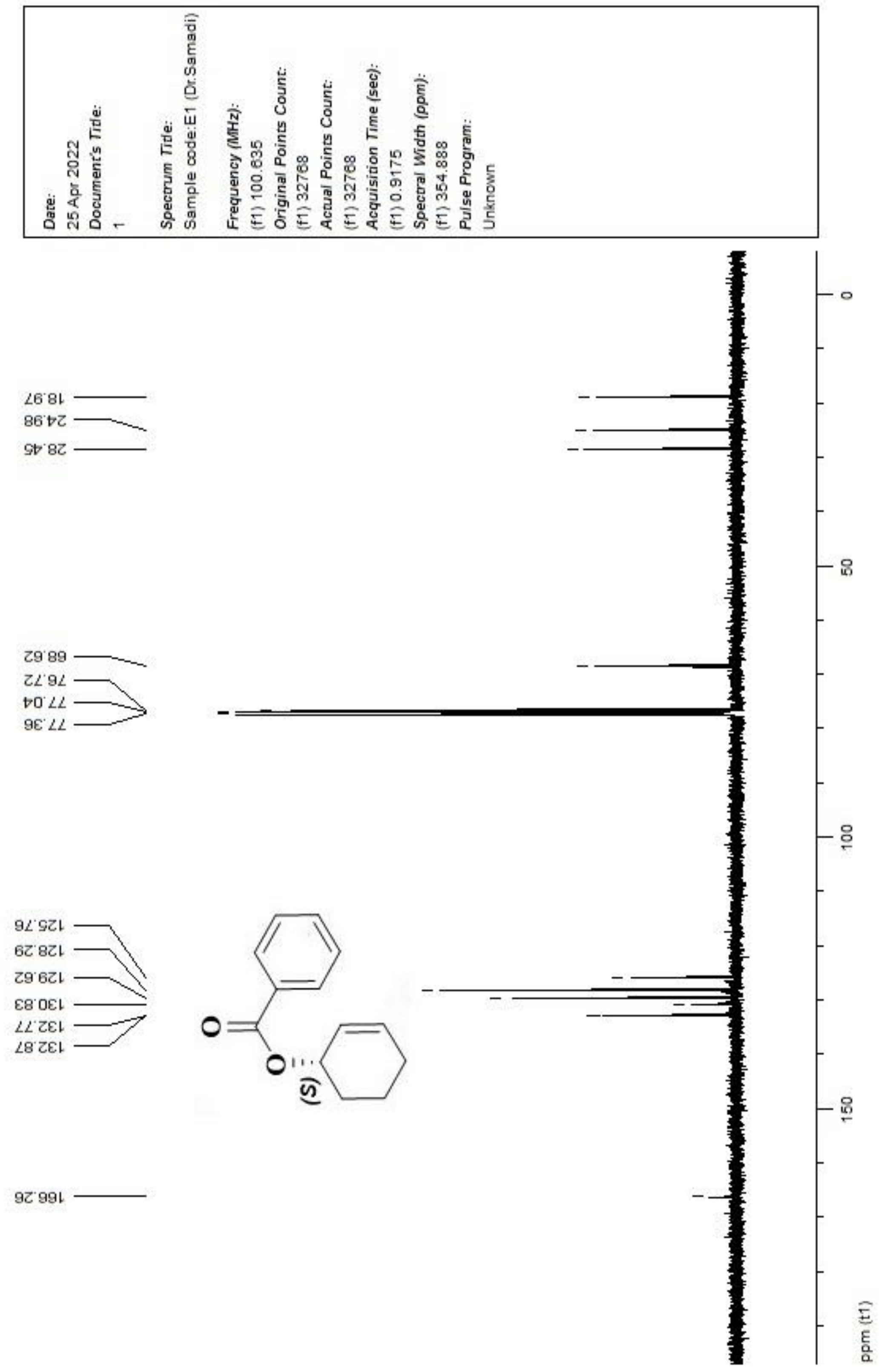

Figure S29: ^{13}CNMR of **8g**

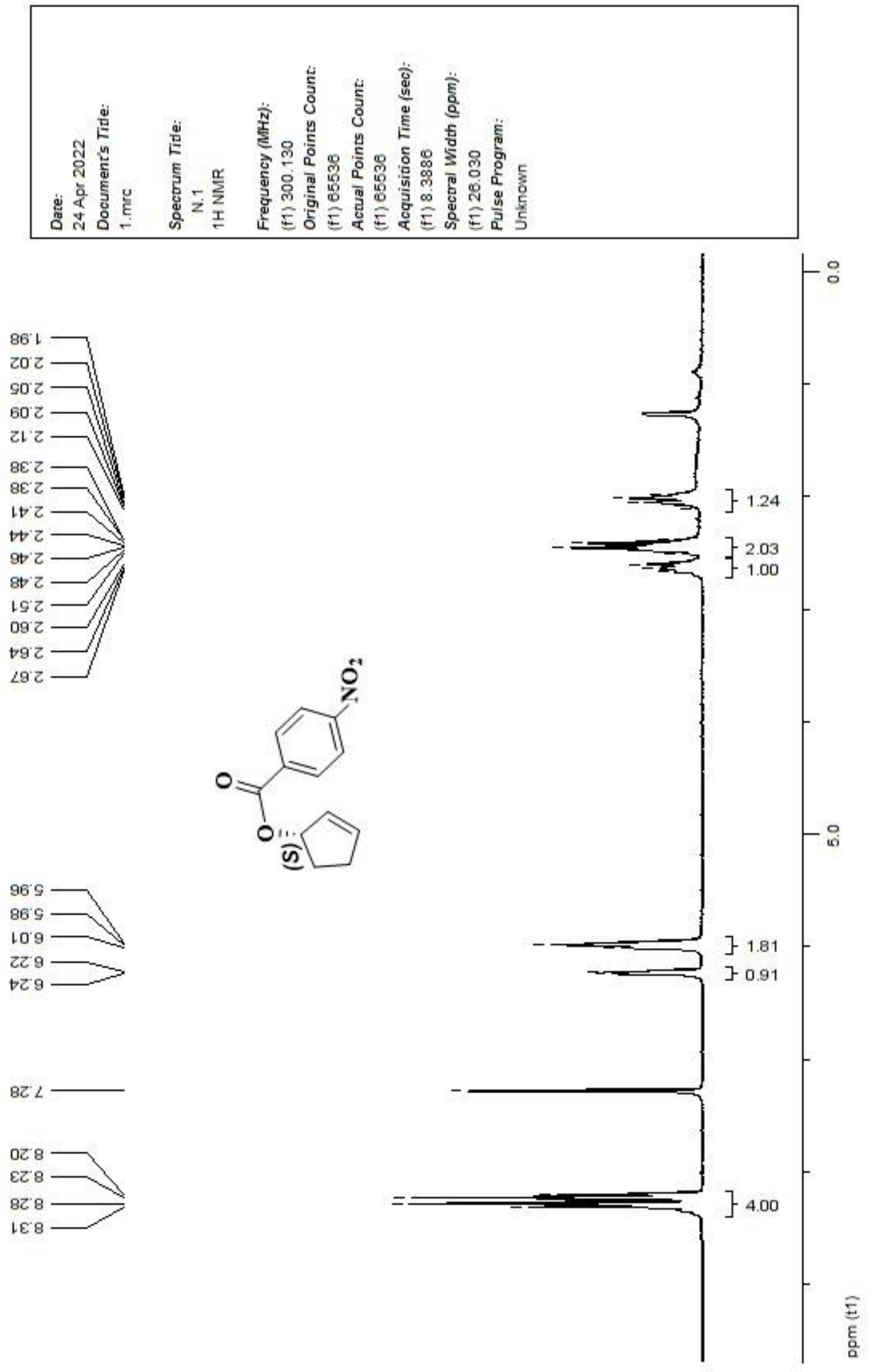

Figure S30: ^{1}HNMR of **9a**

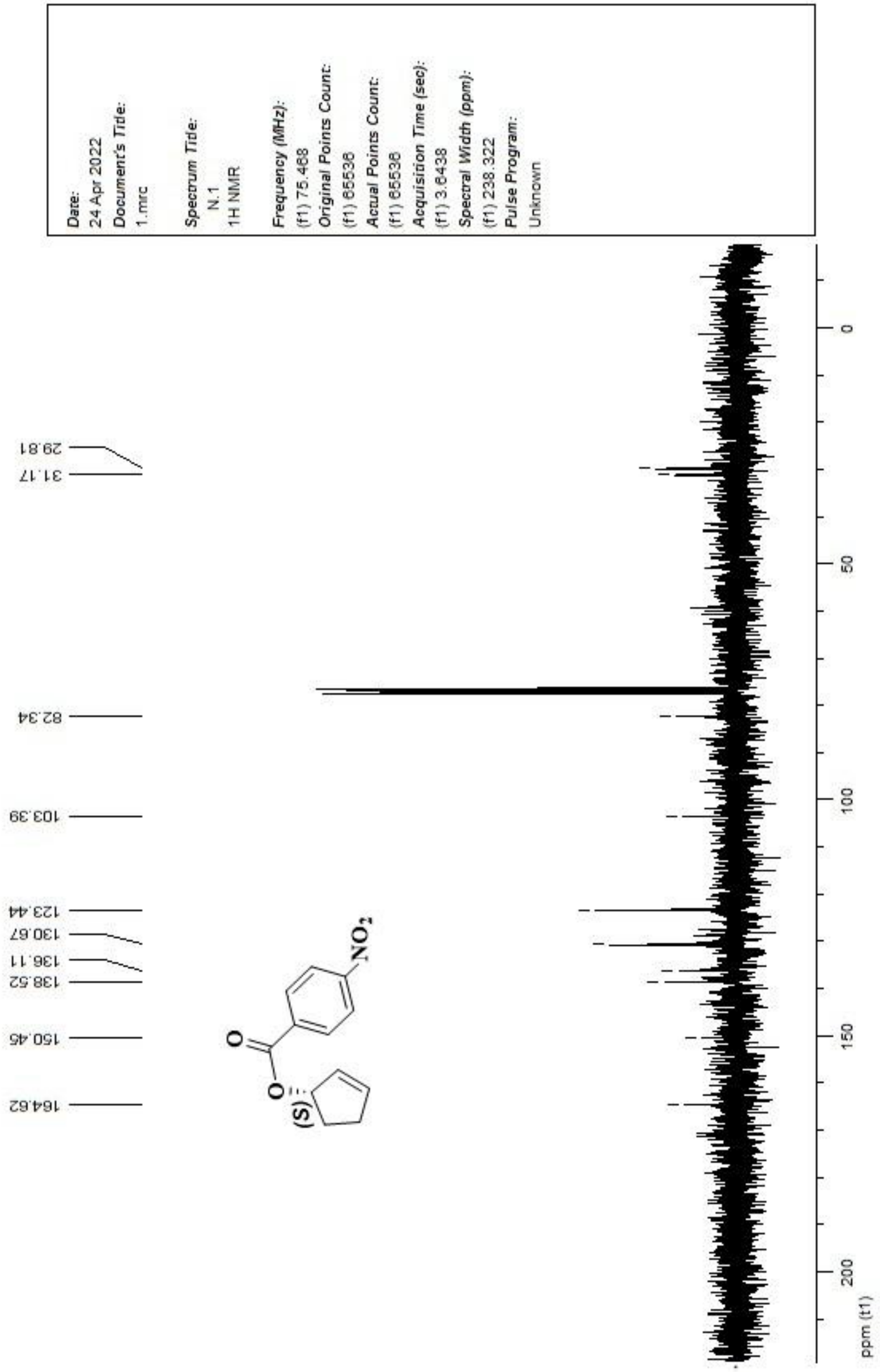

Figure S31: ^{13}CNMR of **9a**

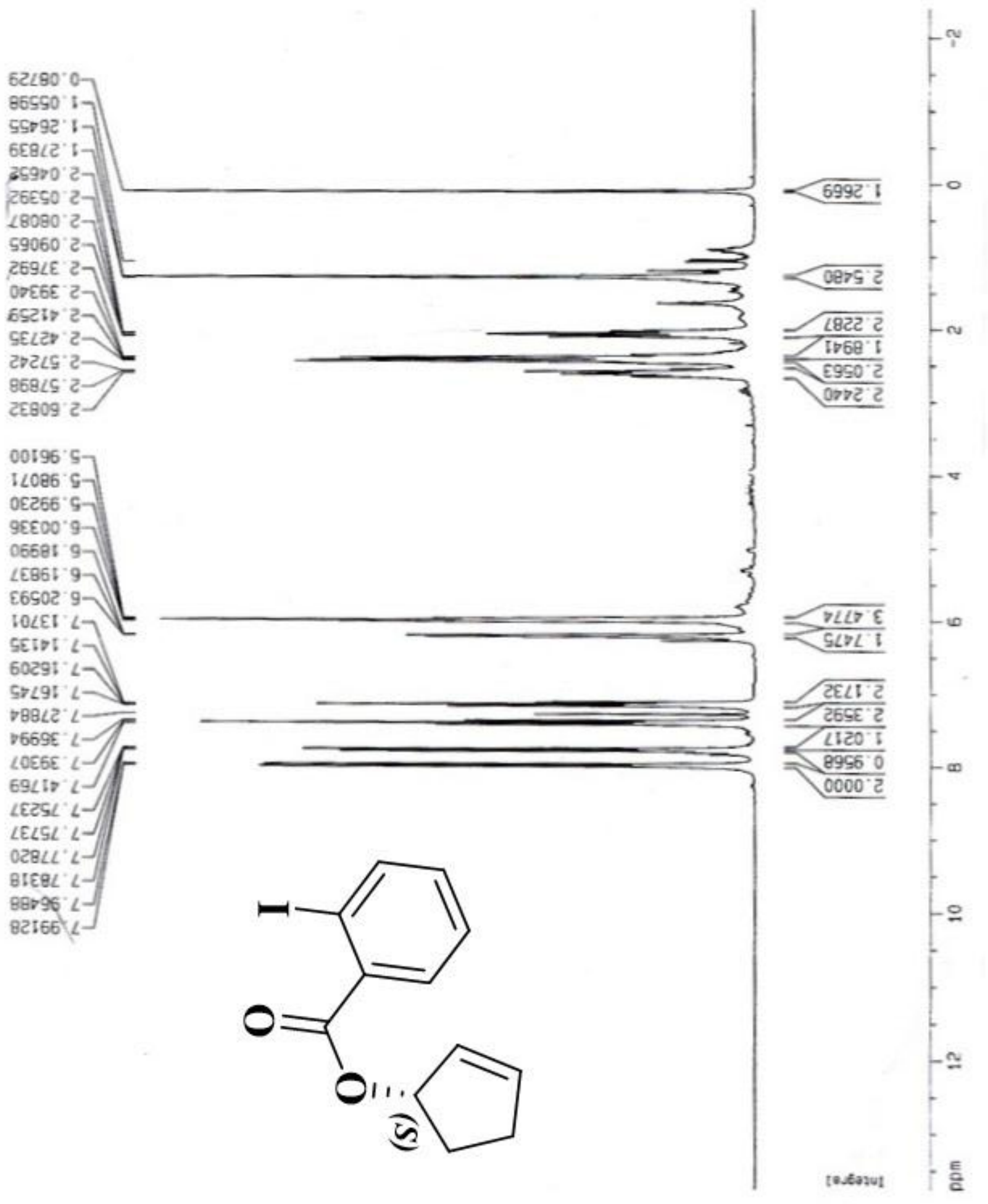

Figure S32: ^{1}HNMR of **9d**

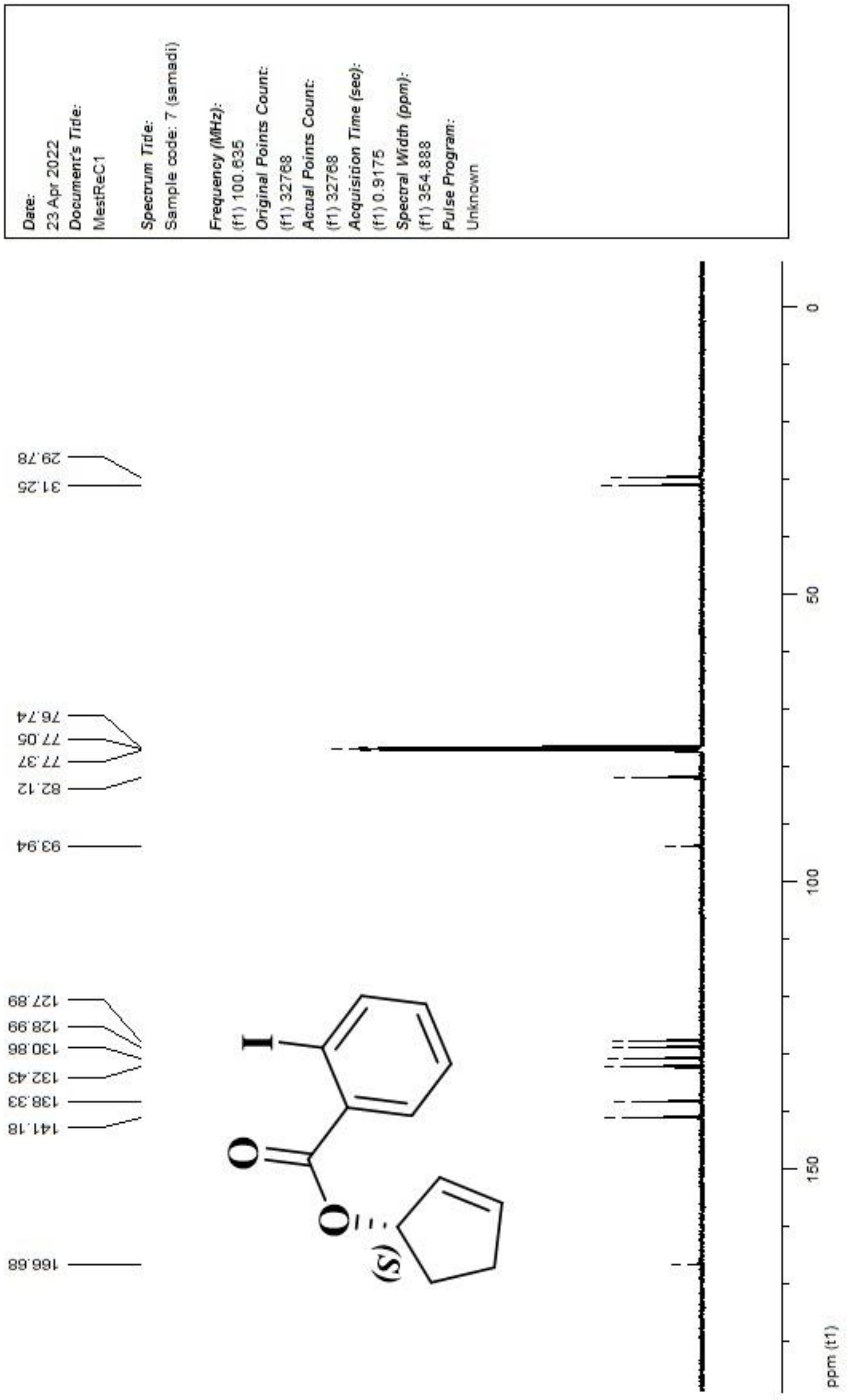

Figure S33: ^{13}CNMR of **9d**

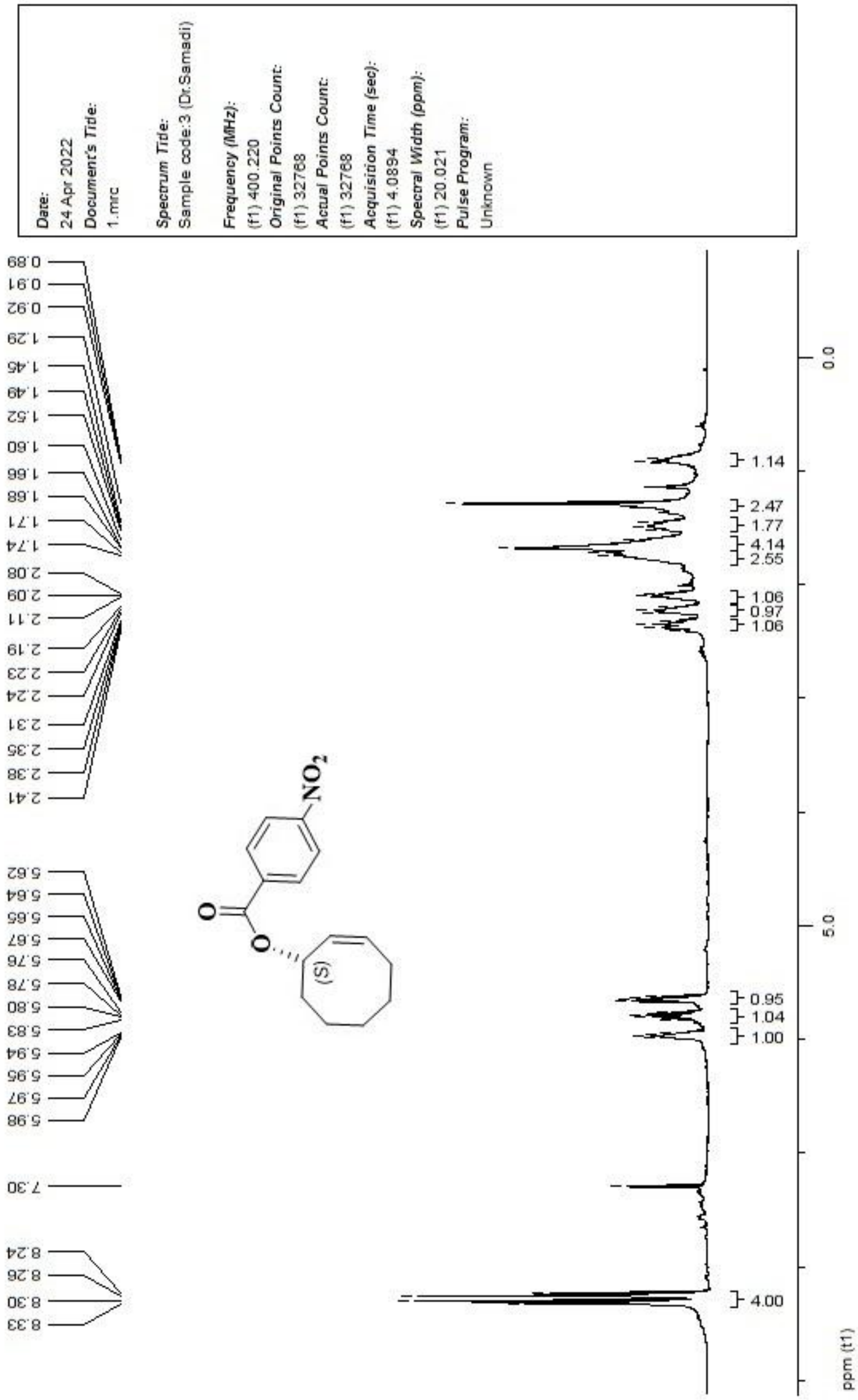

Figure S34: ^{1}HNMR of **10a**

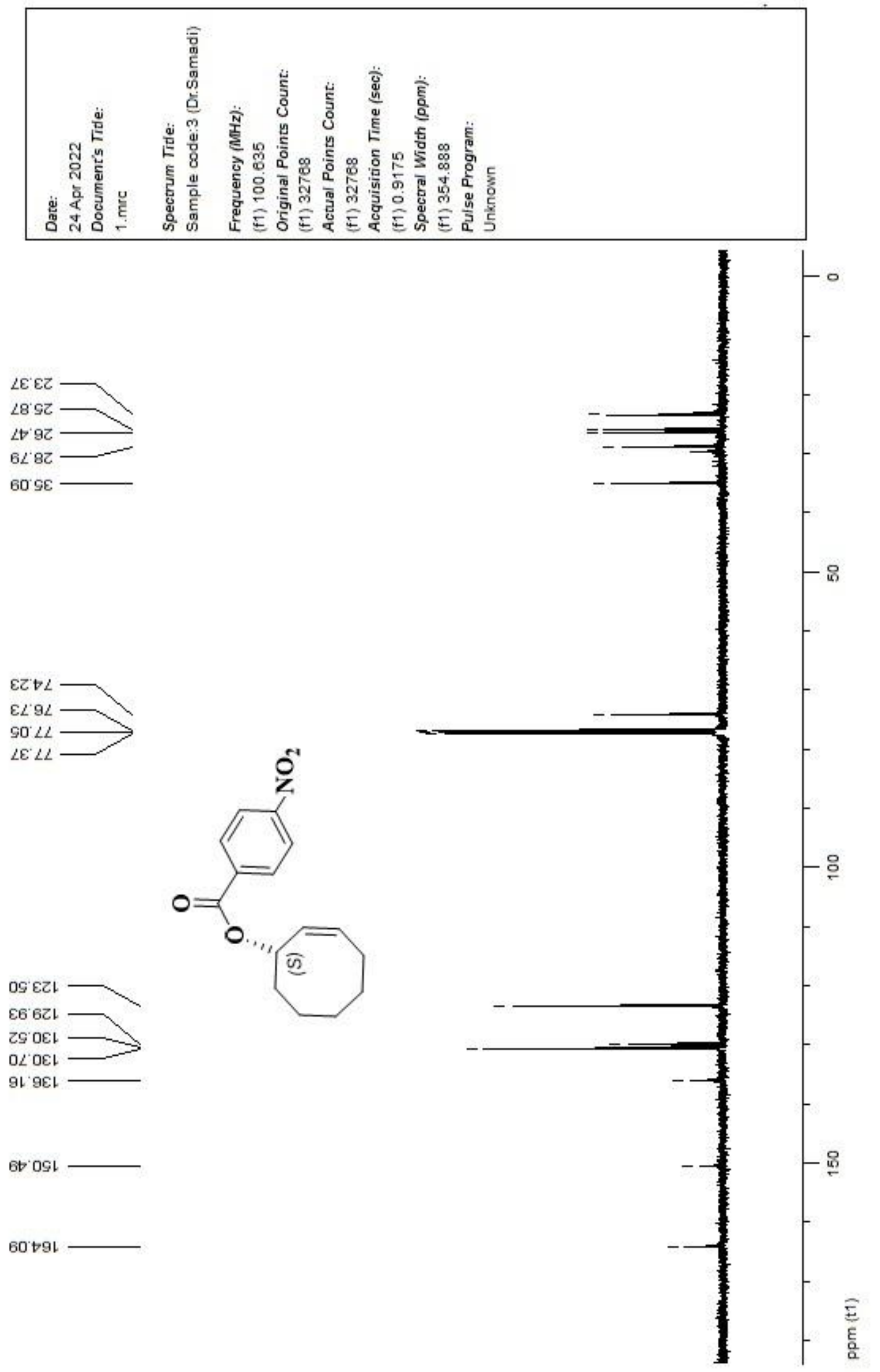

Figure S35: ^{13}CNMR of **10a**

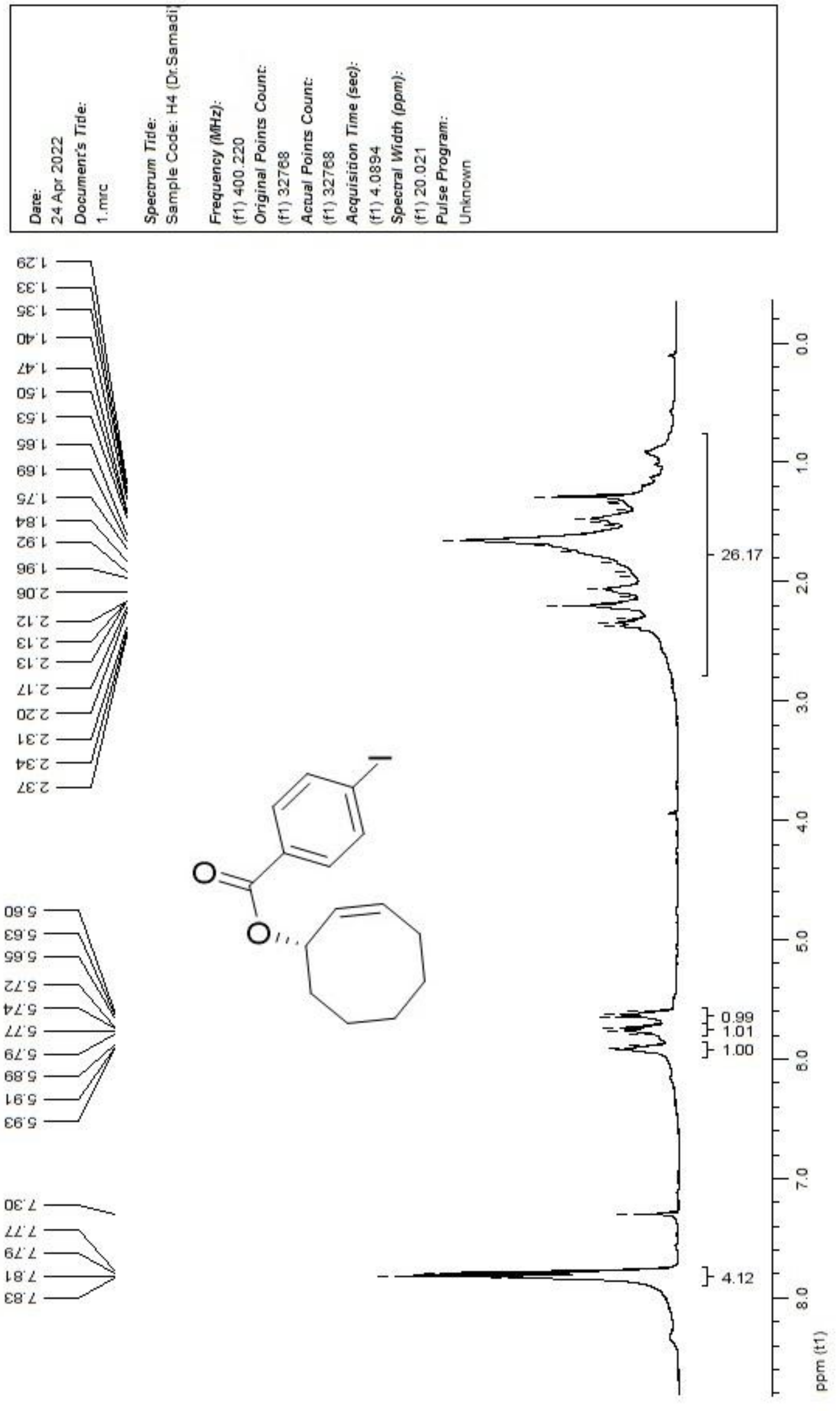

Figure S36: ^{1}HNMR of **10b**

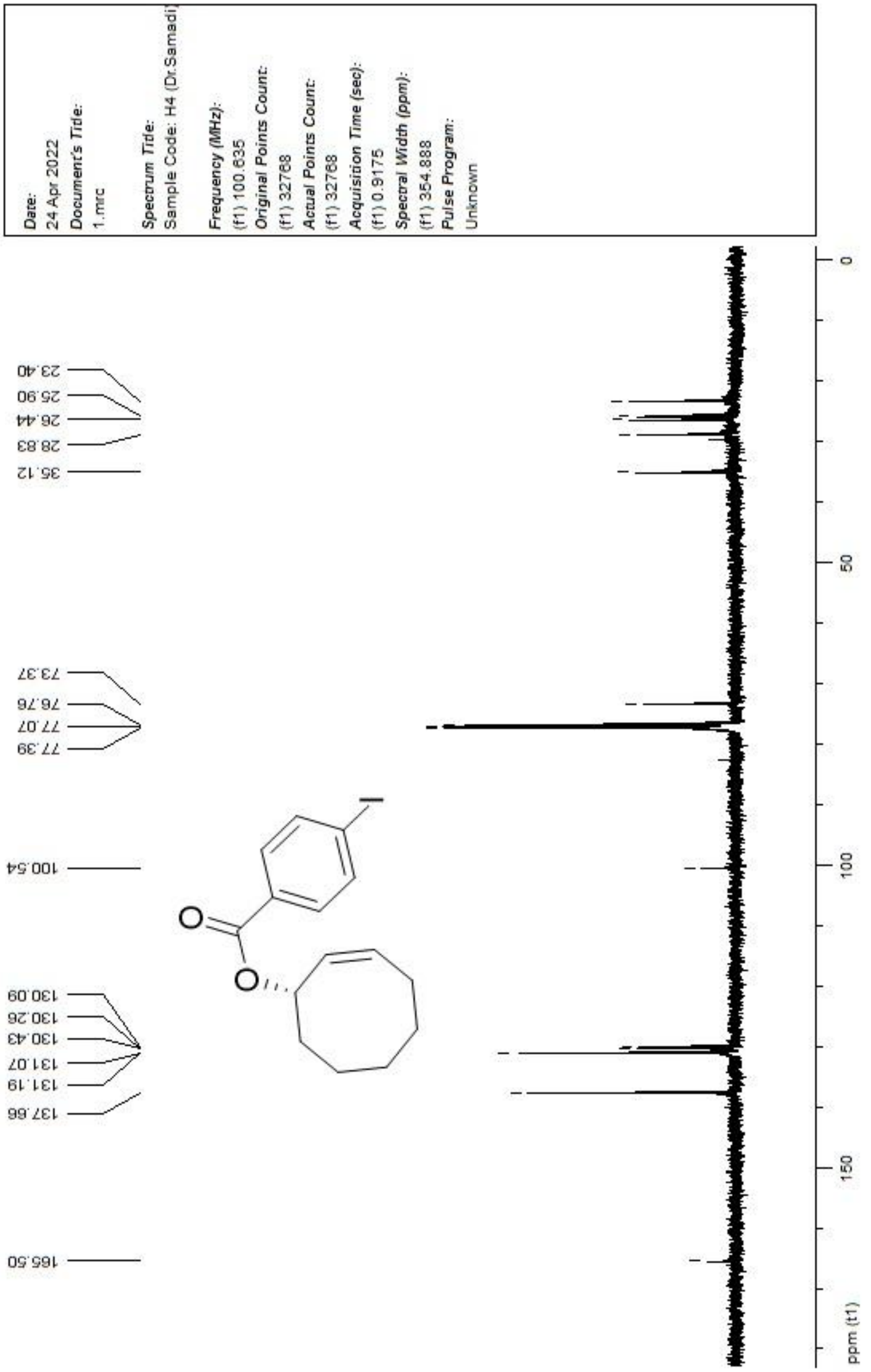

Figure 37: ^{13}CNMR of **10b**

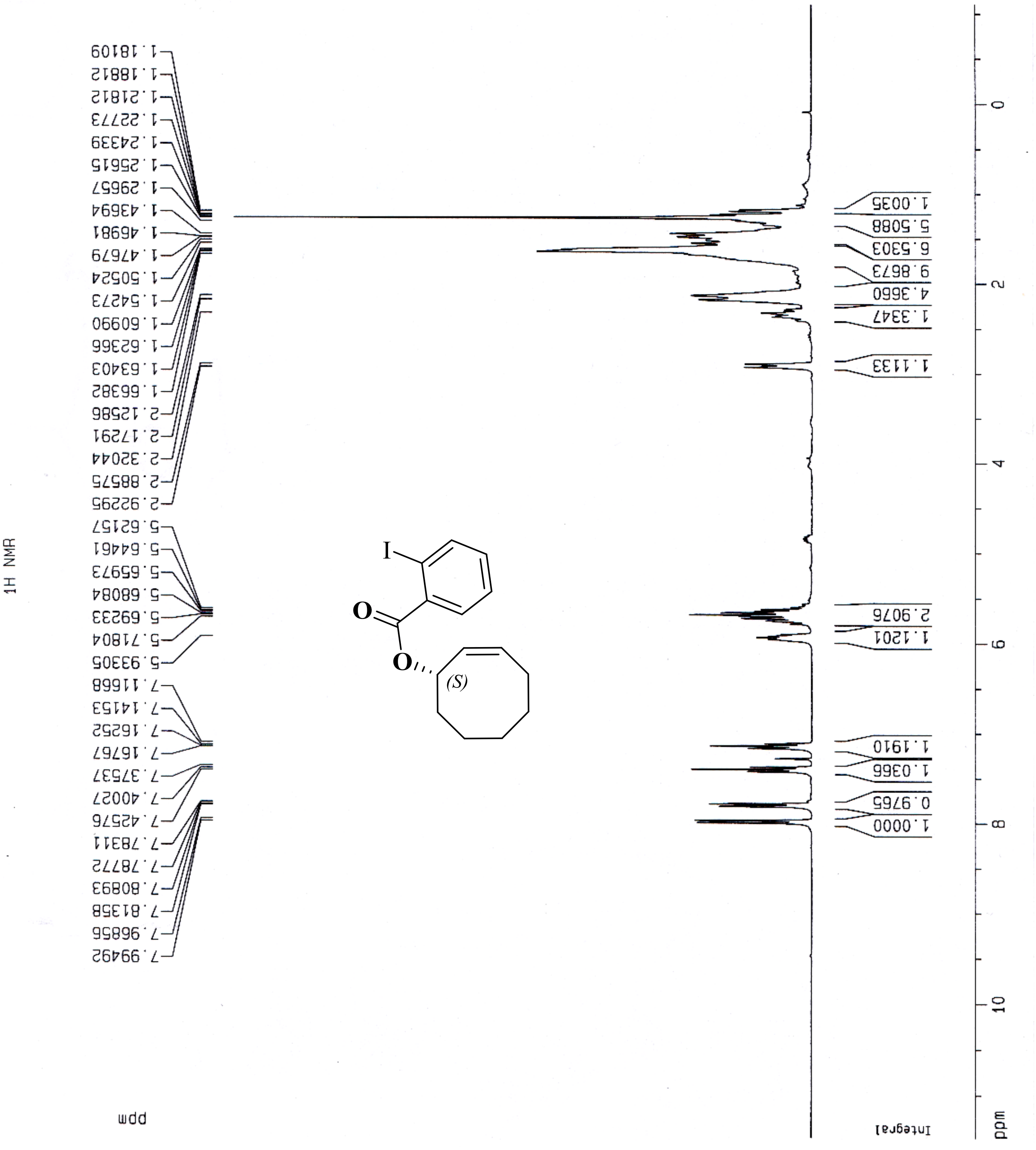

Figure S38: ^{1}HNMR of **10d**

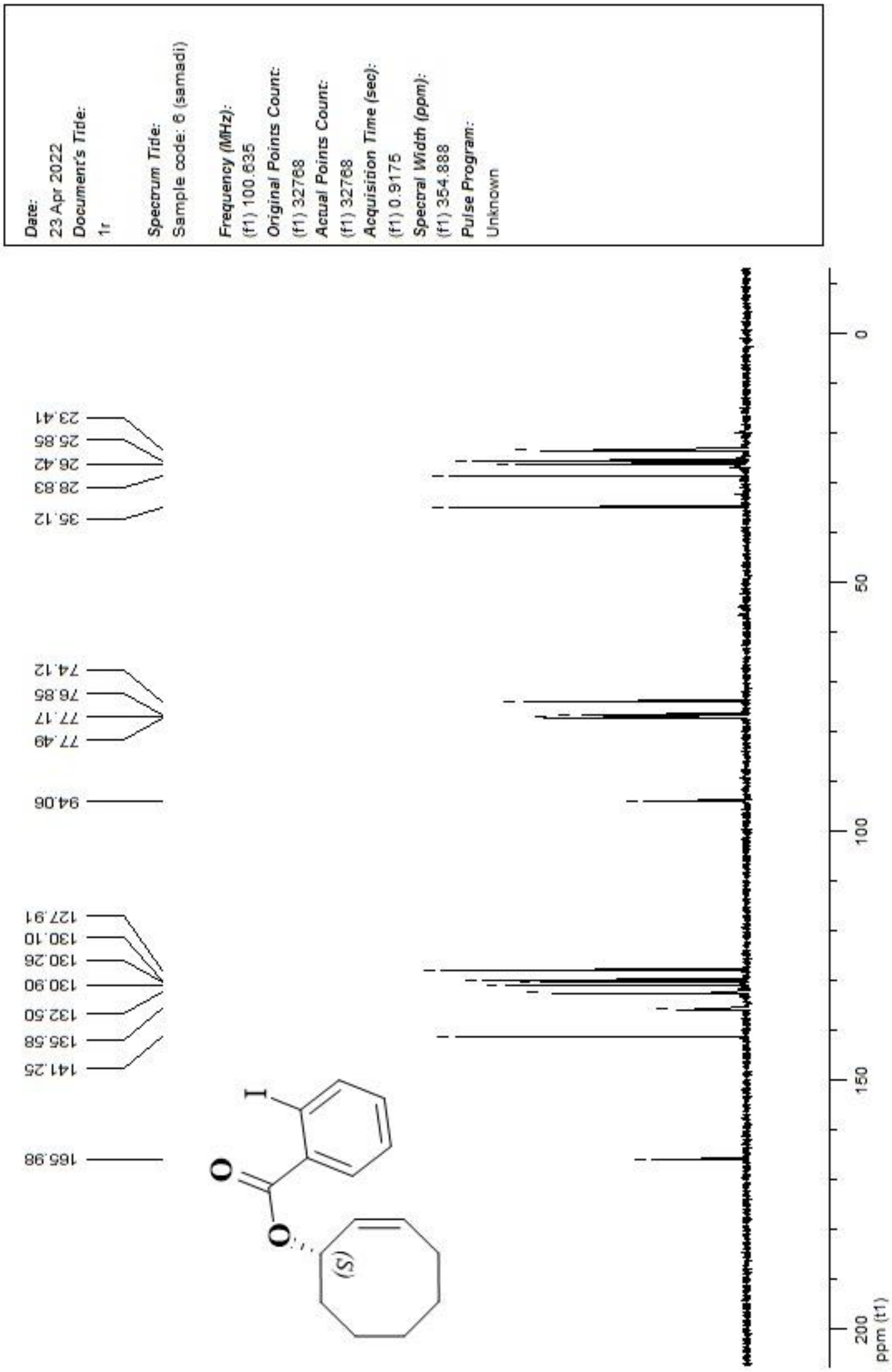

Figure S39: ^{13}CNMR of **10d**

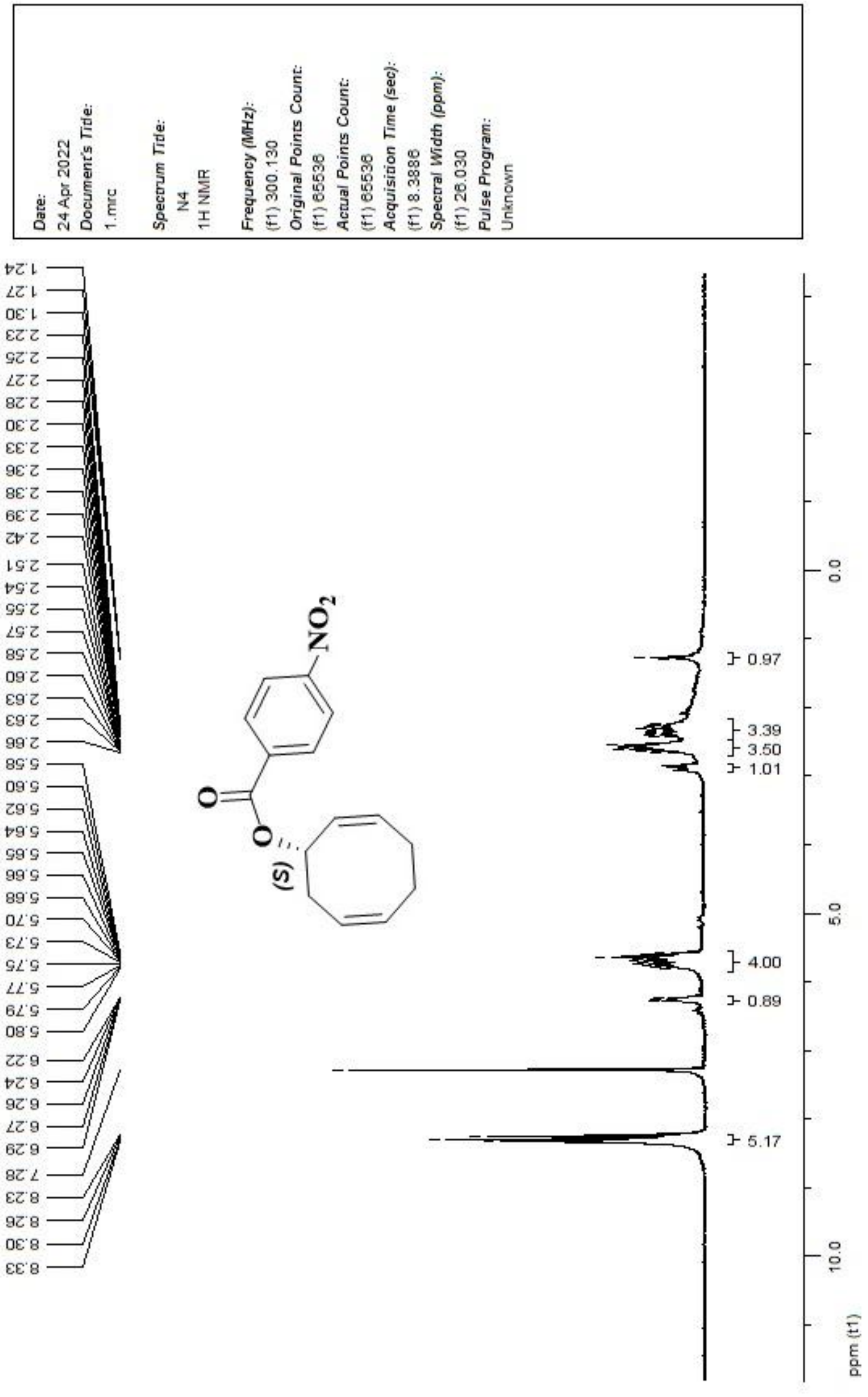

Figure S40: ^{1}HNMR of **11a**

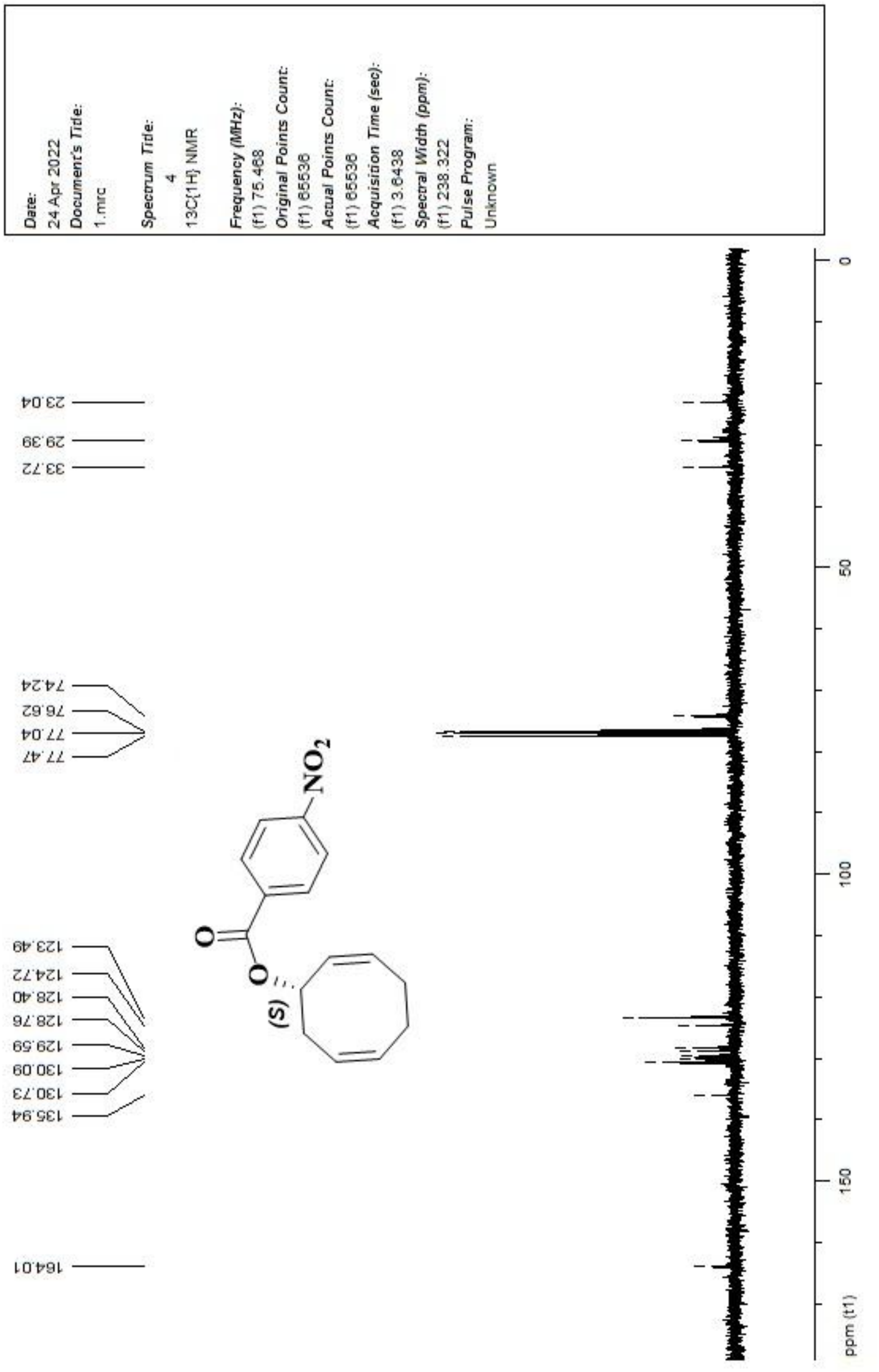

Figure S41: ^{13}CNMR of **11a**

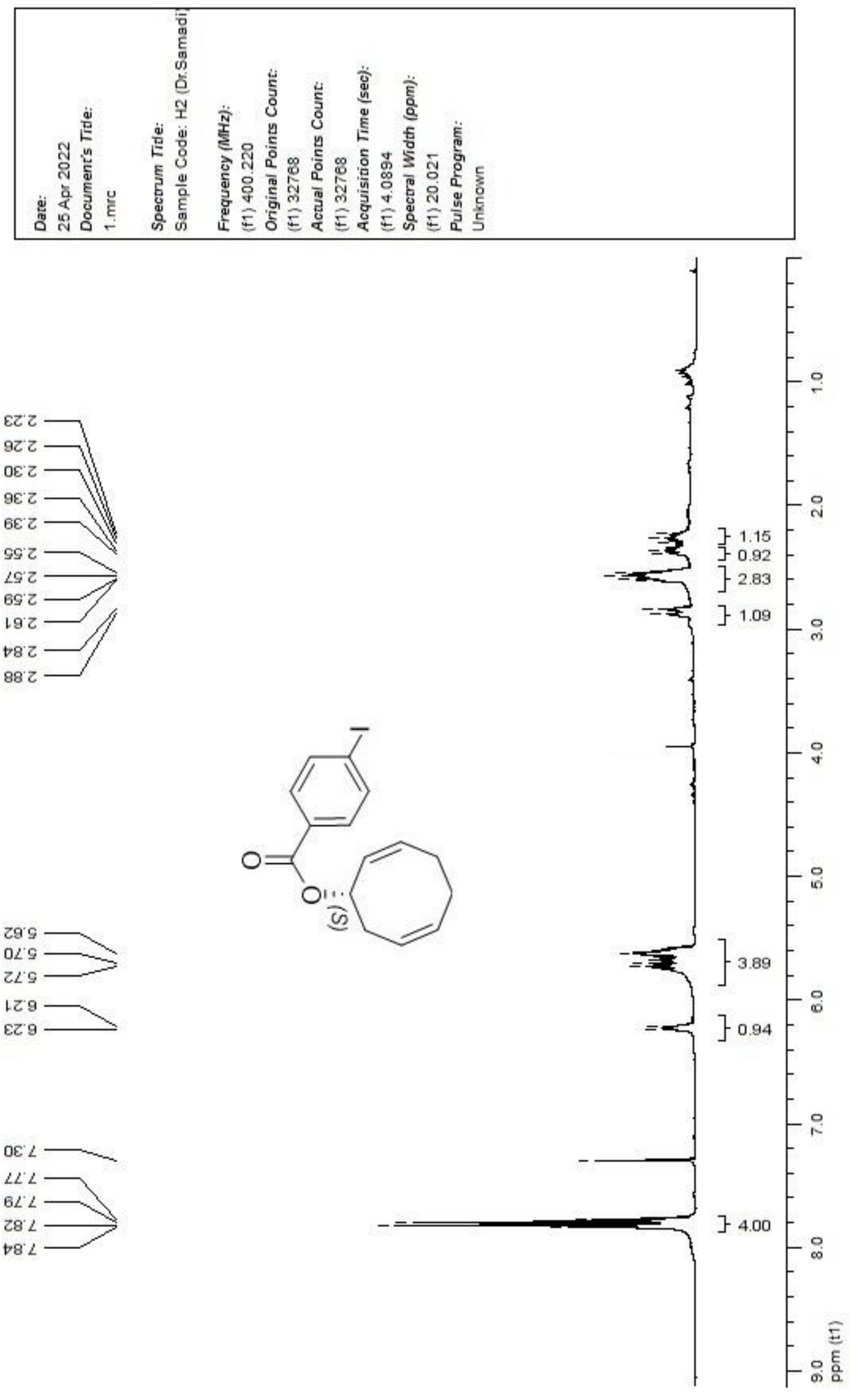

Figure S42: ^{1}HNMR of **11b**

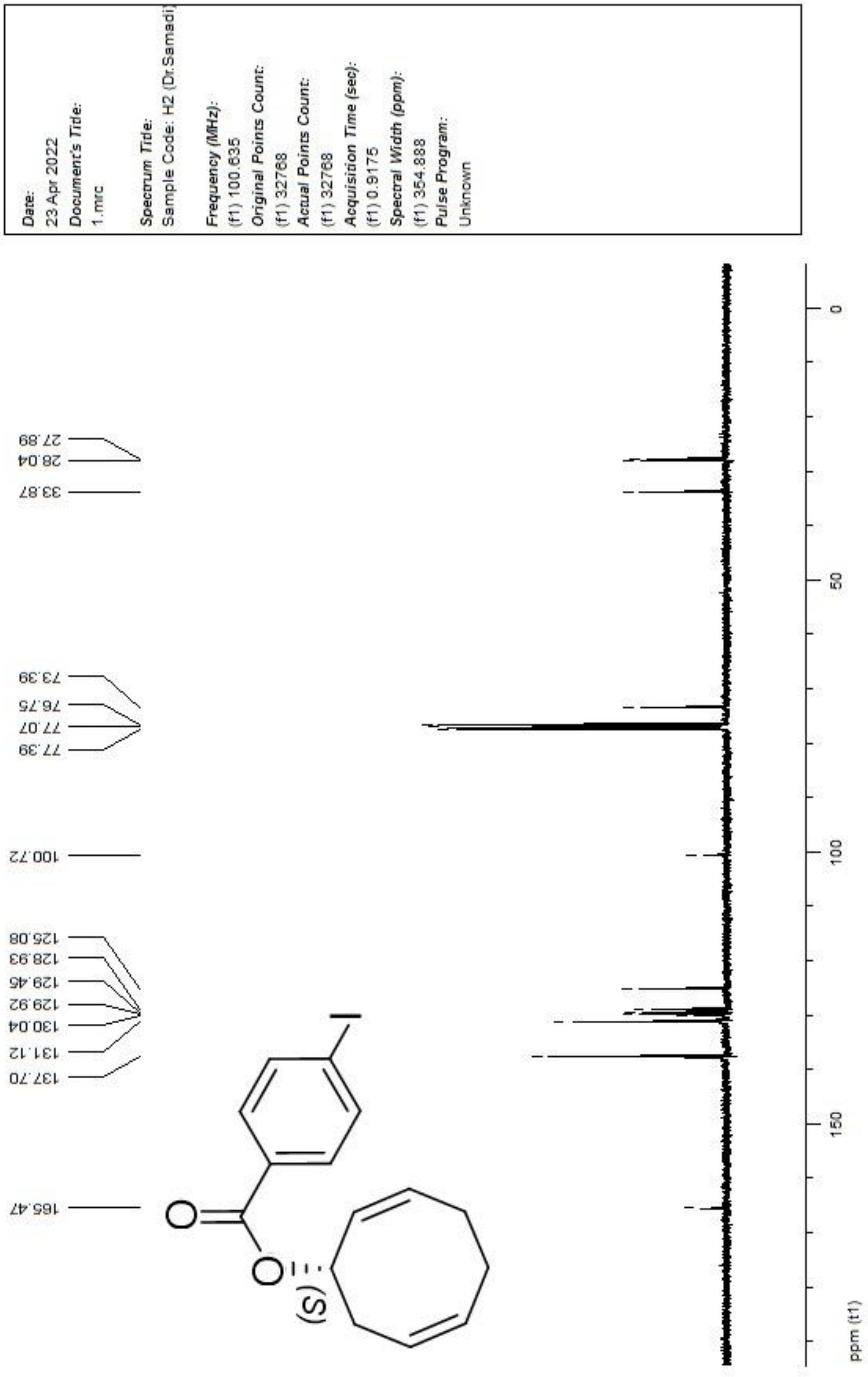

Figure S43: ^{13}CNMR of **11b**

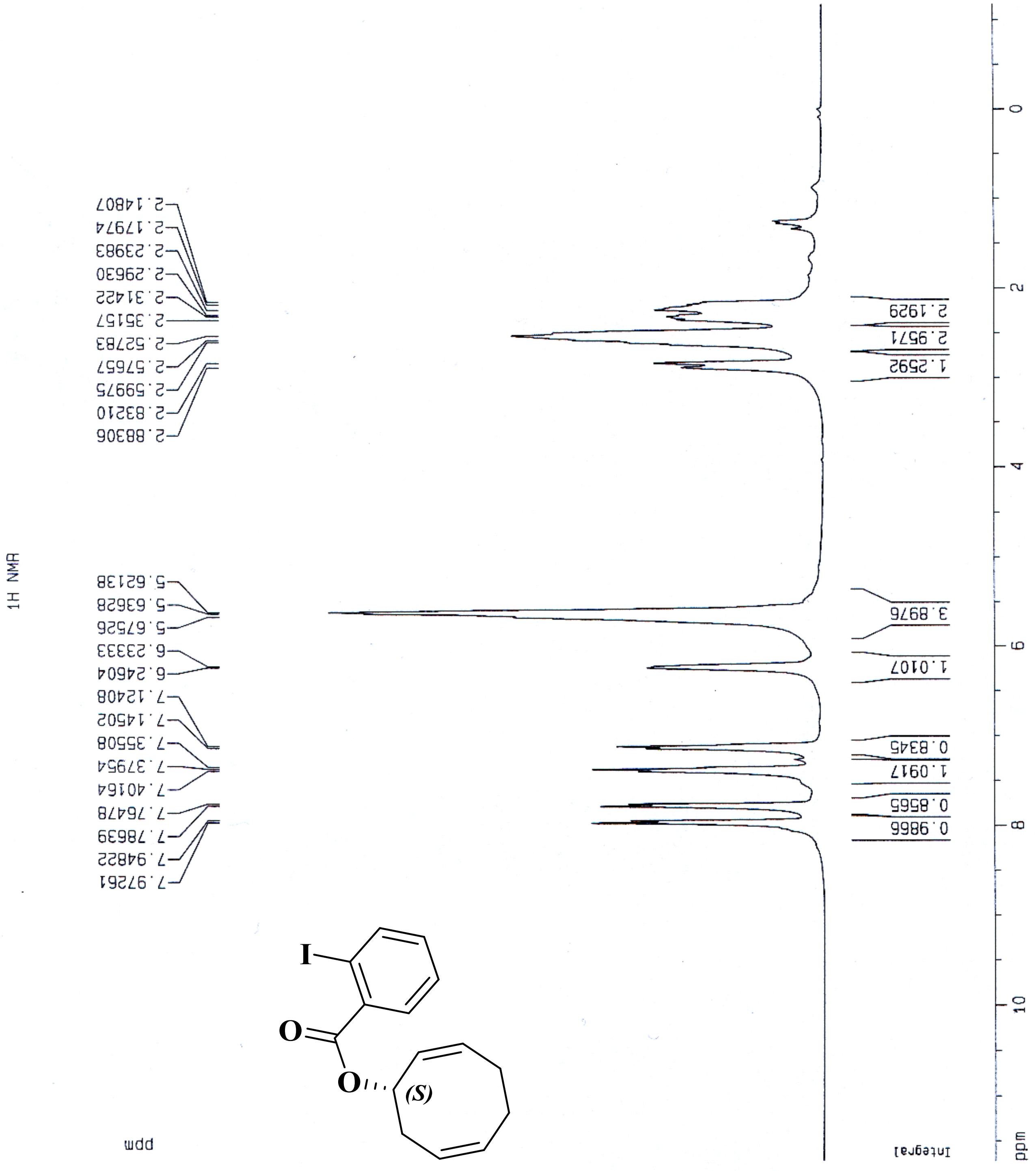

Figure S44: ^{1}HNMR of **11d**

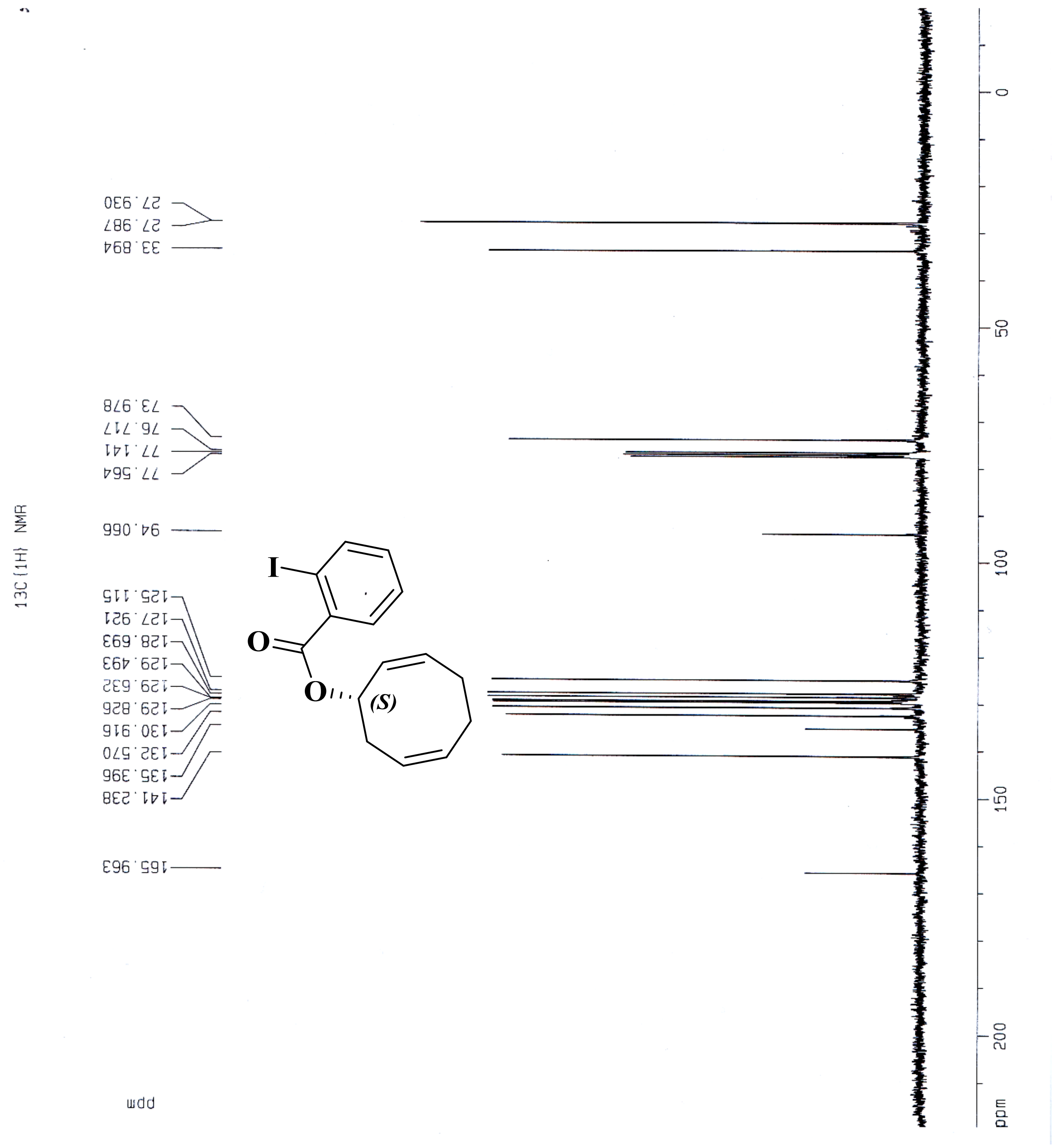

Figure S45: ^{13}CNMR of **11d**

Computational method:

In this study, the phenyl and *iso*propyl group substitutions were selected to examine the effect of aryl and alkyl groups on the isomeric complexes. First structures were drawn using Spartan software[19] and gauss view 6[20] and then Gaussian 9[21] and Gaussian 16 were employed for DFT calculations. Both frequency and optimization calculations were carried out with the B3LYP method at 6-31G(*d,p*) basis set level and CPCM as the method for chloroform solvent. The basis set was selected based on two factors: computational cost and an excellent agreement between computational and experimental results. In addition, long-range van der Waals interactions are taken into account using the Grimm's D3 dispersion correction for complexes (i.e. B3LYP-D3/6-31G (*d,p*) level of theory) to make the DFT energies of complexes more accurate. Different conformers of complexes with C_1 and C_2 symmetries were designed and optimized at the 6-31G (*d,p*) basis set level. The selection criterion for determining the stable conformers was the Gibbs free energy of the systems and not the symmetry of the molecules. All frequencies were done without imaginary frequency. The absence of imaginary frequencies in the computational outputs indicates that all optimized structures are at their minimum energy in the potential energy surfaces diagram. Moreover, Gibbs free energies were calculated to determine the thermodynamic stability of the complexes. In the case of intermediates containing 1-hexenyl groups, different conformers, including *cis* and *trans* forms of the 2-hexenyl group, were investigated to find global minimums. In addition, to determine the relative amounts of each isomer at equilibrium, equilibrium constant (*K*eq) at the 298.15 K, one atmosphere pressure and 1.98 x 10^{-3} kcal/(K.mole) as gas constant, were calculated [22].

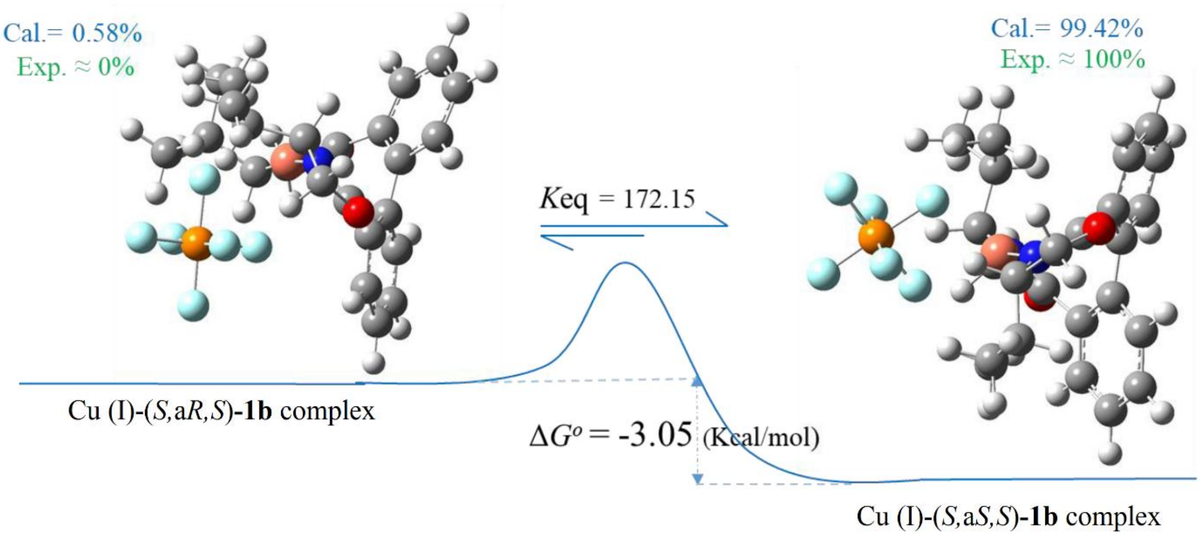

Figure S46. Gibbs free energy and equilibrium constant values for Cu (I)-**1b** complexes.

Geometry optimized coordinates of compounds

Cu (I)-(*S*,a*S*,*S*)-**1a** complex

C	-0.70969	0.23287	-2.69417
C	-1.66379	-0.5374	-3.36721
C	-2.96224	-0.06424	-3.56793
C	-3.31939	1.20934	-3.12897
C	-2.38504	1.99291	-2.45355
C	-1.09762	1.5025	-2.21174
C	0.70833	-0.23325	-2.69294
C	1.09679	-1.50374	-2.21232
C	2.3845	-1.99312	-2.4531
C	3.31998	-1.20973	-3.12941
C	2.96129	0.06531	-3.56864
C	1.66411	0.53668	-3.36695
H	-1.37293	-1.50632	-3.75985
H	-3.68494	-0.68545	-4.08766
H	-4.3214	1.5893	-3.30024
H	-2.65872	2.97226	-2.07468
H	2.65868	-2.97287	-2.07363
H	4.32257	-1.58799	-3.29967
H	3.68477	0.68598	-4.08858
H	1.37317	1.50747	-3.76066
C	0.75723	4.0663	-0.3829
C	1.11716	2.79795	0.43323
H	1.62304	4.60588	-0.76719
H	0.10674	4.75127	0.16561
H	0.76998	2.87768	1.46334
C	-0.75755	-4.0672	-0.38235
C	-1.11724	-2.79936	0.43289

H	-1.62372	-4.60586	-0.76715
H	-0.10621	-4.75087	0.16447
H	-0.77056	-2.87842	1.46346
O	0.00003	3.56574	-1.53175
O	-0.00031	-3.56551	-1.53189
N	0.30156	1.75067	-0.2363
C	-0.22061	2.25961	-1.29964
C	0.21999	-2.25952	-1.30009
N	-0.30197	-1.75169	-0.23756
Cu	0.00077	0.0003	0.30627
C	2.57726	2.3887	0.42239
C	3.18846	1.94943	1.60153
C	3.30123	2.36961	-0.77643
C	4.51582	1.51291	1.58172
H	2.6172	1.92957	2.52247
C	4.62401	1.92925	-0.79739
H	2.82585	2.68417	-1.70229
C	5.235	1.50264	0.38543
H	4.98269	1.17594	2.50269
H	5.17301	1.91273	-1.73315
H	6.26482	1.15938	0.37173
C	-2.57659	-2.38849	0.42313
C	-3.18943	-1.94954	1.60108
C	-3.30185	-2.3698	-0.77579
C	-4.51556	-1.51309	1.58295
H	-2.61775	-1.92849	2.52206
C	-4.62439	-1.92924	-0.79719
H	-2.82539	-2.68334	-1.70118
C	-5.23548	-1.50174	0.38494
H	-4.98401	-1.17484	2.50174

H	-5.17206	-1.91345	-1.73341
H	-6.26574	-1.15826	0.37271
P	0.00042	0.0002	3.43933
F	1.12429	-0.26814	2.23427
F	0.37953	1.59011	3.38829
F	1.13358	-0.26956	4.55681
F	-0.38099	-1.59026	3.38767
F	-1.13365	0.26916	4.55679
F	-1.12548	0.26748	2.23445

Sum of electronic and zero-point Energies= -3998.204772

Sum of electronic and thermal Energies= -3998.168526

Sum of electronic and thermal Enthalpies= -3998.167582

Sum of electronic and thermal Free Energies= -3998.278449

Cu (I)-(*S*,a*R*,*S*)-**1a** complex

C	-2.82003	0.57816	-0.66539
C	-2.20991	1.84591	-0.82916
C	-2.24428	2.50165	-2.06487
C	-2.84023	1.89455	-3.16841
C	-3.40419	0.62647	-3.03333
C	-3.4124	-0.01062	-1.79137
C	-3.11991	-0.01952	0.67547
C	-3.89242	0.7368	1.56974
C	-4.43619	0.16893	2.72219
C	-4.25753	-1.189	2.98644
C	-3.49474	-1.96267	2.11404
C	-2.90678	-1.38432	0.98199
H	-1.76429	3.46909	-2.16295

H	-2.85079	2.4027	-4.12681
H	-3.86754	0.14127	-3.88659
H	-3.9199	-0.96384	-1.68164
H	-4.1	1.77614	1.33551
H	-5.02687	0.78323	3.39454
H	-4.69947	-1.64189	3.86779
H	-3.31895	-3.01271	2.32285
C	-1.39103	2.42296	0.24835
C	0.0674	2.63613	1.94361
C	-0.42251	4.02916	1.46859
H	-0.40016	2.37849	2.90174
H	-0.8299	4.65134	2.26443
H	0.34892	4.57487	0.91895
N	-0.51611	1.74157	0.91339
O	-1.50397	3.72768	0.53299
C	1.57032	2.54361	2.09456
C	2.39976	2.30656	0.99127
C	2.14825	2.75355	3.35296
C	3.78603	2.27716	1.14841
H	1.96989	2.10847	0.01849
C	3.53564	2.73778	3.50606
H	1.51116	2.92876	4.21636
C	4.35821	2.49934	2.40273
H	4.40933	2.07173	0.28372
H	3.97172	2.90305	4.48685
H	5.43741	2.47828	2.52248
C	-1.92259	-2.18418	0.23249
C	0.03227	-2.89683	-0.64215
C	-1.0762	-3.96494	-0.8442
H	0.42708	-2.55105	-1.59777

H	-1.37315	-4.07583	-1.88883
H	-0.83249	-4.93912	-0.4209
N	-0.72717	-1.7728	-0.02922
O	-2.23351	-3.43982	-0.11799
C	1.18136	-3.34304	0.24574
C	0.93792	-3.89344	1.51271
C	2.50218	-3.21298	-0.19739
C	1.99587	-4.31324	2.31836
H	-0.08207	-3.99835	1.8743
C	3.56193	-3.63691	0.60823
H	2.69873	-2.76455	-1.1656
C	3.31219	-4.18819	1.86557
H	1.79362	-4.73854	3.29695
H	4.58262	-3.53216	0.25239
H	4.13666	-4.51691	2.4913
Cu	-0.23872	-0.00613	0.3135
P	1.77802	0.35549	-2.4693
F	0.92128	1.69397	-2.06331
F	1.90178	0.86305	-4.00135
F	3.15762	1.11631	-2.05946
F	2.60875	-1.00657	-2.81
F	1.63539	-0.16948	-0.88276
F	0.37557	-0.42219	-2.80515

Sum of electronic and zero-point Energies= -3998.201874

Sum of electronic and thermal Energies= -3998.165693

Sum of electronic and thermal Enthalpies= -3998.164749

Sum of electronic and thermal Free Energies= -3998.272900

Cu (I)-(*S*,a*S*,*S*)-**1b** complex

C	-2.71986	0.22882	-0.71286
C	-3.44583	1.3818	-1.03944
C	-3.65229	1.75784	-2.36768
C	-3.1584	0.96454	-3.40347
C	-2.43011	-0.18415	-3.10253
C	-2.18566	-0.54343	-1.76934
C	-2.7204	-0.22502	0.71243
C	-2.1851	0.54636	1.76902
C	-2.43044	0.18749	3.10215
C	-3.1607	-0.96001	3.40298
C	-3.65568	-1.75246	2.36708
C	-3.44834	-1.37678	1.03888
H	-3.8816	1.97145	-0.23885
H	-4.22056	2.65572	-2.5896
H	-3.33251	1.23993	-4.43853
H	-2.01958	-0.79723	-3.89769
H	-2.01905	0.79987	3.89741
H	-3.33544	-1.23509	4.43799
H	-4.22548	-2.64942	2.58885
H	-3.88496	-1.96572	0.23819
C	-0.32088	-3.66736	-1.91625
C	0.36664	-2.9859	-0.70785
H	-0.73184	-4.65343	-1.69649
H	0.31452	-3.72361	-2.80326
H	1.43554	-2.84111	-0.88586
C	-0.31492	3.66686	1.91714
C	0.37122	2.98492	0.70824
H	-0.72393	4.65392	1.69809

H	0.32063	3.72121	2.80413
H	1.43995	2.83842	0.88591
O	-1.44632	-2.79162	-2.23518
O	-1.44203	2.7931	2.23544
N	-0.28079	-1.64852	-0.68625
C	-1.27476	-1.67329	-1.51086
C	-1.27229	1.6747	1.51079
N	-0.27832	1.64854	0.68623
Cu	0.32574	-0.00034	-0.00016
P	3.47784	-0.00228	-0.00011
F	2.2704	-0.08422	1.15184
F	3.42507	-1.62845	-0.11442
F	4.595	-0.08513	1.16303
F	3.4285	1.62391	0.11416
F	4.5948	0.07817	-1.16368
F	2.27017	0.08212	-1.15174
C	0.18202	-3.73771	0.62951
H	-0.89345	-3.93893	0.74344
C	0.64653	-2.89178	1.82148
H	0.09137	-1.95226	1.87747
H	1.70932	-2.64708	1.73297
H	0.49479	-3.43668	2.75867
C	0.93193	-5.07807	0.59348
H	0.77087	-5.62959	1.52482
H	2.01028	-4.91478	0.4841
H	0.60515	-5.72224	-0.22951
C	0.1874	3.73764	-0.6287
H	-0.88784	3.94044	-0.74235
C	0.93916	5.07695	-0.59209
H	2.01735	4.91206	-0.48323

H	0.61364	5.721	0.23148
H	0.77849	5.62929	-1.52298
C	0.65056	2.89173	-1.82124
H	0.49965	3.43757	-2.75806
H	0.09391	1.95317	-1.87787
H	1.71296	2.64532	-1.73293

Sum of electronic and zero-point Energies= -3771.985026

Sum of electronic and thermal Energies= -3771.949690

Sum of electronic and thermal Enthalpies= -3771.948746

Sum of electronic and thermal Free Energies= -3772.054729

Cu (I)-(*S*,a*R*,*S*)-**1b** complex

H	-1.0402	-1.46303	5.01834
C	-1.36424	-1.12711	4.03887
C	-2.22288	-0.26571	1.48997
C	-1.98267	0.1136	3.88571
C	-1.14985	-1.93127	2.92121
C	-1.54769	-1.49835	1.65047
C	-2.42232	0.52811	2.62687
H	-2.15024	0.75047	4.7486
H	-0.63959	-2.88349	3.01937
H	-2.96063	1.46551	2.5266
C	-2.91851	0.12001	0.22243
C	-4.61662	0.93531	-1.88353
C	-3.95388	-0.70705	-0.23809
C	-2.73225	1.36511	-0.41722
C	-3.59467	1.7735	-1.44379
C	-4.78333	-0.31583	-1.28892
H	-4.12768	-1.6561	0.25941

H	-3.43696	2.7386	-1.91378
H	-5.57547	-0.97868	-1.62285
H	-5.27187	1.25506	-2.68716
N	-0.33627	1.81231	-0.17677
C	0.54683	2.98599	0.04364
C	-0.46323	4.11559	0.36415
O	-1.77469	3.51643	0.11939
C	-1.5606	2.2149	-0.12812
H	1.18216	2.77068	0.90542
H	-0.44082	4.42974	1.40938
H	-0.37767	4.99187	-0.2799
H	-1.19658	-4.90333	-1.12597
C	-0.58576	-4.12855	-0.66502
C	-0.36274	-2.88164	-1.54223
N	-0.55564	-1.77848	-0.56257
C	-1.13501	-2.28376	0.47576
O	-1.3151	-3.6158	0.49403
H	0.35199	-4.55889	-0.2979
H	-1.16619	-2.79398	-2.28846
Cu	0.08995	-0.01226	-0.41594
P	3.04766	-0.23591	0.82088
F	2.8231	-1.83995	0.63115
F	1.51017	-0.12704	1.44681
F	2.3643	-0.00285	-0.69011
F	3.1908	1.38216	0.97034
F	4.51095	-0.32959	0.14061
F	3.64729	-0.46014	2.30461
C	0.98829	-2.81411	-2.26874
H	1.76734	-2.76837	-1.49755
C	1.47057	3.23844	-1.16966

H	2.03275	2.3085	-1.29934
C	2.48128	4.34705	-0.8425
H	1.98955	5.31037	-0.66044
H	3.17559	4.48899	-1.67653
H	3.06901	4.0903	0.04437
C	0.70615	3.52208	-2.46923
H	0.1479	4.46469	-2.42438
H	-0.00033	2.71945	-2.70301
H	1.40605	3.6026	-3.30685
C	1.06378	-1.53606	-3.11289
H	2.06575	-1.39437	-3.52751
H	0.82542	-0.66759	-2.49308
H	0.34884	-1.56949	-3.94359
C	1.20998	-4.06332	-3.13373
H	2.16643	-3.99409	-3.66071
H	0.42119	-4.16468	-3.88874
H	1.22921	-4.98327	-2.53991

Sum of electronic and zero-point Energies= -3771.981671

Sum of electronic and thermal Energies= -3771.946384

Sum of electronic and thermal Enthalpies= -3771.945439

Sum of electronic and thermal Free Energies= -3772.049869

Calculations of the key reaction intermediate

Si-face intermediate containing cyclohexenyl group

Charge = 1 Multiplicity = 1

C	-1.19849	2.3561	-1.67995
C	-0.57409	3.6104	-1.73836
C	0.58745	3.80226	-2.48637
C	1.14497	2.73706	-3.19653
C	0.53863	1.48472	-3.14971
C	-0.62165	1.28494	-2.38669
C	-2.49387	2.24564	-0.94712
C	-2.54783	2.33185	0.45529
C	-3.77835	2.39035	1.12224
C	-4.96621	2.31507	0.39815
C	-4.92348	2.20636	-0.99349
C	-3.69726	2.18507	-1.65968
H	-1.01821	4.4419	-1.20093
H	1.05291	4.78191	-2.51564
H	2.04665	2.87944	-3.78256
H	0.96308	0.64861	-3.69447
H	-3.79949	2.47406	2.20336
H	-5.91817	2.34405	0.91715
H	-5.84508	2.15009	-1.56375
H	-3.66638	2.13439	-2.7434
C	-1.91762	-1.95518	-3.37742
C	-2.04929	-2.07665	-1.83478
H	-2.88381	-1.95912	-3.88548
H	-1.25837	-2.70345	-3.81791
H	-1.35161	-2.83282	-1.46123
C	0.13958	3.01109	2.82344
C	0.62032	1.65262	2.23398

H	0.85903	3.81799	2.68811
H	-0.1648	2.94363	3.86908
H	0.65781	0.88739	3.01124
O	-1.31762	-0.64509	-3.57858
O	-1.05106	3.34361	2.03979
N	-1.57796	-0.75462	-1.344
C	-1.20622	-0.06866	-2.37013
C	-1.30695	2.29621	1.2546
N	-0.48483	1.30075	1.30281
C	-3.45175	-2.40658	-1.36695
C	-3.78739	-3.71537	-1.00369
C	-4.43158	-1.40732	-1.31112
C	-5.08409	-4.02008	-0.58292
H	-3.03255	-4.49657	-1.04264
C	-5.72405	-1.70877	-0.88295
H	-4.17273	-0.38927	-1.57768
C	-6.05354	-3.01714	-0.5176
H	-5.33262	-5.03807	-0.29899
H	-6.46901	-0.92053	-0.82914
H	-7.05845	-3.25242	-0.18073
C	1.98062	1.73489	1.57188
C	3.12605	1.49795	2.34047
C	2.11995	2.09713	0.23043
C	4.39483	1.63699	1.77681
H	3.02439	1.19167	3.37798
C	3.38786	2.23309	-0.33486
H	1.238	2.25267	-0.37453
C	4.5291	2.00764	0.43695
H	5.27641	1.44054	2.37914
H	3.47812	2.50314	-1.38235
H	5.51561	2.10094	-0.0065

Cu	-0.8129	-0.52204	0.59711
O	0.93109	-0.98419	-0.09504
C	1.82219	-1.29298	0.78054
C	3.08035	-1.91033	0.22683
C	3.27396	-2.01514	-1.15625
C	4.08143	-2.3411	1.10594
C	4.4569	-2.5396	-1.66391
H	2.49806	-1.66367	-1.82434
C	5.27002	-2.86994	0.6176
H	3.91798	-2.24218	2.17212
C	5.43908	-2.95656	-0.76512
H	4.62938	-2.61983	-2.72911
H	6.05749	-3.20306	1.28075
O	1.71854	-1.11278	2.01018
C	-2.49352	-0.98285	1.64861
C	-1.6929	-2.16601	1.54824
C	-2.65256	-0.31747	3.00208
H	-3.30373	-0.8448	0.93879
C	-0.76049	-2.44348	2.48771
H	-1.83072	-2.85908	0.72731
C	-2.15031	-1.20886	4.14634
H	-2.12259	0.63764	3.03109
H	-3.71148	-0.07616	3.13527
C	-0.76181	-1.78567	3.83939
H	-0.07371	-3.26246	2.31556
H	-2.12481	-0.64083	5.07967
H	-2.85099	-2.03944	4.28738
H	-0.45265	-2.51793	4.59292
H	0.0139	-1.01106	3.85203
N	6.69626	-3.4991	-1.29186
O	7.55694	-3.86317	-0.4866
O	6.8356	-3.56513	-2.51584

Sum of electronic and zero-point Energies= -3916.071691

Sum of electronic and thermal Energies= -3916.026279

Sum of electronic and thermal Enthalpies= -3916.025335

Sum of electronic and thermal Free Energies= -3916.154510

Re-face intermediate containing cyclohexenyl group

Charge = 1 Multiplicity = 1

C	-1.37943	2.65163	-1.53227
C	-0.95464	3.9885	-1.5241
C	0.1627	4.39671	-2.25165
C	0.86999	3.47249	-3.02255
C	0.47115	2.13944	-3.03434
C	-0.63293	1.71761	-2.27457
C	-2.64894	2.32638	-0.81925
C	-2.72669	2.43105	0.58073
C	-3.9575	2.33574	1.23963
C	-5.11594	2.07302	0.50883
C	-5.0431	1.9242	-0.87795
C	-3.82143	2.06779	-1.53851
H	-1.52295	4.71332	-0.95056
H	0.47077	5.43676	-2.22518
H	1.73301	3.78392	-3.60108
H	1.02788	1.40589	-3.60599
H	-4.00178	2.44368	2.31879
H	-6.06828	1.98364	1.02027
H	-5.94096	1.71484	-1.45032
H	-3.77416	1.99639	-2.62043
C	-0.85967	-1.77823	-3.18021
C	-1.29997	-1.86734	-1.69514
H	-1.58316	-2.20171	-3.87659
H	0.13007	-2.2039	-3.35757

H	-0.58544	-2.46936	-1.12879
C	0.06422	3.47879	2.69869
C	0.52019	2.04817	2.28469
H	0.72603	4.26096	2.32489
H	-0.09058	3.5936	3.77165
H	0.56865	1.39223	3.1576
O	-0.77857	-0.34748	-3.42996
O	-1.23305	3.65335	2.04058
N	-1.17504	-0.46425	-1.21976
C	-0.90695	0.27265	-2.24682
C	-1.47246	2.53216	1.35738
N	-0.60085	1.58501	1.42621
C	-2.69441	-2.43098	-1.50899
C	-2.87758	-3.81956	-1.56341
C	-3.80294	-1.60347	-1.3104
C	-4.15232	-4.37058	-1.41954
H	-2.01983	-4.47084	-1.70954
C	-5.07791	-2.15537	-1.15586
H	-3.66817	-0.53013	-1.25884
C	-5.25615	-3.53868	-1.20854
H	-4.28113	-5.44777	-1.45893
H	-5.9274	-1.49925	-0.99051
H	-6.24558	-3.96749	-1.08449
C	1.86247	2.00954	1.5819
C	3.02693	1.78414	2.32449
C	1.96351	2.23201	0.20574
C	4.2737	1.78449	1.69869
H	2.95555	1.58818	3.3902
C	3.2089	2.21905	-0.42386
H	1.06668	2.4021	-0.3733
C	4.36805	1.99773	0.32126

H	5.16929	1.597	2.2828
H	3.26604	2.37759	-1.49628
H	5.33678	1.97547	-0.16828
Cu	-0.76952	-0.35115	0.76763
O	0.84806	-1.12129	0.14662
C	1.25964	-2.30171	0.50449
C	2.76343	-2.42345	0.57792
C	3.57858	-1.28567	0.51973
C	3.33749	-3.68488	0.77648
C	4.95615	-1.3992	0.66899
H	3.11955	-0.31526	0.37623
C	4.71315	-3.81932	0.91797
H	2.68804	-4.54992	0.83478
C	5.50129	-2.66829	0.8676
H	5.60244	-0.53174	0.64061
H	5.17574	-4.78483	1.07526
O	0.54765	-3.26968	0.79092
C	-1.30031	-3.0097	2.31085
C	-1.13571	-1.67252	2.28593
C	-2.67882	-3.64641	2.16225
H	-0.57454	-3.57197	2.8881
C	-2.31867	-0.76603	2.04125
H	-0.19881	-1.19974	2.54692
C	-3.76609	-2.6971	2.68878
H	-2.87566	-3.85281	1.10559
H	-2.6886	-4.61416	2.66576
C	-3.66205	-1.32811	2.00007
H	-2.1646	0.30676	1.97521
H	-4.75311	-3.13462	2.51576
H	-3.65177	-2.56337	3.77087
H	-4.3395	-0.58953	2.44135

H	-3.96312	-1.4036	0.94385
N	6.95288	-2.79674	1.0356
O	7.63759	-1.77117	0.99769
O	7.42242	-3.92434	1.20845

Sum of electronic and zero-point Energies= -3916.068038

Sum of electronic and thermal Energies= -3916.022890

Sum of electronic and thermal Enthalpies= -3916.021946

Sum of electronic and thermal Free Energies= -3916.149630

Si-face intermediate containing 1-hexenyl group

Charge = 1 Multiplicity = 1

C	1.09574	-1.83395	-1.94442
C	0.37106	-3.01146	-2.16459
C	-0.93385	-2.96844	-2.65665
C	-1.53017	-1.74329	-2.96057
C	-0.8193	-0.56328	-2.76107
C	0.47896	-0.60453	-2.23669
C	2.52519	-1.92113	-1.52293
C	2.90874	-2.34037	-0.23522
C	4.26184	-2.52644	0.07974
C	5.24525	-2.26961	-0.87281
C	4.87563	-1.84495	-2.15065
C	3.52762	-1.67752	-2.47111
H	0.84408	-3.96648	-1.95846
H	-1.48024	-3.89343	-2.8102
H	-2.54488	-1.70474	-3.34167
H	-1.27631	0.39635	-2.97605
H	4.53407	-2.85925	1.07582
H	6.29159	-2.40399	-0.6203
H	5.63466	-1.64738	-2.90051
H	3.24055	-1.367	-3.47056

C	1.78222	2.78874	-2.38419
C	2.12602	2.42532	-0.91251
H	2.65684	3.04376	-2.983
H	1.02423	3.56865	-2.46322
H	1.53819	3.05165	-0.23844
C	0.95734	-3.76967	2.48049
C	0.20945	-2.42149	2.2897
H	0.31882	-4.6425	2.34465
H	1.48525	-3.83434	3.43322
H	0.27743	-1.82511	3.20099
O	1.21158	1.56149	-2.93342
O	1.95719	-3.7808	1.41721
N	1.60474	1.04146	-0.78714
C	1.14321	0.67559	-1.93621
C	1.90634	-2.58205	0.81967
N	1.01662	-1.74632	1.23299
C	3.59707	2.53206	-0.56837
C	4.09405	3.71456	-0.00652
C	4.47223	1.46693	-0.80945
C	5.45116	3.83199	0.30122
H	3.4177	4.54089	0.19583
C	5.82578	1.58046	-0.49366
H	4.08849	0.54316	-1.2232
C	6.3197	2.76461	0.05982
H	5.82675	4.75235	0.73795
H	6.48948	0.74045	-0.67574
H	7.3729	2.85308	0.30838
C	-1.25112	-2.55586	1.91145
C	-2.23948	-1.94935	2.69236
C	-1.63065	-3.31352	0.79735
C	-3.59006	-2.10476	2.37257

H	-1.95269	-1.358	3.55743
C	-2.97843	-3.47026	0.47414
H	-0.87156	-3.77884	0.17751
C	-3.96264	-2.87146	1.26657
H	-4.34812	-1.62828	2.9865
H	-3.25932	-4.05628	-0.39548
H	-5.01216	-2.99475	1.01741
Cu	0.92493	0.31673	0.95209
O	-0.91469	0.3526	0.49198
C	-1.42643	1.46108	0.05486
C	-2.85138	1.33177	-0.41529
C	-3.46133	0.07317	-0.49343
C	-3.56249	2.48134	-0.78235
C	-4.77492	-0.04383	-0.9323
H	-2.89934	-0.80505	-0.20817
C	-4.87831	2.38257	-1.2207
H	-3.07084	3.44487	-0.7183
C	-5.46285	1.11653	-1.28883
H	-5.26255	-1.00771	-0.9955
H	-5.44806	3.25707	-1.50629
O	-0.85253	2.55963	0.01755
C	2.55676	0.73266	2.06672
H	3.26038	-0.08522	2.17593
C	-0.89796	1.71609	3.32292
H	-0.80435	1.33579	4.34887
H	-1.47699	0.95301	2.78154
N	-6.85192	1.00252	-1.75104
O	-7.35568	-0.1217	-1.80776
O	-7.44946	2.03594	-2.0605
C	1.44588	0.81323	2.93194
H	1.2549	0.01743	3.64581

C	0.45178	1.75845	2.69066
H	0.68606	2.62408	2.07232
H	2.94754	1.62644	1.59004
C	-1.65566	3.04927	3.27976
H	-1.12908	3.78822	3.8953
H	-1.64354	3.42155	2.24981
C	-3.10067	2.89382	3.75829
H	-3.14225	2.51127	4.78418
H	-3.62854	3.85177	3.7353
H	-3.64731	2.19241	3.11746

Sum of electronic and zero-point Energies= -3917.265340

Sum of electronic and thermal Energies= -3917.218239

Sum of electronic and thermal Enthalpies= -3917.217295

Sum of electronic and thermal Free Energies= -3917.350653

Re-face intermediate containing 1-hexenyl group

Charge = 1 Multiplicity = 1

C	1.28965	-2.66811	-1.13779
C	0.61343	-3.89211	-1.03772
C	-0.53725	-4.14192	-1.78493
C	-1.03054	-3.16816	-2.65543
C	-0.37363	-1.94579	-2.76566
C	0.77505	-1.68509	-2.00347
C	2.57917	-2.51288	-0.40208
C	2.62021	-2.37712	0.99699
C	3.8422	-2.39793	1.68272
C	5.03681	-2.51128	0.97525
C	5.00805	-2.63018	-0.41608
C	3.78896	-2.64145	-1.09547
H	1.0089	-4.65624	-0.37655

H	-1.04409	-5.09646	-1.68935
H	-1.92234	-3.35754	-3.24326
H	-0.74808	-1.17977	-3.43575
H	3.84866	-2.30319	2.76343
H	5.98249	-2.51073	1.50638
H	5.93416	-2.72406	-0.97378
H	3.76726	-2.76484	-2.17341
C	2.21005	1.34262	-3.39458
C	2.33779	1.65819	-1.87749
H	3.176	1.26898	-3.89679
H	1.56082	2.03749	-3.9282
H	1.68986	2.50164	-1.61937
C	-0.01668	-2.53407	3.49375
C	-0.54863	-1.3703	2.61524
H	-0.73078	-3.34644	3.62088
H	0.36152	-2.20259	4.4629
H	-0.65042	-0.455	3.19995
O	1.59062	0.02647	-3.4298
O	1.12281	-3.05392	2.73727
N	1.78137	0.44488	-1.22477
C	1.41476	-0.36574	-2.15755
C	1.3845	-2.16778	1.7754
N	0.56377	-1.17682	1.64397
C	3.75531	1.96665	-1.43814
C	4.2143	3.29001	-1.43942
C	4.62475	0.94102	-1.05068
C	5.52486	3.58155	-1.05578
H	3.54338	4.09358	-1.73224
C	5.93207	1.23276	-0.6598
H	4.27165	-0.0826	-1.04237
C	6.38578	2.55407	-0.66193

H	5.86934	4.61113	-1.05651
H	6.59081	0.42665	-0.35075
H	7.40163	2.78282	-0.35473
C	-1.88525	-1.67172	1.96148
C	-3.04627	-1.56239	2.73942
C	-1.99273	-2.07447	0.63083
C	-4.29293	-1.86109	2.19345
H	-2.97348	-1.22911	3.77143
C	-3.24285	-2.37196	0.08198
H	-1.1057	-2.12978	0.01733
C	-4.39484	-2.26872	0.85933
H	-5.18589	-1.76418	2.80363
H	-3.30854	-2.67067	-0.95905
H	-5.36704	-2.48804	0.42877
Cu	0.92141	0.55632	0.71471
O	-0.75758	0.93048	-0.10429
C	-1.77564	1.16501	0.66482
C	-3.11092	1.10843	-0.02927
C	-3.20535	0.74749	-1.37997
C	-4.2723	1.36636	0.70777
C	-4.44703	0.6369	-1.9935
H	-2.29914	0.53623	-1.93345
C	-5.52334	1.26092	0.1111
H	-4.17902	1.63107	1.75362
C	-5.58919	0.89307	-1.23303
H	-4.542	0.35093	-3.03276
H	-6.43337	1.44781	0.66582
O	-1.71577	1.41607	1.87719
C	2.56033	0.89696	1.82559
H	3.47973	0.5207	1.39088
C	0.18689	3.84208	1.33939

H	0.13085	4.58994	2.14364
H	-0.85388	3.58622	1.1026
N	-6.90561	0.76407	-1.86804
O	-6.94985	0.43666	-3.05654
O	-7.90746	0.98799	-1.18409
C	2.09207	2.17017	1.43874
H	2.60297	2.70602	0.64621
C	0.87172	2.6457	1.90337
H	0.4177	2.17651	2.77198
H	2.28769	0.49315	2.79716
C	0.91045	4.45493	0.13217
H	1.04289	5.52532	0.3216
H	1.91036	4.01354	0.04596
C	0.1301	4.24326	-1.16656
H	-0.86066	4.70782	-1.10675
H	0.65756	4.68429	-2.01765
H	-0.01314	3.17732	-1.37567

Sum of electronic and zero-point Energies= -3917.264508

Sum of electronic and thermal Energies= -3917.217741

Sum of electronic and thermal Enthalpies= -3917.216797

Sum of electronic and thermal Free Energies= -3917.348459

References:

1. McKennon, M. J., Meyers, A., Drauz, K. & Schwarm, M. A convenient reduction of amino acids and their derivatives. *J. Org. Chem.* **58**, 3568-3571 (1993).

2. Samadi, S., Ashouri, A., Rashid, H. I., Majidian, S. & Mahramasrar, M. Immobilization of (L)-valine and (L)-valinol on SBA-15 nanoporous silica and their application as chiral heterogeneous ligands in the Cu-catalyzed asymmetric allylic oxidation of alkenes. *New J. Chem.* **45**, 17630-17641 (2021).

3. Samadi, S., Ashouri, A. & Samadi, M. Synthesis of chiral allylic esters by using the new recyclable chiral heterogeneous oxazoline-based catalysts. *ACS Omega* **5**, 22367-22378 (2020).

4. Samadi, S., Jadidi, K., Khanmohammadi, B. & Tavakoli, N. Heterogenization of chiral mono oxazoline ligands by grafting onto mesoporous silica MCM-41 and their application in copper-catalyzed asymmetric allylic oxidation of cyclic olefins. *J. Catal.* **340**, 344-353 (2016).

5. Samadi, S., Jadidi, K. & Notash, B. Chiral bisoxazoline ligands with a biphenyl backbone: development and application in catalytic asymmetric allylic oxidation of cycloolefins. *Tetrahedron: Asymmetry* **24**, 269-277 (2013).

6. Samadi, S., Jadidi, K., Samadi, M., Ashouri, A. & Notash, B. Designing chiral amido-oxazolines as new chelating ligands devoted to direct Cu-catalyzed oxidation of allylic C-H bonds in cyclic olefins. *Tetrahedron* **75**, 862-867 (2019).

7. Samadi, S., Nazari, S., Arvinnezhad, H., Jadidi, K. & Notash, B. A significant improvement in enantioselectivity, yield, and reactivity for the copper-bi-*o*-tolyl bisoxazoline-catalyzed asymmetric allylic oxidation of cyclic olefins using recoverable SBA-15 mesoporous silica material. *Tetrahedron* **69**, 6679-6686 (2013).

8. Imai, Y., Zhang, W., Kida, T., Nakatsuji, Y. & Ikeda, I. Novel chiral bisoxazoline ligands with a biphenyl backbone: preparation, complexation, and application in asymmetric catalytic reactions. *J. Org. Chem.* **65**, 3326-3333 (2000).

9. Faraji, L., Samadi, S., Jadidi, K. & Notash, B. Synthesis of novel chiral diamino alcohols and their application in copper-catalyzed asymmetric allylic oxidation of cycloolefins. *Bull. Korean Chem. Soc.* **35**, 1989-1995 (2014).

10. Sadjadi, S., Samadi, S. & Samadi, M. $Cu(CH_3CN)_4PF_6$ immobilized on halloysite as efficient heterogeneous catalyst for oxidation of allylic C–H bonds in olefins under mild reaction condition. *Res. Chem. Intermed.* **45**, 2441-2455 (2019).

11. Samadi, S., Ashouri, A. & Ghambarian, M. Use of CuO encapsulated in mesoporous silica SBA-15 as a recycled catalyst for allylic C–H bond oxidation of cyclic olefins at room temperature. *RSC Adv.* **7**, 19330-19337 (2017).

12. Samadi, S., Ashouri, A., Kamangar, S. & Pourakbari, F. 2-Aminopyrazine-functionalized MCM-41 nanoporous silica as a new efficient heterogeneous ligand for Cu-catalyzed allylic C–H bonds oxidation of olefins. *Res. Chem. Intermed.* **46**, 557-569 (2020).

13. Samadi, S., Ashouri, A., Majidian, S. & Rashid, H. I. Synthesis of new alkenyl iodobenzoate derivatives via Kharasch-Sosnovsky reaction using *tert*-butyl iodo benzoperoxoate and copper (I) iodide. *J. Chem. Sci.* **132**, 1-9 (2020).

14. Andrus, M. B. & Asgari, D. Asymmetric allylic oxidation with biarylbisoxazoline-copper (I) catalysis. *Tetrahedron* **56**, 5775-5780 (2000).

15. Kawasaki, K.-i. & Katsuki, T. Enantioselective allylic oxidation of cycloalkenes by using Cu (II)-*tris* (oxazoline) complex as a catalyst. *Tetrahedron* **53**, 6337-6350 (1997).

16. Andrus, M. B. & Zhou, Z. Highly enantioselective copper− bisoxazoline-catalyzed allylic oxidation of cyclic olefins with *tert*-butyl *p*-nitroperbenzoate. *J. Am. Chem. Soc.* **124**, 8806-8807 (2002).

17. Ginotra, S. K. & Singh, V. K. Studies on enantioselective allylic oxidation of olefins using peresters catalyzed by Cu (I)-complexes of chiral pybox ligands. *Org. Biomol. Chem.* **4**, 4370-4374 (2006).

18. Tan, Q. & Hayashi, M. Novel *N*, *N*-bidentate ligands for enantioselective copper (I)-catalyzed allylic oxidation of cyclic olefins. *Adv. Synth. Catal.* **350**, 2639-2644 (2008).

19. Wavefunction, Inc. Spartan'14 version 1.1.9 (Irvine, CA).

20. Dennington, R., Keith, T. A. & Millam, J. M. GaussView 6.0. 16. *Semichem Inc.: Shawnee Mission, KS, USA*, (2016).

21. Frisch, M. J. et al. Gaussian, Inc., Wallingford (2009).

22. Seefeldt, L. C. *et al.* Reduction of substrates by nitrogenases. *Chem. Rev.* **120**, 5082-5106 (2020).
